# Supplementary material for: TransHLA: a Hybrid Transformer model for HLA-presented epitope detection
Source: Gigascience. 2025 Feb 27;14:giaf008. doi: 10.1093/gigascience/giaf008 (PMC11878767; doi:10.1093/gigascience/giaf008)
Supplement: giaf008_GIGA-D-24-00176_Original_Submission [file giaf008_giga-d-24-00176_original_submission.pdf]

GigaScience

TransHLA: A Hybrid Transformer Model for HLA-Presented Epitope Detection

--Manuscript Draft--

**Manuscript Number:**	GIGA-D-24-00176
**Full Title:**	TransHLA: A Hybrid Transformer Model for HLA-Presented Epitope Detection
**Article Type:**	Technical Note
**Funding Information:**	An Immune Aging Monitor Based on the Dynamics of TCR Repertoire and Machine Learning (9080002) — Professor Shuaicheng LI
**Abstract:**	The precise prediction of epitope presentation on human leukocyte antigen (HLA) molecules significantly advances the fields of vaccine development and immunotherapy.While conventional HLA-peptide binding affinity prediction softwares can accurately determine the affinity between HLA residues and peptides, they are typically focused on individual or a limited number of alleles, and do not have a universal approach to consider all HLA sites collectively and efficiently filter out invalid peptide segments. Addressing these limitations, we introduce TransHLA, a pioneering software tool designed for epitope prediction across all HLA alleles, a crucial aspect for effective immune functionality. TransHLA integrates Transformer and Residue CNN architectures to analyze peptide sequences and structures comprehensively, thereby surpassing the constraints of prior models that required allele-specific training or exhibited suboptimal performance. By employing the ESM2 large language model for sequence and structure embeddings, TransHLA achieves notable predictive accuracy. Specifically, for HLA class I molecules, it attains an accuracy of 84.72% and an area under the receiver operating characteristic curve (AUC) of 91.95% in IEDB test data. For HLA class II molecules, the accuracy reaches 79.94% with an AUC of 88.14% in IEDB test data. And our case studys, using comprehensive datasets including CEDAR, VDJdb, ImmuneCode, dbPepNeo2.0, and NEPDB, reveal that TransHLA outperforms existing models in both specificity and sensitivity in identifying potential immunogenic epitopes and neoepitopes. These advancements underscore TransHLA's potential to significantly enhance vaccine design and immunotherapy through the efficient identification of broadly reactive peptides. Our resources, including data and code, are publicly accessible at https://github.com/SkywalkerLuke/TransHLA
**Corresponding Author:**	Shuaicheng Li<br><br>HONG KONG
**Corresponding Author Secondary Information:**	
**Corresponding Author's Institution:**	
**Corresponding Author's Secondary Institution:**	
**First Author:**	Tianchi LU
**First Author Secondary Information:**	
**Order of Authors:**	Tianchi LU<br>Xueying Wang<br>Wan Nie<br>Miaozhe Huo<br>Shuaicheng LI
**Order of Authors Secondary Information:**	
**Additional Information:**	
**Question**	**Response**

Powered by Editorial Manager® and ProduXion Manager® from Aries Systems Corporation

Are you submitting this manuscript to a special series or article collection?	No
**Experimental design and statistics**<br><br>Full details of the experimental design and statistical methods used should be given in the Methods section, as detailed in our Minimum Standards Reporting Checklist. Information essential to interpreting the data presented should be made available in the figure legends.<br><br>Have you included all the information requested in your manuscript?	Yes
**Resources**<br><br>A description of all resources used, including antibodies, cell lines, animals and software tools, with enough information to allow them to be uniquely identified, should be included in the Methods section. Authors are strongly encouraged to cite Research Resource Identifiers (RRIDs) for antibodies, model organisms and tools, where possible.<br><br>Have you included the information requested as detailed in our Minimum Standards Reporting Checklist?	Yes
**Availability of data and materials**<br><br>All datasets and code on which the conclusions of the paper rely must be either included in your submission or deposited in publicly available repositories (where available and ethically appropriate), referencing such data using a unique identifier in the references and in the "Availability of Data and Materials" section of your manuscript.<br><br>Have you have met the above requirement as detailed in our Minimum	Yes

Powered by Editorial Manager® and ProduXion Manager® from Aries Systems Corporation

Standards Reporting Checklist?	

Manuscript

Click here to access/download;Manuscript;Epitope_Presentation_prediction.

journal title, 2023, pp. 1–12

doi: DOI HERE

Advance Access Publication Date: Day Month Year

Paper

PAPER

TransHLA: A Hybrid Transformer Model for HLA-Presented Epitope Detection

Tianchi LU,[1,†] Xueying Wang,[1,2,†] Wan Nie,[1] Miaozhe Huo[1] and LI Shuaicheng[1,*]

[1]Department of Computer Science, City University of Hong Kong, Kowloon, Hong Kong and [2]Department of Computer Science, City University of Hong Kong (Dongguan), Dongguan523000, China

FOR PUBLISHER ONLY Received on Date Month Year; revised on Date Month Year; accepted on Date Month Year

Abstract

The precise prediction of epitope presentation on human leukocyte antigen (HLA) molecules significantly advances the fields of vaccine development and immunotherapy.While conventional HLA-peptide binding affinity prediction softwares can accurately determine the affinity between HLA residues and peptides, they are typically focused on individual or a limited number of alleles, and do not have a universal approach to consider all HLA sites collectively and efficiently filter out invalid peptide segments. Addressing these limitations, we introduce TransHLA, a pioneering software tool designed for epitope prediction across all HLA alleles, a crucial aspect for effective immune functionality. TransHLA integrates Transformer and Residue CNN architectures to analyze peptide sequences and structures comprehensively, thereby surpassing the constraints of prior models that required allele-specific training or exhibited suboptimal performance. By employing the ESM2 large language model for sequence and structure embeddings, TransHLA achieves notable predictive accuracy. Specifically, for HLA class I molecules, it attains an accuracy of 84.72% and an area under the receiver operating characteristic curve (AUC) of 91.95% in IEDB test data. For HLA class II molecules, the accuracy reaches 79.94% with an AUC of 88.14% in IEDB test data. And our case studys, using comprehensive datasets including CEDAR, VDJdb, ImmuneCode, dbPepNeo2.0, and NEPDB, reveal that TransHLA outperforms existing models in both specificity and sensitivity in identifying potential immunogenic epitopes and neoepitopes. These advancements underscore TransHLA's potential to significantly enhance vaccine design and immunotherapy through the efficient identification of broadly reactive peptides. Our resources, including data and code, are publicly accessible at `https://github.com/SkywalkerLuke/TransHLA`

Key words: Epitope Presentation, Pre-trained language model, Deep Learning

footnote[1]

Introduction

The intricate process of epitope presentation by human leukocyte antigen (HLA) molecules is a cornerstone of the immune system's ability to combat pathogens, neoplasms, and its involvement in the multifaceted arenas of autoimmunity, allergies, and organ transplant rejection [1, 2]. HLA class I and II molecules play a pivotal role in presenting crucial antigen peptides to T cells[3], thereby triggering downstream immune responses.

Due to the extensive polymorphism of HLA molecules, their affinity for a wide range of peptides can vary significantly, posing a challenge for vaccine design in accurately identifying peptides that can bind to HLAs[4, 5, 6]. The burgeoning interest in HLA peptide binding has revealed the presentation of antigenic peptides by over 22,000 HLA alleles. This wealth of information constitutes a substantial database for deep learning models, offering ample resources for their development and training[7].

The first category consists of models that solely employ potential epitope sequences as input and train individually for each HLA allele. These models propose that peptides binding to the same allele typically exhibit hidden similar features. For instance, MHCnuggets [4] employs Long Short-Term Memory (LSTM) networks to process sequential data, leveraging their ability to remember long-range dependencies. On the other hand, DeepSeqPanII [6] integrates the increasingly popular attention mechanism for feature weighting, while still relying on Recurrent Neural Networks (RNNs) as the backbone for feature extraction.

The second category consists of models that require both the epitope sequence and the HLA allele as input in order to make predictions. The combined embedding approach in models like

[1] To whom correspondence should be addressed. Tel: +852 3442-9412; Email: shuaicli@cityu.edu.hk

[†]The authors wish it to be known that, in their opinion, the first two authors should be considered joint First Authors.

© The Author 2023. Published by Oxford University Press. All rights reserved. For permissions, please e-mail: journals.permissions@oup.com

MHCflurry [5] merges the sequence data of HLA alleles and potential epitopes, allowing for a rich feature set that can be learned, especially with large datasets.

However, while these methods can accurately determine the affinity between HLA residues and peptides, they are typically focused on individual or a limited number of alleles, and do not have a universal approach to consider all HLA sites collectively and efficiently filter out invalid peptide segments.

To expedite the precise screening of peptides capable of eliciting broad immune responses, we have introduced TransHLA. TransHLA is founded upon a combination of Transformer [8] and Residue CNN architectures [9], leveraging both the sequence and structural attributes of peptides for assessing the binding potential between peptides and arbitrary sites of HLA-I or HLA-II molecules. HLA utilizes ESM2 [10], a large language model, to encode the sequence features of peptides. TransHLA employs the pre-trained large language model, ESM2, to encapsulate the sequential and structure information characteristics of peptides.

Considering that TransHLA is the first epitope prediction software that does not impose restrictions on HLA alleles, we have selected several state-of-the-art sequence classification models, namely TextCNN [11], TextRCNN [12], DPCNN [13], and RNN-ATTs [14], for benchmarking and comparison purposes. In addition, in the case study, we employed state-of-the-art peptide-HLA allele binding prediction software to perform predictions for all alleles. We then compared these predictions with the results obtained from TransHLA. The comprehensive analysis of the results consistently demonstrated that TransHLA outperformed the other models in general epitope prediction for both HLA-I binding and HLA-II binding.

Materials and methods

Datasets

The datasets used in this work were collected and curated from IEDB [15], CEDAR [16], VDJdb [17], ImmuneCode [18], dbPepNeo2.0 [19] and NEPDB [20]databases. The IEDB database provided the source of our train, validation, test with a 7:2:1 ratio. The other four databases—CEDAR, VDJdb, ImmuneCode, and dbPepNeo2.0 were utilized exclusively for external test to assess the generalizability of our models. The NEPDB were utilized for assessing the performance on the neoepitope prediction. Our particular emphasis was on epitopes originating from human hosts that exhibited a positive outcome in Ligand elution/Mass spectrometry assays. For epitopes presented by HLA-II, the peptide length varied between 13 and 21 amino acids [21], whereas for epitopes presented by HLA-I, the peptide length was within the range of 8 to 14 amino acids [21].

To construct negative samples for both HLA-II and HLA-I datasets, the negative samples were derived by using diamond [22] to blast the positive peptide sequences against the non-redundant (nr) database [23], recovering the proteins from which the sequences originated. From these proteins, random fragments excluding the positive sequences were selected to ensure non-overlap. In this way, we obtained negative samples that are representative of the potential peptide repertoire but do not include the known positive epitopes. Sequence redundancy was removed using CD-HIT [24] with a threshold of 0.8. Finally, we obtained a balanced dataset consisting of 312,245 positive samples and an equal number of negative samples for epitopes presented by HLA-II. Similarly, for epitopes presented by HLA-I, we selected 459,442 samples for both positive and negative samples. The details can be found in Table 1.

Table 1. The number of samples on training datasets and independent test datasets

Datasets	Types	Count
HLA-I Epitopes	Positive	459,442
	Negative	459,442
HLA-II Epitopes	Positive	312,245
	Negative	312,245

Pre-trained Embeddings for Sequence and Structure

Pre-trained protein language models[25, 10, 26] have been extensively applied in various tasks, such as protein classification, by providing intricate representations of protein sequences [27, 28, 29]. Additionally, AlphaFold2 [30] and ColabFold [31] have set high standards in protein structure prediction.

In our approach, we address the issue of HLA-I binding epitopes with lengths less than 14 and HLA-II binding epitopes with lengths less than 21 by padding the end with ones. This padding technique ensures that the sequences have the required lengths. Subsequently, we utilize the ESM2 protein language model to extract sequence embeddings and predict structure embeddings for these epitopes[10].

The Architectures of the Deep Learning Model

The Transformer module

To enhance the extraction of global features, we incorporated the Transformer encoder module [8, 32], which utilizes inputs in the form of pre-trained sequence features extracted by ESM2, represented as a $\mathbf{E} \in \mathbb{R}^{1280 \times peptide\_length}$ matrix. The module leverages a multi-head attention mechanism to facilitate effective global feature extraction.

Within each attention head, three key components are involved: Q (query), K (key), and V (value). Q represents the current position being attended, while the K and V represent other positions in the peptide sequence. By computing the attention weights between the Q and K, the model determines the importance of each position and assigns higher weights to more relevant positions. The values are then combined based on these attention weights to generate the output representation, and the scaled dot-product attention is calculated as:

$$\text{Attention}(Q, K, V) = \text{softmax}\left(\frac{QK^T}{\sqrt{d_k}}\right)V \tag{1}$$

d_k is the dimension of the key vectors.

The multi-head attention is achieved through a series of operations to transform the input vectors Q, K, and V for h times (where h is the number of heads). Each transformed vector undergoes scaled dot-product attention independently. Finally, the attention outputs are concatenated and further transformed. This process can be expressed as follows:

$$\begin{aligned} \text{MultiHead}(Q, K, V) &= \text{Concat}(\text{head}_1, \ldots, \text{head}_h)W_O \\ \text{where head}_j &= \text{Attention}(QW_{Q_j}, KW_{K_j}, VW_{V_j}) \end{aligned} \tag{2}$$

In the given equation, W_{Q_j}, W_{K_j}, and W_{V_j} represent weight matrices for each head corresponding to the Q, K,

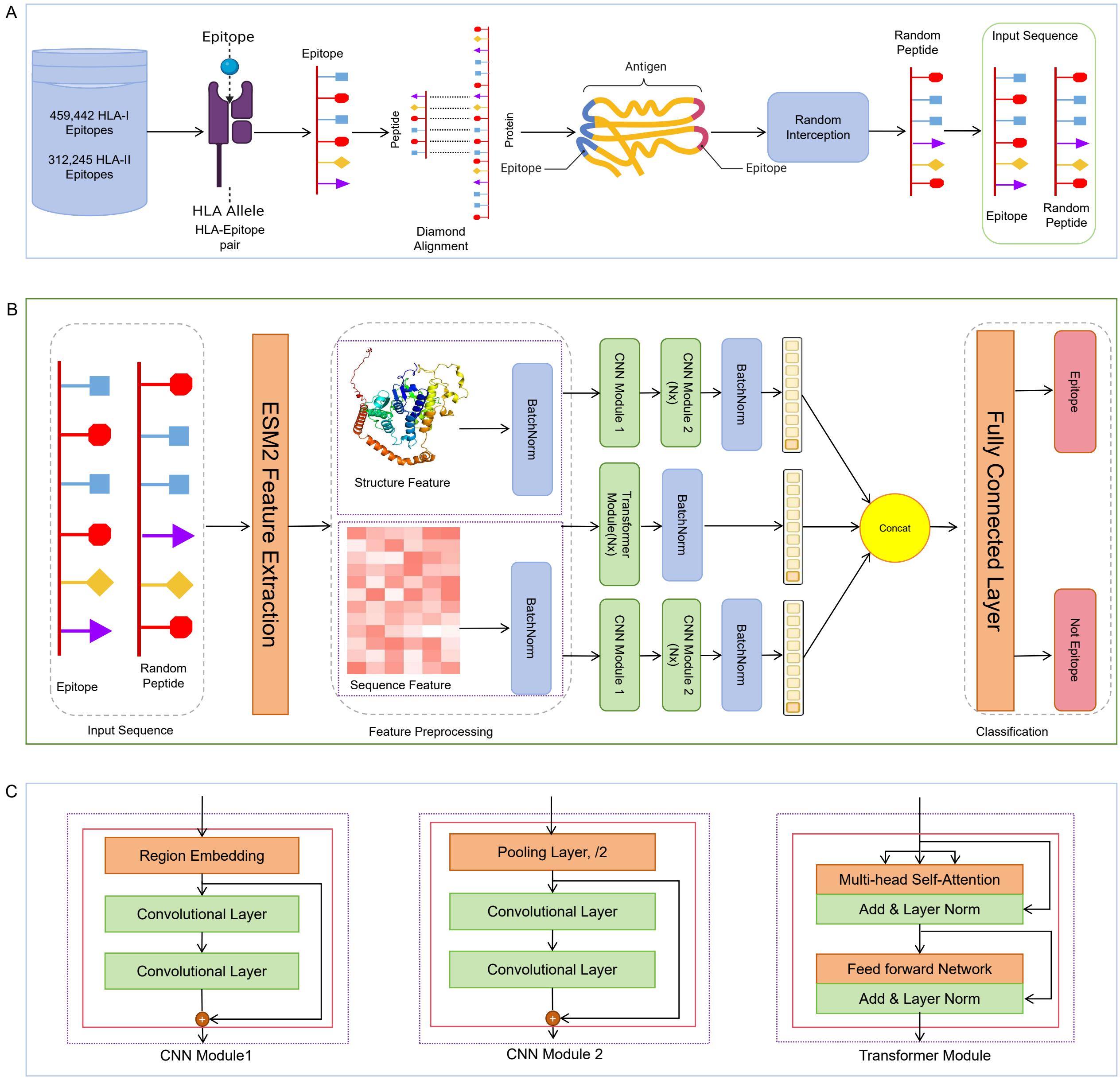

Fig. 1. Overview of data acquisition and predictive modeling using TransHLA. (A) Data Acquisition: The dataset, derived from the IEDB, features a variety of peptide sequences that bind to HLA class I or II molecules. For negative sample generation, Non-overlapping random peptide fragments were sourced by matching positive peptides to their originating proteins through sequence alignment. THen, the Non-overlapping random peptide fragments were processed with CD-HIT to achieve a reduced redundancy, resulting in the final set of negative samples.(B) With ESM2's advanced modeling capabilities, we generated high-dimensional sequence embeddings for the peptides associated with both HLA classes. Concurrently, structural insights were obtained through ESM2's contact map predictions, yielding structure embeddings. These two distinct yet complementary sets of embeddings were crafted to capture the intricate nature of peptide-HLA interactions. describes the process from data input to epitope presentation prediction. (C) The architecture of the different modules, including the CNN Module 1, CNN Module 2 and the Transformer module.

and V vectors, respectively. W_O denotes the weight matrix for the output. This formulation allows for weight sharing across heads, reducing redundancy and promoting a more compact representation.

The CNN module

To enhance feature extractions, TransHLAs employs two structurally identical CNNs, each consisting of a CNN module 1 for region embedding, followed by multiple layers of CNN module 2. These modules process both the pre-trained sequence features **E** and the contact map structural features extracted by ESM2, with the contact map being a

symmetric matrix $\mathbf{S} \in \mathbb{R}^{peptide\_length \times peptide\_length}$. Residual connections are implemented between each module to prevent gradient vanishing and ensure effective training of the deep network structure.

CNN Module 1

It first applies a text region embedding layer to get a dense representation of sequences:

$$\mathbf{x} = \text{RegionEmbed}(\mathbf{E}) \tag{3}$$

where $\mathbf{x} \in \mathbb{R}^{M \times D}$ and M is the number of text regions and D is the region embedding dimension.

This is followed by a convolution block, which contains two convolutional layers each with 256 feature maps:

$$\mathbf{z}_1 = \text{ConvBlock}(\mathbf{x}) \tag{4}$$

where $\mathbf{z}_1 \in \mathbb{R}^{M \times 256}$.

CNN Module 2

Following the CNN module 1, the CNN module 2 commences with a pooling layer that reduces the length of the feature map to half of its original size. Subsequently, CNN module 2 employs a convolutional block with the same structure as the one in CNN module 1, featuring an isometric convolutional layer with 256 channels.

$\mathbf{z}_1$:

$$\mathbf{x}_1 = \text{Downsample}(\mathbf{z}_1) \tag{5}$$

where $\mathbf{x}_1 \in \mathbb{R}^{M/2 \times 256}$.

$$\mathbf{z}_2 = \text{ConvBlock}(\mathbf{x}_1) \tag{6}$$

where $\mathbf{z}_2 \in \mathbb{R}^{M/2 \times 256}$.

This is repeated for L times, with the downsampling layer and l-th CNN Modules being:

$$\mathbf{x}_l = \text{Downsample}(\mathbf{z}_l) \tag{7}$$

$$\mathbf{z}_{l+1} = \text{ConvBlock}(\mathbf{x}_l) \tag{8}$$

where $\mathbf{z}_{l+1} \in \mathbb{R}^{M/2^l \times 256}$.

After the final Downsample layer, we add a batchnorm layer, which helps reduce internal covariate shift and acts as a regularization technique.

$$\mathbf{z}_{l+1} = \text{BatchNorm}(\mathbf{z}_{l+1}) \tag{9}$$

The TIM Loss

The TransHLA framework is developed based on a modified TIM loss function, as introduced by Boudiaf et al. (2020) [33], which merges conventional cross-entropy with a mutual information component, tailored for empirical analysis. we address the empirical mutual information within dataset X (comprising amino acid sequences) linked to their respective outcomes Y (which now signify epitope presentation). The initial factor is the empirical conditional entropy of the outcomes conditioned on the data, notated as $\widehat{\mathcal{H}}(Y|X)$. The next factor is the empirical marginal entropy of the outcomes, notated as $\widehat{\mathcal{H}}(Y)$. To further calibrate the binary classification process, the cross-entropy loss, indicated as CE, between the model's predictions and the actual outcomes is incorporated. The formulation for these components is defined as follows:

$$\begin{aligned} \widehat{\mathcal{H}}(Y) &:= -\sum_{k=1}^{K} \widehat{p}_k \log \widehat{p}_k \\ \widehat{\mathcal{H}}(Y \mid X) &:= -\frac{1}{|X|}\sum_{i \in X}\sum_{k=1}^{K} p_{ik}\log(p_{ik}) \\ \text{CE} &:= -\frac{1}{|X|}\sum_{i \in X}\sum_{k=1}^{K} y_{ik}\log(p_{ik}) \end{aligned} \tag{10}$$

Let $|X|$ denote the total count of sequences within the dataset, with i being the sequence identifier in X, and T representing the possible outcome categories. The variable p_{it} is the predicted likelihood of the i-th amino acid sequence being classified within the t-th category. The binary variable y_{it} is used to indicate if the i-th sequence is actually categorized under class t. We assign $T = 2$ for this model since the task at hand is a binary classification problem.

The final loss function for TransHLA is defined as:

$$\widehat{\mathcal{L}}(X;Y) := \text{CE} - \widehat{\mathcal{H}}(Y) + \alpha\widehat{\mathcal{H}}(Y \mid X) \tag{11}$$

Where α is hyperparameter that determine the rate of convergence for each term in the loss function. In experiments, we set $\alpha = 0.04$, considering the standard cross-entropy loss and standard mutual information.

By selecting these particular hyperparameter values, we maintain fairness and impartiality in TransHLA's training process.

Performance evaluation

In the Benchmark Results with other sequence classification models, we employed the aforementioned metrics, including accuracy (ACC), Recall, F1-score (F1), and Matthews Correlation Coefficient (MCC), to evaluate the performance of TransHLA.

$$ACC = \frac{TP + TN}{TP + FP + TN + FN} \tag{12}$$

$$Recall = \frac{TP}{TP + FN} \tag{13}$$

$$F1 = \frac{2TP}{2TP + FN + FP} \tag{14}$$

$$MCC = \frac{TP \times TN - FP \times FN}{\sqrt{(TP + FP) \times (TP + FN) \times (TN + FP) \times (TN + FN)}} \tag{15}$$

Additionally, in the Comparison Results with other HLA-epitope binding software across extensive datasets, we augmented our assessment with two further metrics, Precision and Specificity. These additional indicators highlight how our methodology has overcome the limitations commonly associated with traditional affinity-binding software, particularly the tendency to incorrectly classify negative samples as positive.

$$Precision = \frac{TP}{TP + FP} \tag{16}$$

$$Specificity = \frac{TN}{TN + FP} \tag{17}$$

Where TP (true positives) represents the number of correctly identified true epitope, TN (true negatives) represents the

Table 2. Benchmark Results with other sequence classification models

Type	Method	ACC (%)	F1(%)	Recall(%)	MCC	AUC (%)
HLA-I	TransHLA	**84.72**	**84.59**	83.92	**0.69**	**91.95**
	TextCNN	81.63	79.61	83.50	0.63	89.37
	TextRCNN	81.21	76.65	83.78	0.64	87.62
	DPCNN	83.75	83.76	**83.96**	0.68	90.97
	RNN-ATTs	81.17	82.27	81.46	0.63	87.98
HLA-II	TransHLA	**79.94**	**81.07**	**86.19**	**0.60**	**88.14**
	TextCNN	73.26	73.49	72.64	0.47	80.64
	TextRCNN	70.96	72.02	69.28	0.42	78.21
	DPCNN	77.41	77.91	75.98	0.55	85.30
	RNN-ATTs	69.04	69.83	67.89	0.38	75.81

number of correctly identified the normal peptide, The FP (false positives) represents the number of instances where normal peptides were incorrectly identified as epitopes , and FN (false negatives) represents the number of instances where epitopes were incorrectly identified as normal peptides.

In addition to these metrics, we also utilize Receiver Operating Characteristic (ROC) and Precision-Recall (PR) curves as significant evaluation tools for classification accuracy. The Area Under the ROC Curve (AU-ROC) and the Area Under the Precision-Recall Curve (AU-PRC) values quantify the overall performance by measuring the area beneath the ROC and PR curves, respectively.

Results

Benchmark Results with other sequence classification models

Since this paper focuses on the epitope presentation classification problem, a corresponding software for comparison is not yet established. We conducted comparison experiments on independent test sequences from IEDB including 92,347 HLA-I binding epitopes, 65,105 HLA-II binding epitopes and 157,879 random sequences with the state-of-the-art sequence classification models, including TextCNN [11], TextRCNN [12], DPCNN [13], and RNN-ATTs [14].

The performance metrics are presented in Table 2. TransHLA exhibits enhanced performance in classifying HLA-I epitopes across four metrics, including ACC, F1, Recall, MCC and AUC. The corresponding values for TransHLA are 0.847, 0.846, 0.694, and 0.920, respectively. In comparison, the second-ranked software obtains scores of 0.838, 0.838, 0.68 and 0.910 for the same metrics, respectively. Figure 2A and 2C illustrate the ROC and PR curves of the compared models.

HLA-II binding predictions are known to be more complex compared to HLA-I predictions. [34, 35, 36, 37] Consequently, the performance of the models, in general, is inferior in terms of HLA-II binding metrics of other models, with an average decrease of 9.3% in ACC, 7.26% in F1, 11.73% in Recall, 0.19 in MCC, 9.00% in AUC. However, even in the challenging task of HLA-II binding prediction, TransHLA demonstrates robust classification performance. Compared to the values achieved in HLA-I binding prediction, TransHLA only experiences a decrease of 4.78%in ACC, 3.52% in F1, 0.09 in MCC, and 3.81% in AUC. Remarkably, TransHLA achieves an increase of 2.27% in Recall. Furthermore, in the prediction of HLA-II epitope binding, TransHLA demonstrates superior performance across all evaluation metrics. Compared to the next best-performing models, TransHLA achieves an improvement of 0.25 in ACC, an enhancement of 0.316 in the F1 Score, a boost of 1.02 in Recall, and a substantial improvement of 0.548 in MCC.

Comparison Results with other HLA-epitope binding software in the case study

We employed our software along with various state-of-the-art epitope-HLA binding prediction tools, including Mhcflurry [5], DeepSeqPanII [6], and Mhcnuggets [4] to evaluate their accuracy in correctly identifying sequence as potential epitopes from CEDAR [16], VDJdb [17], ImmuneCode [18], and dbPepNeo2.0 [19] datasets. Our analysis yielded a total of 21,387 HLA-I binding epitopes and 3,580 HLA-II binding epitopes in the case study. Our criterion for deeming a peptide as a presentation-worthy epitope is that it must exhibit binding affinity to at least one major HLA allele. Mhcflurry contains 11,576 HLA-I alleles, Mhcnuggets contains 118 HLA-II alleles and 106 HLA-I alleles, and DeepSeqPanII contains 61 HLA-II alleles. And the details information of alleles used in each tool can be found in the Supplementary File.

To better explore the ability of different models to distinguish between epitope and non-epitope sequences, we conducted two experimental scenarios. In the first scenario, we added random sequences in the same quantity as the identified persistent epitopes. In the second scenario, we added random sequences at four times the number of the persistent epitopes. The results of these two experiments are presented in Table 3 and Table 4, respectively. For the prediction of HLA-I binding, we compared the performance of the TransHLA against Mhcflurry and Mhcnuggets. In the prediction of HLA-II binding, we compared the performance of the TransHLA, DeepSeqPanII, and Mhcnuggets. The details of the parameters used in the mentioned models can be found in the Supplementary File.

In the first experimental scenario, TransHLA and Mhcflurry demonstrated good performance in predicting HLA-I binding. Among the three models, TransHLA achieved the highest ACC of 83.09%, precision of 85.22%, and specificity of 86.11%, followed by Mhcflurry with 82.96%, 81.95%, and 81.38% in the same metrics. Besides, TransHLA, in conjunction with the Mhcflurry, obtained the highest MCC of 0.66 in this experiment. Mhcflurry had the highest F1-score of 83.24%, while TransHLA obtained the second highest F1-score of 82.96%. Upon closer examination of the F1 score metrics (Figure 3A), it was observed that TransHLA attained the highest true negative (TN) rate of 86.1%, which surpassed that of MhcFlurry at 81.4%. Conversely, MhcFlurry exhibited the highest true positive (TP) rate at 84.6%. This differential performance indicates that TransHLA demonstrates a more robust capability in filtering out noise during epitope

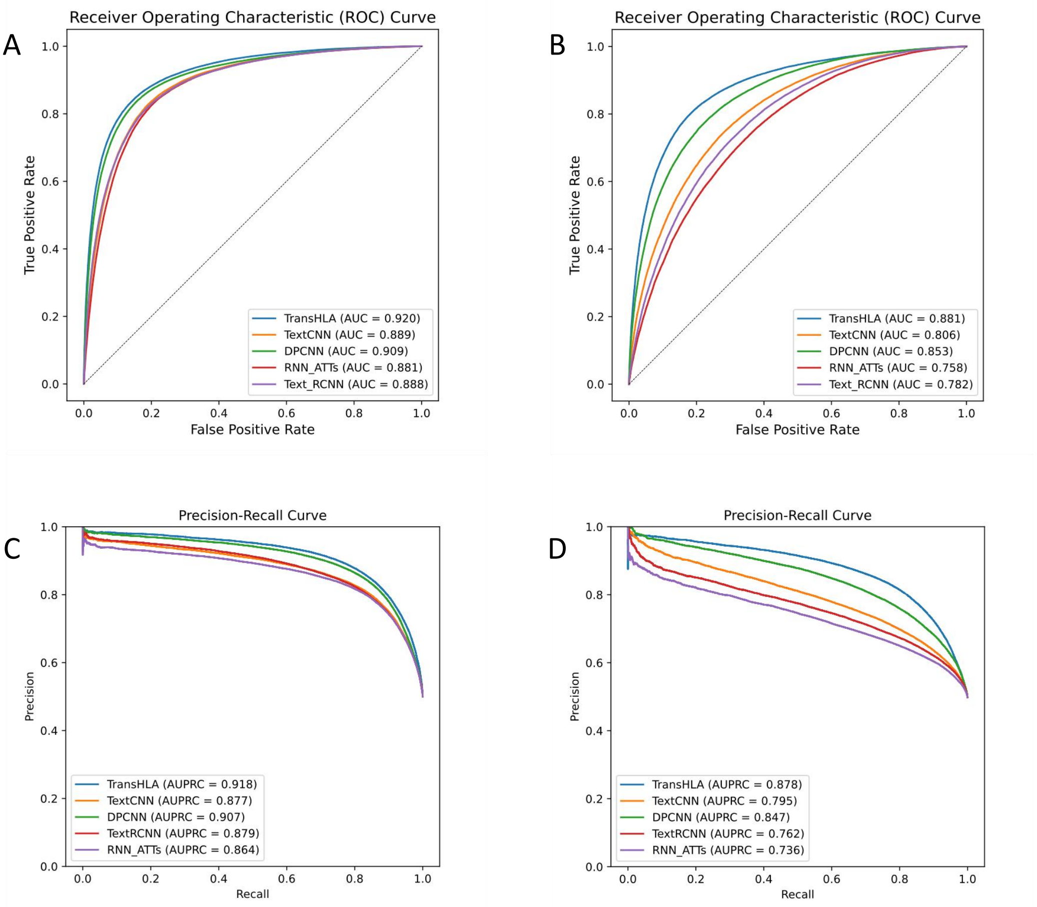

Fig. 2. This figure evaluates TransHLA's epitope prediction capabilities, benchmarked against other models using two key statistical metrics: AUROC and AUPRC. Subfigures (A) and (C) examine HLA-I class performance, with (A) showing AUROC and (C) presenting AUPRC. The graphs demonstrate TransHLA's proficiency in distinguishing between epitope and non-epitope peptides for HLA-I, where AUROC indicates its discriminative power and AUPRC reflects the precision-recall trade-off. Subfigures (B) and (D) extend the analysis to HLA-II, with (B) displaying AUROC and (D) illustrating AUPRC. The performance depicted emphasizes TransHLA's effectiveness in identifying HLA-II epitopes, highlighting its ability to differentiate between classes under imbalanced distributions. Together, the subfigures demonstrate TransHLA's advantage over conventional models in epitope prediction for both HLA classes.

identification. In the prediction of HLA-II binding, TransHLA showed excellent performance, achieving the highest scores in ACC (66.88%), F1 (64.08%), MCC (0.34), Precision (66.99%), and Specificity (74.67%). In contrast, while Mhcnuggets achieved a high recall of 99.84% in HLA-I binding and 89.69% in HLA-II binding, its precision was only 50.04%, and 19.83% respectively, indicating that it misjudged a large number of negative samples as epitopes. This will cause a lot of noise to be mixed into the screening results. And DeepSeqPanII gets poorer performance than Mhcnuggets in HLA-II binding. Additionally, DeepSeqPanII performed worse than Mhcnuggets in HLA-II binding. In the second experimental scenario, as illustrated in Table 4, the performance characteristics of TransHLA and MhcFlurry exhibited a high degree of stability, demonstrating strong robustness. Specifically, TransHLA attained a TN rate of 85.9%, outperforming MhcFlurry's TN rate of 81.5% (Figure 3B). Conversely, Mhcflurry achieved a superior true TP rate of 84.6% compared to TransHLA's TP rate of 80.1%. When the negative sample size was four times greater than the positive sample, the F1 score of TransHLA (67.73%) surpassed that of MhcFlurry (65.38%), indicative of TransHLA's enhanced resilience to the influence of increased noise in the data. In contrast, the performance of Mhcnuggets and DeepSeqPanII exhibited a significant decline in this particular scenario.

Good performance achieved in NeoEpitope prediction by TransHLA

To validate the effectiveness of TransHLA in NeoEpitope prediction, we employed newly identified antigens with immunogenicity verified by TCR experiments from NEPDB [20] as positive samples and compared our software with NetMHCpan [21], a widely used tool for NeoEpitope prediction in the industry. In NEPDB, we collected a total of 139 neoantigens presented by HLA-I alleles and 11 neoantigens presented by HLA-II alleles. Additionally, we used a 1:1 ratio of randomly selected negative samples to form our final dataset.

The results of the comparison between TransHLA and NetMHCpan are presented in Figure 3 E, F and Table 4. We observed that our model demonstrated superior

Table 3. Comparison Results with other HLA-epitope binding softwares in One-time negative Case Study

Type	Method	ACC (%)	F1(%)	Recall(%)	MCC	Precision(%)	Specificity (%)
HLA-I	TransHLA	**83.09**	82.56	80.06	**0.66**	**85.22**	**86.11**
	Mhcflurry	82.96	**83.24**	84.57	0.66	81.95	81.38
	Mhcnuggets	50.07	66.67	**99.87**	0.02	50.04	0.29
HLA-II	TransHLA	**66.88**	**64.08**	59.09	**0.34**	**66.99**	**74.67**
	DeepSeqPanII	49.68	49.89	50.10	-0.00	49.68	48.10
	Mhcnuggets	50.13	64.26	**89.69**	0.00	50.07	10.59

Table 4. Comparison Results with other HLA-epitope binding softwares in Four-time negative Case Study

Type	Method	ACC (%)	F1(%)	Recall(%)	MCC	Precision(%)	Specificity (%)
HLA-I	TransHLA	**84.75**	**67.73**	80.06	**0.59**	**58.70**	**85.92**
	Mhcflurry	82.08	65.38	84.57	0.57	53.28	81.46
	Mhcnuggets	20.18	33.40	**99.87**	0.01	20.02	0.10
HLA-II	TransHLA	**71.14**	**45.08**	59.24	**0.28**	**36.39**	**74.12**
	DeepSeqPanII	48.59	28.24	50.59	-0.01	19.59	48.10
	Mhcnuggets	25.36	32.00	**89.80**	-0.01	19.83	9.20

performance in various metrics. In the prediction of HLA-I presented NeoEpitopes, TransHLA achieved an accuracy of 90.65%, surpassing NetMHCpan4.1b's accuracy of 85.25%. Furthermore, although NetMHCpan4.1b achieved a recall of 98.56%, its specificity was only 71.94%. This finding highlights that, like other conventional software, NetMHCpan4.1b is prone to accumulating errors through the misclassification of negative samples as positive when employed for universal, unrestricted HLA site recognition of peptides. In contrast, TransHLA maintained a specificity of 87.77% while also achieving a high recall of 93.52%.

Regarding the prediction of HLA-II presented NeoEpitopes, we observed that the performance of NetMHCIIpan4.3b was unsatisfactory, with an accuracy of only 59.09%. It also accumulated a significant number of errors, as evidenced by its specificity of 54.55% and recall of 63.64%. In contrast, TransHLA exhibited favorable performance across various metrics on the NEPDB dataset, achieving an accuracy of 81.82%. These results indicate that TransHLA exhibits excellent performance in accurately predicting new antigens in a general context.

Ablation study

To evaluate the contribution of different components of TransHLA to its performance on test data, we conducted an ablation study on five variants: without transformer module, omitting structure pre-trained embedding, removing sequence pre-trained embedding, deleting CNN module, without any pre-trained embedding and changing TIM loss to Cross Entropy loss. The performance metrics for different modules across both HLA-I and HLA-II models are illustrated in the Supplementary File.

Based on the comparisons conducted, it is evident that the sequence embedding approach is significantly more effective in capturing the peptide features, achieving 83.78% and 79.68% accuracy in HLA-I and HLA-II, respectively.The CNN modules, due to their difference in global feature extraction compared to pretrained transformers, contribute substantially to performance enhancement, achieving 83.43% and 79.38% accuracy in HLA-I and HLA-II. Additionally, other modules also play their respective roles in the overall efficacy of the system. This is corroborated by ablation studies, which demonstrate that each module contributes positively to the model's predictive capabilities.

The experimental environment of TransHLA is: Python 3.10 programming, 64.0 GB RAM, one GeForce RTX 3080(10G) GPU.

TransHLA extracts a high-quality peptide embedding in low-dimension

To assess the feature extraction capability of the model, we undertook dimension reduction and visualization of the penultimate layer features derived from the self-trained models outlined in Section 3.1, along with random embeddings and the TransHLA embeddings. The PCA [38] layouts of the learned representations for HLA-I epitopes binding prediction (Figure 4).

When comparing with random embedding, both DPCNN (Figure 4 I-D, II-D) and TransHLA (Figure 4 I-H, II-H)demonstrate superior embedding effects on HLA-I and HLA-II binding epitopes, thereby exhibiting distinct discrimination between positive and negative samples. However, in the low-dimensional visualization of DPCNN, a considerable number of positive and negative samples overlap at the junction, while TransHLA exhibits a more apparent boundary in comparison.

Flexibility Patterns of Epitopes in Antigen Presentation

The structural dynamics of peptides play a critical role in antigen processing and presentation. [39, 40] In this analysis, we investigate the calculated flexibility differences between epitopes and non-epitopic peptide regions presented by both HLA-I and HLA-II from datasets mentioned in Section 3.1 and Section 3.2. Moreover, we compare the flexibility between true positive and true negative samples predicted by TransHLA. The biopython [41] is used for the flexibility calculations.

Through rigorous statistical analyses conducted on our test dataset, we made an intriguing observation regarding the flexibility of epitopes and non-epitopes. Our findings demonstrate that epitopes consistently exhibit lower flexibility in comparison to non-epitopes (Figure 5 A left, B left) with a statistically significant p-value of less than 1×10^{-6}.

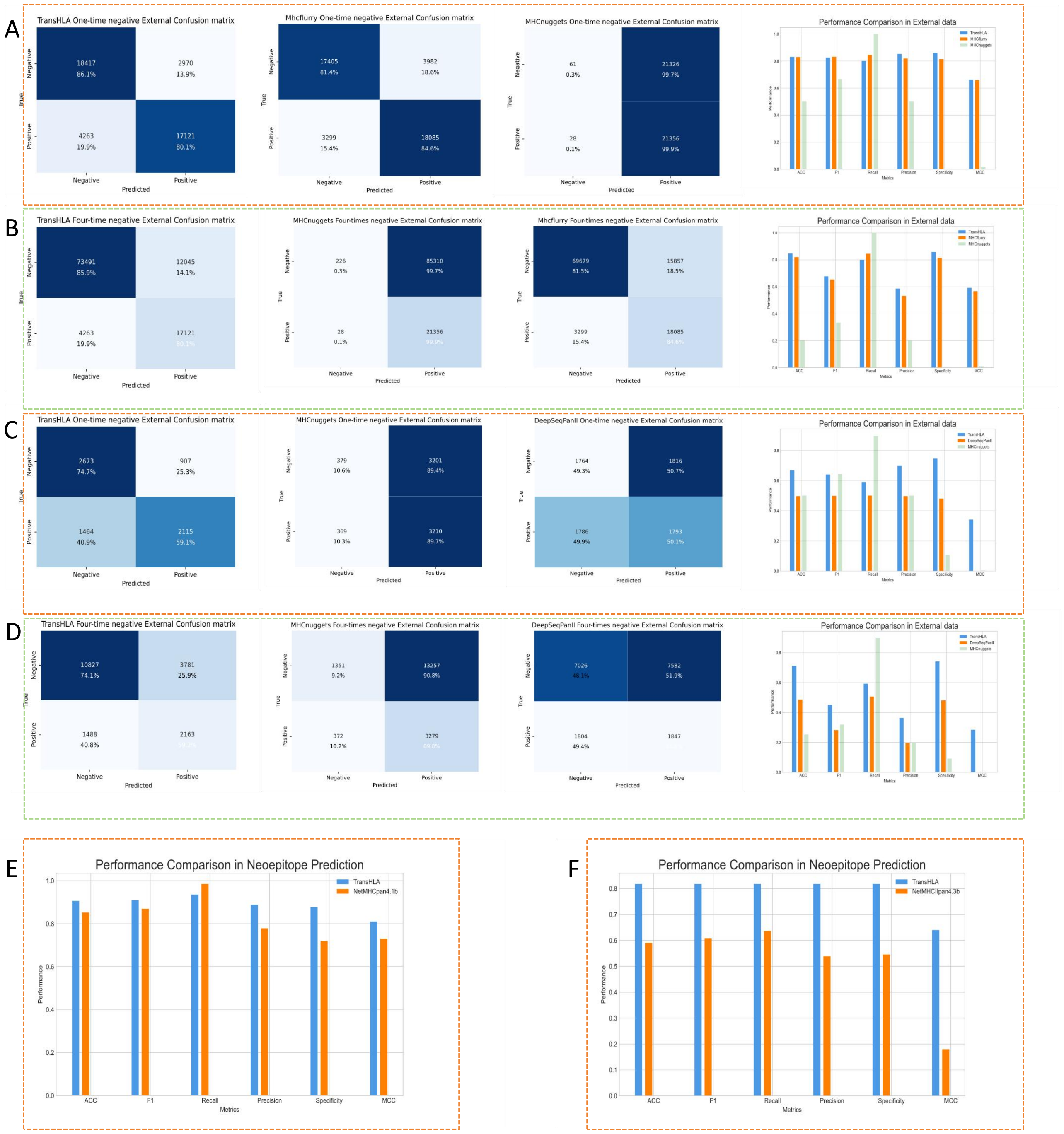

Fig. 3. This figure provides a comparative analysis of epitope and neoepitope prediction performance for HLA-I and HLA-II classes using our software versus other leading tools. Confusion matrices and performance metrics (accuracy, precision, recall, F1 score) are shown in bar charts for balanced datasets (Subfigures A and C) and negative-skewed (four-time negative) datasets (Subfigures B and D). Subfigure E and Subfigure F specifically illustrate the performance metrics for neoepitope prediction in HLA-I and HLA-II classes, respectively. The results highlight our software's accuracy and robustness under varying class imbalances, with a particular emphasis on its efficacy in predicting neoepitopes.

Table 5. Comparison Results with NetMhcpan in NeoEpitope Prediction Case Study

Type	Method	ACC (%)	F1(%)	Recall(%)	MCC	Precision(%)	Specificity (%)
HLA-I	TransHLA	**90.65**	**90.91**	93.52	**0.81**	**88.43**	**87.77**
	NetMHCpan4.1b	85.25	86.98	**98.56**	0.73	77.84	71.94
HLA-II	TransHLA	**81.82**	**81.82**	**81.82**	**0.64**	**81.82**	**81.82**
	NetMHCIIpan4.3	59.09	60.87	63.64	0.18	53.86	54.55

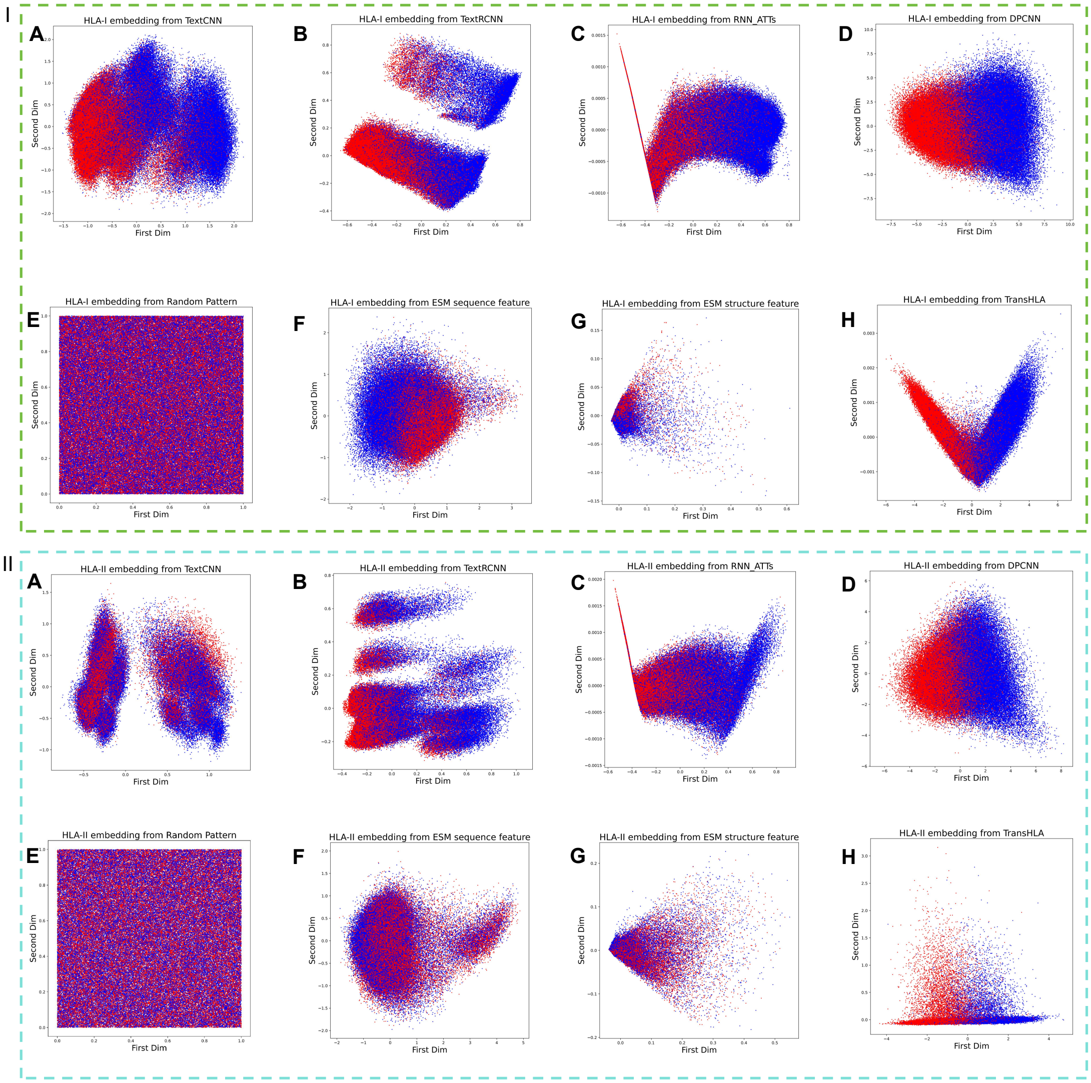

Fig. 4. This figure displays the PCA-based 2D feature space distribution of HLA-I and HLA-II epitope presentation test sets. The space is split into two regions: Region I for HLA-I epitopes and Region II for HLA-II epitopes. Each region shows positive samples as blue dots and negative samples as red dots, with representations from various model embeddings (A-H) including Random Pattern, TextCNN, TextRCNN, RNN-ATTs, DPCNN, random initial, ESM2 pre-trained sequence, structure pre-trained, and TransHLA.

Furthermore, we also examined the flexibility of true positive and true negative samples predicted by TransHLA and found a similar trend (Figure 5 A, B) with a p-value of less than 1×10^{-6} (Figure 5 A right, B right). Upon conducting a comparative analysis of Figure 6C and Figure 5 C and D, a notable divergence was observed when testing the model on the case study dataset. Specifically, epitopes associated with HLA Class I consistently exhibited their characteristic lower flexibility, as evidenced by a statistically significant p-value of less than 1×10^{-6}. Conversely, in the case of epitopes associated with HLA Class II, this characteristic was not maintained, as indicated by a p-value of 2.5×10^{-1}. Consequently, the performance of TransHLA declined compared to the benchmark reported in Section 3.1.

Further exploring the positive epitope samples, which include True Positives (TP) and False Negatives (FN), as well as the negative epitope samples, which include True Negatives (TN) and False Positives (FP), we made an

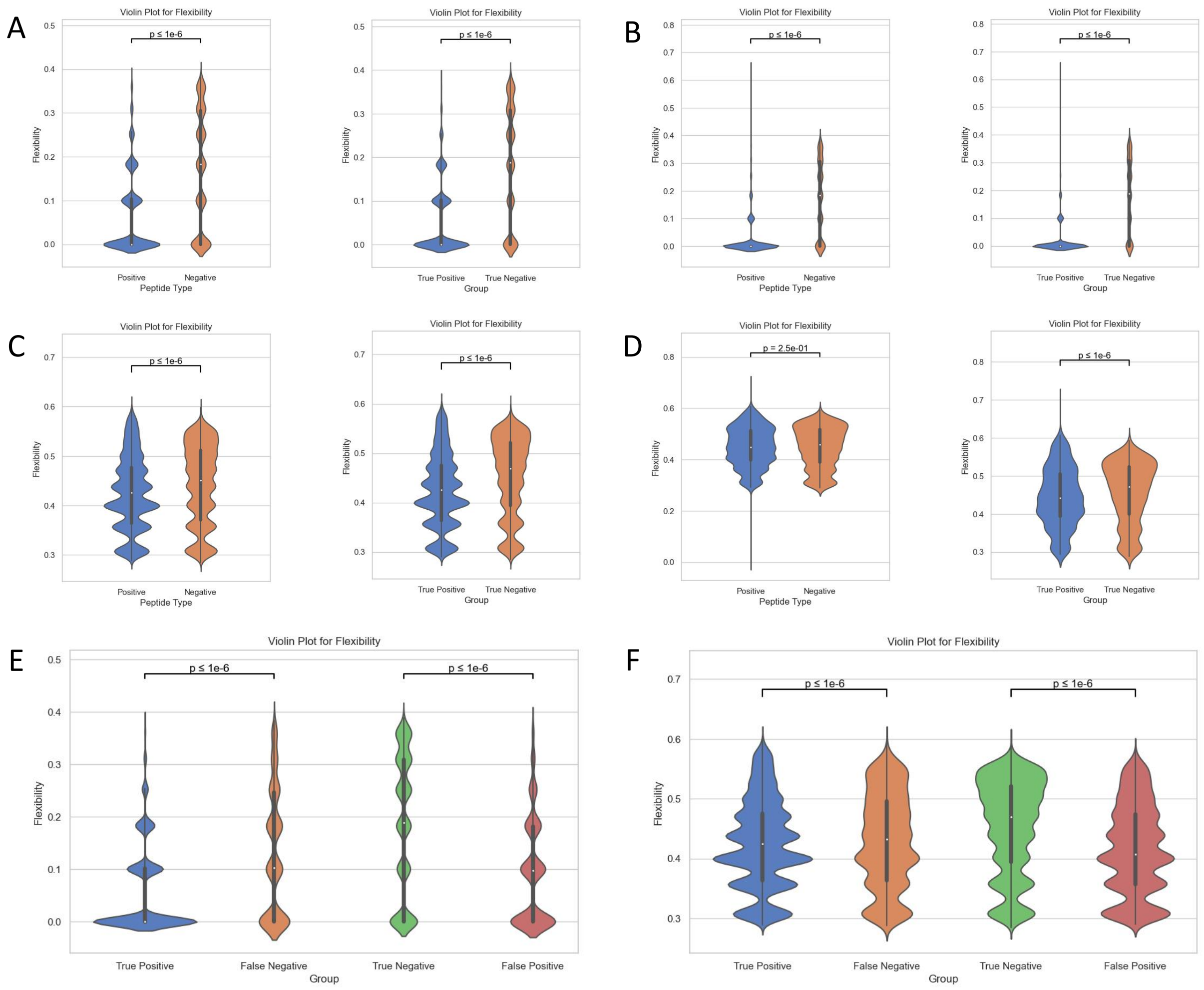

Fig. 5. This comprehensive figure presents a series of violin plots illustrating the 'Flexibility' chemical property of peptides across various sample subsets for HLA-I and HLA-II molecules. Subfigure (A) delineates the flexibility distribution in independent test samples for HLA-I, separated into positive and negative samples, with each subgroup's statistical significance assessed via t-tests and annotated with corresponding p-values. Subfigure (B) mirrors this setup for HLA-II independent test samples, highlighting the comparative flexibility distributions. The external dataset distributions for HLA-I and HLA-II are respectively showcased in subfigures (C) and (D), emphasizing the metric's external validity. Subfigures (E) and (F) delve deeper, contrasting the flexibility of true positives and false negatives against true negatives and false positives within HLA-I and HLA-II datasets, respectively. These plots collectively elucidate the nuanced relationship between peptide flexibility and its propensity for epitope presentation, with the violin plots' breadth reflecting the data density and providing an intuitive visualization of the flexibility spectrum across the tested classifications.

intriguing observation (Figure 5 E, F). There is a significant distribution gap in flexibility between the correctly classified samples (TP and TN) and the incorrectly classified samples (FP and FN) predicted by TransHLA ($p - value \leq 1 \times 10^{-6}$). Interestingly, the incorrectly classified samples (FN and FP) exhibit relatively similar flexibility distributions. This similarity in epitope characteristics between the FN and FP samples likely contributes to the high difficulty in accurate prediction.

Conclusion and Discussion

In this work, we introduced TransHLA, a pre-trained language model-based deep learning framework for predicting epitopes presented by both HLA-I and HLA-II. TransHLA uses a large language model to extract the structural features and text feature ports of candidate sequences and then uses CNN and Transformer modules to process features. On the benchmark dataset, TransHLA was shown to outperform cutting-edge sequence classification models in predicting both HLA-I and HLA-II binding epitopes. Additionally, the case study found that TransHLA substantially outperformed state-of-the-art peptide-HLA-specific binding methods under different noise levels.

In comparison to traditional epitope-HLA binding methods that necessitate knowledge of both the epitope and HLA allele information, TransHLA provides users with the capability to perform epitope screening without the restrictive requirement of knowing the specific HLA alleles. TransHLA efficiently filters out non-epitope sequences and achieves higher accuracy compared to conventional methods. In a general Neoeptope dataset verified by TCR experiments, TransHLA achieves an accuracy of 90.65% for HLA-I epitopes and 81.82% for HLA-II epitopes. These results demonstrate TransHLA's efficacy in

accurately identifying potential epitopes, making it a promising alternative for filtering potential epitopes in vaccine design.

Code availability

We provide the source code of TransHLA including model training, inference and evaluation, which is freely available at `https://github.com/SkywalkerLuke/TransHLA`.

Data availability

Our datasets used in this paper is collected from [15, 16, 17, 18, 19, 20]. We also submit it along with our source code of TransHLA.

Supplemental information

.

Competing interests

The authors declare no competing interests.

Author contributions statement

SCL supervised the project, designed the initial study, and revised the manuscript. TCL and XYW designed the study, implemented the code, and wrote the manuscript. NW implemented the tests and discussed the project. MZH discussed the project. All authors read and approved the final manuscript.

Acknowledgments

References

1. Nigel Chaffey. Alberts, b., johnson, a., lewis, j., raff, m., roberts, k. and walter, p. molecular biology of the cell. 4th edn., 2003.
2. Kenneth Murphy and Casey Weaver. *Janeway's immunobiology*. Garland science, 2016.
3. Abul Abbas, Andrew Lichtman, and Shiv Pillai. *Cellular and molecular immunology E-book*. Elsevier Health Sciences, 2014.
4. Xiaoshan M Shao, Rohit Bhattacharya, Justin Huang, IK Ashok Sivakumar, Collin Tokheim, Lily Zheng, Dylan Hirsch, Benjamin Kaminow, Ashton Omdahl, Maria Bonsack, et al. High-throughput prediction of mhc class i and ii neoantigens with mhcnuggets. *Cancer immunology research*, 8(3):396–408, 2020.
5. Timothy J O'Donnell, Alex Rubinsteyn, and Uri Laserson. Mhcflurry 2.0: improved pan-allele prediction of mhc class i-presented peptides by incorporating antigen processing. *Cell systems*, 11(1):42–48, 2020.
6. Zhonghao Liu, Jing Jin, Yuxin Cui, Zheng Xiong, Alireza Nasiri, Yong Zhao, and Jianjun Hu. Deepseqpanii: an interpretable recurrent neural network model with attention mechanism for peptide-hla class ii binding prediction. *IEEE/ACM Transactions on Computational Biology and Bioinformatics*, 19(4):2188–2196, 2021.
7. Andrea T Nguyen, Christopher Szeto, and Stephanie Gras. The pockets guide to hla class i molecules. *Biochemical Society Transactions*, 49(5):2319–2331, 2021.
8. Ashish Vaswani, Noam Shazeer, Niki Parmar, Jakob Uszkoreit, Llion Jones, Aidan N Gomez, Łukasz Kaiser, and Illia Polosukhin. Attention is all you need. *Advances in neural information processing systems*, 30, 2017.
9. Kaiming He, Xiangyu Zhang, Shaoqing Ren, and Jian Sun. Deep residual learning for image recognition. In *Proceedings of the IEEE conference on computer vision and pattern recognition*, pages 770–778, 2016.
10. Zeming Lin, Halil Akin, Roshan Rao, Brian Hie, Zhongkai Zhu, Wenting Lu, Nikita Smetanin, Robert Verkuil, Ori Kabeli, Yaniv Shmueli, et al. Evolutionary-scale prediction of atomic-level protein structure with a language model. *Science*, 379(6637):1123–1130, 2023.
11. Yoon Kim. Convolutional neural networks for sentence classification. *arXiv preprint arXiv:1408.5882*, 2014.
12. Siwei Lai, Liheng Xu, Kang Liu, and Jun Zhao. Recurrent convolutional neural networks for text classification. In *Proceedings of the AAAI conference on artificial intelligence*, volume 29, 2015.
13. Rie Johnson and Tong Zhang. Deep pyramid convolutional neural networks for text categorization. In *Proceedings of the 55th Annual Meeting of the Association for Computational Linguistics (Volume 1: Long Papers)*, pages 562–570, 2017.
14. Peng Zhou, Wei Shi, Jun Tian, Zhenyu Qi, Bingchen Li, Hongwei Hao, and Bo Xu. Attention-based bidirectional long short-term memory networks for relation classification. In *Proceedings of the 54th annual meeting of the association for computational linguistics (volume 2: Short papers)*, pages 207–212, 2016.
15. Randi Vita, Swapnil Mahajan, James A Overton, Sandeep Kumar Dhanda, Sheridan Martini, Jason R Cantrell, Daniel K Wheeler, Alessandro Sette, and Bjoern Peters. The immune epitope database (iedb): 2018 update. *Nucleic acids research*, 47(D1):D339–D343, 2019.
16. Zeynep Koşaloğlu-Yalçın, Nina Blazeska, Randi Vita, Hannah Carter, Morten Nielsen, Stephen Schoenberger, Alessandro Sette, and Bjoern Peters. The cancer epitope database and analysis resource (cedar). *Nucleic Acids Research*, 51(D1):D845–D852, 2023.
17. Mikhail Shugay, Dmitriy V Bagaev, Ivan V Zvyagin, Renske M Vroomans, Jeremy Chase Crawford, Garry Dolton, Ekaterina A Komech, Anastasiya L Sycheva, Anna E Koneva, Evgeniy S Egorov, et al. Vdjdb: a curated database of t-cell receptor sequences with known antigen specificity. *Nucleic acids research*, 46(D1):D419–D427, 2018.
18. Sean Nolan, Marissa Vignali, Mark Klinger, Jennifer N Dines, Ian M Kaplan, Emily Svejnoha, Tracy Craft, Katie Boland, Mitch Pesesky, Rachel M Gittelman, et al. A large-scale database of t-cell receptor beta (tcrβ) sequences and binding associations from natural and synthetic exposure to sars-cov-2. *Research square*, 2020.
19. Manman Lu, Linfeng Xu, Xingxing Jian, Xiaoxiu Tan, Jingjing Zhao, Zhenhao Liu, Yu Zhang, Chunyu Liu, Lanming Chen, Yong Lin, et al. dbpepneo2. 0: A database for human tumor neoantigen peptides from mass spectrometry and tcr recognition. *Frontiers in Immunology*, 13:855976, 2022.
20. Jiaqi Xia, Peng Bai, Weiliang Fan, Qiming Li, Yongzheng Li, Dehe Wang, Lei Yin, and Yu Zhou. Nepdb: a database of

t-cell experimentally-validated neoantigens and pan-cancer predicted neoepitopes for cancer immunotherapy. *Frontiers in Immunology*, 12:644637, 2021.

21. Birkir Reynisson, Bruno Alvarez, Sinu Paul, Bjoern Peters, and Morten Nielsen. Netmhcpan-4.1 and netmhciipan-4.0: improved predictions of mhc antigen presentation by concurrent motif deconvolution and integration of ms mhc eluted ligand data. *Nucleic acids research*, 48(W1):W449–W454, 2020.
22. Benjamin Buchfink, Chao Xie, and Daniel H Huson. Fast and sensitive protein alignment using diamond. *Nature methods*, 12(1):59–60, 2015.
23. Kim D Pruitt, Tatiana Tatusova, and Donna R Maglott. Ncbi reference sequences (refseq): a curated non-redundant sequence database of genomes, transcripts and proteins. *Nucleic acids research*, 35(suppl_1):D61–D65, 2007.
24. Limin Fu, Beifang Niu, Zhengwei Zhu, Sitao Wu, and Weizhong Li. Cd-hit: accelerated for clustering the next-generation sequencing data. *Bioinformatics*, 28(23):3150–3152, 2012.
25. Bo Chen, Xingyi Cheng, Pan Li, Yangli-ao Geng, Jing Gong, Shen Li, Zhilei Bei, Xu Tan, Boyan Wang, Xin Zeng, et al. xtrimopglm: unified 100b-scale pre-trained transformer for deciphering the language of protein. *arXiv preprint arXiv:2401.06199*, 2024.
26. Ahmed Elnaggar, Michael Heinzinger, Christian Dallago, Ghalia Rehawi, Wang Yu, Llion Jones, Tom Gibbs, Tamas Feher, Christoph Angerer, Martin Steinegger, Debsindhu Bhowmik, and Burkhard Rost. Prottrans: Towards cracking the language of lifes code through self-supervised deep learning and high performance computing. *IEEE Transactions on Pattern Analysis and Machine Intelligence*, pages 1–1, 2021.
27. T.B. Brown, B. Mann, N. Ryder, M. Subbiah, J. Kaplan, P. Dhariwal, A. Neelakantan, P. Shyam, G. Sastry, A. Askell, S. Agarwal, A. Herbert-Voss, G. Krueger, T. Henighan, R. Child, A. Ramesh, D.M. Ziegler, J. Wu, C. Winter, C. Hesse, M. Chen, E. Sigler, M. Litwin, S. Gray, B. Chess, J. Clark, C. Berner, S. McCandlish, A. Radford, I. Sutskever, and D. Amodei. Language models are few-shot learners. *Advances in Neural Information Processing Systems*, 33:1877–1901, 2020.
28. Zhenjiao Du, Xingjian Ding, Yixiang Xu, and Yonghui Li. Unidl4biopep: a universal deep learning architecture for binary classification in peptide bioactivity. *Briefings in Bioinformatics*, 24(3):bbad135, 2023.
29. Ziyang Xu, Haitian Zhong, Bingrui He, Xueying Wang, and Tianchi Lu. Ptransips: Identification of phosphorylation sites enhanced by protein plm embeddings. *IEEE Journal of Biomedical and Health Informatics*, 2024.
30. John Jumper, Richard Evans, Alexander Pritzel, Tim Green, Michael Figurnov, Olaf Ronneberger, Kathryn Tunyasuvunakool, Russ Bates, Augustin Žídek, Anna Potapenko, et al. Highly accurate protein structure prediction with alphafold. *Nature*, 596(7873):583–589, 2021.
31. Milot Mirdita, Konstantin Schütze, Yoshitaka Moriwaki, Lim Heo, Sergey Ovchinnikov, and Martin Steinegger. Colabfold: making protein folding accessible to all. *Nature methods*, 19(6):679–682, 2022.
32. Jacob Devlin, Ming-Wei Chang, Kenton Lee, and Kristina Toutanova. Bert: Pre-training of deep bidirectional transformers for language understanding. *arXiv preprint arXiv:1810.04805*, 2018.
33. Malik Boudiaf, Ziko Imtiaz Masud, Jérôme Rony, José Dolz, Pablo Piantanida, and Ismail Ben Ayed. Transductive information maximization for few-shot learning, 2020.
34. Julien Racle, Justine Michaux, Georg Alexander Rockinger, Marion Arnaud, Sara Bobisse, Chloe Chong, Philippe Guillaume, George Coukos, Alexandre Harari, Camilla Jandus, et al. Robust prediction of hla class ii epitopes by deep motif deconvolution of immunopeptidomes. *Nature biotechnology*, 37(11):1283–1286, 2019.
35. Adi Nagler, Shelly Kalaora, Chaya Barbolin, Anastasia Gangaev, Steven LC Ketelaars, Michal Alon, Joy Pai, Gil Benedek, Yfat Yahalom-Ronen, Noam Erez, et al. Identification of presented sars-cov-2 hla class i and hla class ii peptides using hla peptidomics. *Cell Reports*, 35(13), 2021.
36. Yaqing Yang, Zhonghui Wei, Gabriel Cia, Xixi Song, Fabrizio Pucci, Marianne Rooman, Fuzhong Xue, and Qingzhen Hou. Mhcii-peptide presentation: an assessment of the state-of-the-art prediction methods. *Frontiers in Immunology*, 15:1293706, 2024.
37. Julien Racle, Philippe Guillaume, Julien Schmidt, Justine Michaux, Amédé Larabi, Kelvin Lau, Marta AS Perez, Giancarlo Croce, Raphaël Genolet, George Coukos, et al. Machine learning predictions of mhc-ii specificities reveal alternative binding mode of class ii epitopes. *Immunity*, 56(6):1359–1375, 2023.
38. Andrzej Maćkiewicz and Waldemar Ratajczak. Principal components analysis (pca). *Computers & Geosciences*, 19(3):303–342, 1993.
39. H-G Rammensee, Jutta Bachmann, Niels Philipp Nikolaus Emmerich, Oskar Alexander Bachor, and SSYFPEITHI Stevanović. Syfpeithi: database for mhc ligands and peptide motifs. *Immunogenetics*, 50:213–219, 1999.
40. Jonathan W Yewdell and Jack R Bennink. Mechanisms of viral interference with mhc class i antigen processing and presentation. *Annual review of cell and developmental biology*, 15(1):579–606, 1999.
41. Peter JA Cock, Tiago Antao, Jeffrey T Chang, Brad A Chapman, Cymon J Cox, Andrew Dalke, Iddo Friedberg, Thomas Hamelryck, Frank Kauff, Bartek Wilczynski, et al. Biopython: freely available python tools for computational molecular biology and bioinformatics. *Bioinformatics*, 25(11):1422, 2009.

Click here to access/download
Supplementary Material
supplementary file.pdf
