# Supplementary material for: TransHLA: a Hybrid Transformer model for HLA-presented epitope detection
Source: Gigascience. 2025 Feb 27;14:giaf008. doi: 10.1093/gigascience/giaf008 (PMC11878767; doi:10.1093/gigascience/giaf008)
Supplement: giaf008_GIGA-D-24-00176_Revision_3 [file giaf008_giga-d-24-00176_revision_3.pdf]

GigaScience

TransHLA: A Hybrid Transformer Model for HLA-Presented Epitope Detection

--Manuscript Draft--

**Manuscript Number:**	GIGA-D-24-00176R3
**Full Title:**	TransHLA: A Hybrid Transformer Model for HLA-Presented Epitope Detection
**Article Type:**	Technical Note
**Funding Information:**	An Immune Aging Monitor Based on the Dynamics of TCR Repertoire and Machine Learning (9080002) — Professor Shuaicheng LI
**Abstract:**	Background: Precise prediction of epitope presentation on human leukocyte antigen (HLA) molecules is crucial for advancing vaccine development and immunotherapy. Conventional HLA-peptide binding affinity prediction tools often focus on specific alleles and lack a universal approach for comprehensive HLA site analysis. This limitation hinders efficient filtering of invalid peptide segments.<br>Results: We introduce TransHLA, a pioneering tool designed for epitope prediction across all HLA alleles, integrating Transformer and Residue CNN architectures. TransHLA utilizes the ESM2 large language model for sequence and structure embeddings, achieving high predictive accuracy. For HLA class I, it reaches an accuracy of 84.72% and an AUC of 91.95% on IEDB test data. For HLA class II, it achieves 79.94% accuracy and an AUC of 88.14%. Our case studies using datasets like CEDAR and VDJdb demonstrate that TransHLA surpasses existing models in specificity and sensitivity for identifying immunogenic epitopes and neoepitopes.<br>Conclusions: TransHLA significantly enhances vaccine design and immunotherapy by efficiently identifying broadly reactive peptides. Our resources, including data and code, are publicly accessible at https://github.com/SkywalkerLuke/TransHLA
**Corresponding Author:**	Shuaicheng Li<br><br>HONG KONG
**Corresponding Author Secondary Information:**	
**Corresponding Author's Institution:**	
**Corresponding Author's Secondary Institution:**	
**First Author:**	Tianchi LU
**First Author Secondary Information:**	
**Order of Authors:**	Tianchi LU<br>Xueying Wang<br>Wan Nie<br>Miaozhe Huo<br>Shuaicheng LI
**Order of Authors Secondary Information:**	
**Response to Reviewers:**	Subject: Response to Editorial Office - GIGA-D-24-00176R2<br><br>Dear Dr. Zauner,<br><br>Thank you for your email regarding our manuscript "TransHLA: A Hybrid Transformer Model for HLA-Presented Epitope Detection" (GIGA-D-24-00176R2). We appreciate the feedback and the opportunity to revise our submission.<br><br>We are pleased to inform you that we have completed all the requests outlined in your letter:

Powered by Editorial Manager® and ProduXion Manager® from Aries Systems Corporation

	We have included the citation for the GigaDB dataset in the reference list and the data availability section.<br>The new software application has been registered in the bio.tools and SciCrunch.org databases, and the corresponding identifiers have been added to the manuscript.<br>RRIDs for other published software tools mentioned in the methods section have been included as requested.<br>URLs in the main text have been moved to the bibliography and cited appropriately.<br>All relevant accession numbers for data have been mentioned in the data availability section.<br>We have removed all highlighting and tracking of changes.<br>A reference to the DOME-ML annotations has been added to the bibliography, along with the appropriate citation in the data availability section.<br>Editable source files have been submitted, including LaTeX style and bib files.<br>We have ensured that the revised manuscript conforms to the journal style as per the Instructions for Authors.<br>We believe that these changes address all the requirements, and we look forward to your feedback on the revised manuscript.<br><br>Thank you once again for your guidance and support.<br><br>Best regards,<br><br>Shuaicheng Li
Additional Information:	
Question	Response
Are you submitting this manuscript to a special series or article collection?	No
**Experimental design and statistics**<br><br>Full details of the experimental design and statistical methods used should be given in the Methods section, as detailed in our Minimum Standards Reporting Checklist. Information essential to interpreting the data presented should be made available in the figure legends.<br><br>Have you included all the information requested in your manuscript?	Yes
**Resources**<br><br>A description of all resources used, including antibodies, cell lines, animals and software tools, with enough information to allow them to be uniquely identified, should be included in the Methods section. Authors are strongly encouraged to cite Research Resource Identifiers (RRIDs) for antibodies, model organisms and tools, where possible.	Yes

Have you included the information requested as detailed in our Minimum Standards Reporting Checklist?	
**Availability of data and materials**<br><br>All datasets and code on which the conclusions of the paper rely must be either included in your submission or deposited in publicly available repositories (where available and ethically appropriate), referencing such data using a unique identifier in the references and in the "Availability of Data and Materials" section of your manuscript.<br><br>Have you have met the above requirement as detailed in our Minimum Standards Reporting Checklist?	Yes

Powered by Editorial Manager® and ProduXion Manager® from Aries Systems Corporation

LaTeX - Main Document (TeX file)

Click here to access/download;LaTeX - Main Document (TeX file);main.tex

```
This is pdfTeX, Version 3.141592653-2.6-1.40.26 (TeX Live 2024)
(preloaded format=pdflatex 2024.8.2)  23 DEC 2024 02:23
entering extended mode
 restricted \write18 enabled.
 %&-line parsing enabled.
**main.tex
(./main.tex
LaTeX2e <2024-06-01> patch level 2
L3 programming layer <2024-05-27>
(./oup-contemporary.cls
Document Class: oup-contemporary 2023/06/12, v1.2
(c:/texlive/2024/texmf-dist/tex/latex/base/article.cls
Document Class: article 2024/02/08 v1.4n Standard LaTeX document class
(c:/texlive/2024/texmf-dist/tex/latex/base/size10.clo
File: size10.clo 2024/02/08 v1.4n Standard LaTeX file (size option)
)
\c@part=\count194
\c@section=\count195
\c@subsection=\count196
\c@subsubsection=\count197
\c@paragraph=\count198
\c@subparagraph=\count199
\c@figure=\count266
\c@table=\count267
\abovecaptionskip=\skip49
\belowcaptionskip=\skip50
\bibindent=\dimen141
) (c:/texlive/2024/texmf-dist/tex/latex/base/inputenc.sty
Package: inputenc 2024/02/08 v1.3d Input encoding file
\inpenc@prehook=\toks17
\inpenc@posthook=\toks18
) (c:/texlive/2024/texmf-dist/tex/latex/base/fontenc.sty
Package: fontenc 2021/04/29 v2.0v Standard LaTeX package
) (c:/texlive/2024/texmf-dist/tex/generic/iftex/ifpdf.sty
Package: ifpdf 2019/10/25 v3.4 ifpdf legacy package. Use iftex instead.
(c:/texlive/2024/texmf-dist/tex/generic/iftex/iftex.sty
Package: iftex 2022/02/03 v1.0f TeX engine tests
)) (c:/texlive/2024/texmf-dist/tex/latex/microtype/microtype.sty
Package: microtype 2024/03/29 v3.1b Micro-typographical refinements (RS)
(c:/texlive/2024/texmf-dist/tex/latex/graphics/keyval.sty
Package: keyval 2022/05/29 v1.15 key=value parser (DPC)
\KV@toks@=\toks19
) (c:/texlive/2024/texmf-dist/tex/latex/etoolbox/etoolbox.sty
Package: etoolbox 2020/10/05 v2.5k e-TeX tools for LaTeX (JAW)
\etb@tempcnta=\count268
)
\MT@toks=\toks20
\MT@tempbox=\box52
\MT@count=\count269
LaTeX Info: Redefining \noprotrusionifhmode on input line 1061.
LaTeX Info: Redefining \leftprotrusion on input line 1062.
\MT@prot@toks=\toks21
LaTeX Info: Redefining \rightprotrusion on input line 1081.
LaTeX Info: Redefining \textls on input line 1392.
```

```
\MT@outer@kern=\dimen142
LaTeX Info: Redefining \textmicrotypecontext on input line 2013.
\MT@listname@count=\count270
(c:/texlive/2024/texmf-dist/tex/latex/microtype/microtype-pdftex.def
File: microtype-pdftex.def 2024/03/29 v3.1b Definitions specific to
pdftex (RS)

LaTeX Info: Redefining \lsstyle on input line 902.
LaTeX Info: Redefining \lslig on input line 902.
\MT@outer@space=\skip51
)
Package microtype Info: Loading configuration file microtype.cfg.
(c:/texlive/2024/texmf-dist/tex/latex/microtype/microtype.cfg
File: microtype.cfg 2024/03/29 v3.1b microtype main configuration file
(RS)
)) (c:/texlive/2024/texmf-dist/tex/latex/euler/euler.sty
Package: euler 1995/03/05 v2.5
Package: `euler' v2.5 <1995/03/05> (FJ and FMi)
LaTeX Font Info:    Redeclaring symbol font `letters' on input line 35.
LaTeX Font Info:    Encoding `OML' has changed to `U' for symbol font
(Font)              `letters' in the math version `normal' on input line
35.
LaTeX Font Info:    Overwriting symbol font `letters' in version `normal'
(Font)                  OML/cmm/m/it --> U/eur/m/n on input line 35.
LaTeX Font Info:    Encoding `OML' has changed to `U' for symbol font
(Font)              `letters' in the math version `bold' on input line
35.
LaTeX Font Info:    Overwriting symbol font `letters' in version `bold'
(Font)                  OML/cmm/b/it --> U/eur/m/n on input line 35.
LaTeX Font Info:    Overwriting symbol font `letters' in version `bold'
(Font)                  U/eur/m/n --> U/eur/b/n on input line 36.
LaTeX Font Info:    Redeclaring math symbol \Gamma on input line 47.
LaTeX Font Info:    Redeclaring math symbol \Delta on input line 48.
LaTeX Font Info:    Redeclaring math symbol \Theta on input line 49.
LaTeX Font Info:    Redeclaring math symbol \Lambda on input line 50.
LaTeX Font Info:    Redeclaring math symbol \Xi on input line 51.
LaTeX Font Info:    Redeclaring math symbol \Pi on input line 52.
LaTeX Font Info:    Redeclaring math symbol \Sigma on input line 53.
LaTeX Font Info:    Redeclaring math symbol \Upsilon on input line 54.
LaTeX Font Info:    Redeclaring math symbol \Phi on input line 55.
LaTeX Font Info:    Redeclaring math symbol \Psi on input line 56.
LaTeX Font Info:    Redeclaring math symbol \Omega on input line 57.
\symEulerFraktur=\mathgroup4
LaTeX Font Info:    Overwriting symbol font `EulerFraktur' in version
`bold'
(Font)                  U/euf/m/n --> U/euf/b/n on input line 63.
LaTeX Info: Redefining \oldstylenums on input line 85.
\symEulerScript=\mathgroup5
LaTeX Font Info:    Overwriting symbol font `EulerScript' in version
`bold'
(Font)                  U/eus/m/n --> U/eus/b/n on input line 93.
LaTeX Font Info:    Redeclaring math symbol \aleph on input line 97.
LaTeX Font Info:    Redeclaring math symbol \Re on input line 98.
LaTeX Font Info:    Redeclaring math symbol \Im on input line 99.
```

LaTeX Font Info: Redeclaring math delimiter \vert on input line 101.
LaTeX Font Info: Redeclaring math delimiter \backslash on input line
103.
LaTeX Font Info: Redeclaring math symbol \neg on input line 106.
LaTeX Font Info: Redeclaring math symbol \wedge on input line 108.
LaTeX Font Info: Redeclaring math symbol \vee on input line 110.
LaTeX Font Info: Redeclaring math symbol \setminus on input line 112.
LaTeX Font Info: Redeclaring math symbol \sim on input line 113.
LaTeX Font Info: Redeclaring math symbol \mid on input line 114.
LaTeX Font Info: Redeclaring math delimiter \arrowvert on input line
116.
LaTeX Font Info: Redeclaring math symbol \mathsection on input line
117.
\symEulerExtension=\mathgroup6
LaTeX Font Info: Redeclaring math symbol \coprod on input line 125.
LaTeX Font Info: Redeclaring math symbol \prod on input line 125.
LaTeX Font Info: Redeclaring math symbol \sum on input line 125.
LaTeX Font Info: Redeclaring math symbol \intop on input line 130.
LaTeX Font Info: Redeclaring math symbol \ointop on input line 131.
LaTeX Font Info: Redeclaring math symbol \braceld on input line 132.
LaTeX Font Info: Redeclaring math symbol \bracerd on input line 133.
LaTeX Font Info: Redeclaring math symbol \bracelu on input line 134.
LaTeX Font Info: Redeclaring math symbol \braceru on input line 135.
LaTeX Font Info: Redeclaring math symbol \infty on input line 136.
LaTeX Font Info: Redeclaring math symbol \nearrow on input line 153.
LaTeX Font Info: Redeclaring math symbol \searrow on input line 154.
LaTeX Font Info: Redeclaring math symbol \nwarrow on input line 155.
LaTeX Font Info: Redeclaring math symbol \swarrow on input line 156.
LaTeX Font Info: Redeclaring math symbol \Leftrightarrow on input line
157.
LaTeX Font Info: Redeclaring math symbol \Leftarrow on input line 158.
LaTeX Font Info: Redeclaring math symbol \Rightarrow on input line
159.
LaTeX Font Info: Redeclaring math symbol \leftrightarrow on input line
160.
LaTeX Font Info: Redeclaring math symbol \leftarrow on input line 161.
LaTeX Font Info: Redeclaring math symbol \rightarrow on input line
163.
LaTeX Font Info: Redeclaring math delimiter \uparrow on input line
166.
LaTeX Font Info: Redeclaring math delimiter \downarrow on input line
168.
LaTeX Font Info: Redeclaring math delimiter \updownarrow on input line
170.
LaTeX Font Info: Redeclaring math delimiter \Uparrow on input line
172.
LaTeX Font Info: Redeclaring math delimiter \Downarrow on input line
174.
LaTeX Font Info: Redeclaring math delimiter \Updownarrow on input line
176.
LaTeX Font Info: Redeclaring math symbol \leftharpoonup on input line
177.
LaTeX Font Info: Redeclaring math symbol \leftharpoondown on input
line 178.

```
LaTeX Font Info:    Redeclaring math symbol \rightharpoonup on input line
179.
LaTeX Font Info:    Redeclaring math symbol \rightharpoondown on input
line 180
.
LaTeX Font Info:    Redeclaring math delimiter \lbrace on input line 182.
LaTeX Font Info:    Redeclaring math delimiter \rbrace on input line 184.
\symcmmigroup=\mathgroup7
LaTeX Font Info:    Overwriting symbol font `cmmigroup' in version `bold'
(Font)                  OML/cmm/m/it --> OML/cmm/b/it on input line 200.
LaTeX Font Info:    Redeclaring math accent \vec on input line 201.
LaTeX Font Info:    Redeclaring math symbol \triangleleft on input line
202.
LaTeX Font Info:    Redeclaring math symbol \triangleright on input line
203.
LaTeX Font Info:    Redeclaring math symbol \star on input line 204.
LaTeX Font Info:    Redeclaring math symbol \lhook on input line 205.
LaTeX Font Info:    Redeclaring math symbol \rhook on input line 206.
LaTeX Font Info:    Redeclaring math symbol \flat on input line 207.
LaTeX Font Info:    Redeclaring math symbol \natural on input line 208.
LaTeX Font Info:    Redeclaring math symbol \sharp on input line 209.
LaTeX Font Info:    Redeclaring math symbol \smile on input line 210.
LaTeX Font Info:    Redeclaring math symbol \frown on input line 211.
LaTeX Font Info:    Redeclaring math accent \grave on input line 245.
LaTeX Font Info:    Redeclaring math accent \acute on input line 246.
LaTeX Font Info:    Redeclaring math accent \tilde on input line 247.
LaTeX Font Info:    Redeclaring math accent \ddot on input line 248.
LaTeX Font Info:    Redeclaring math accent \check on input line 249.
LaTeX Font Info:    Redeclaring math accent \breve on input line 250.
LaTeX Font Info:    Redeclaring math accent \bar on input line 251.
LaTeX Font Info:    Redeclaring math accent \dot on input line 252.
LaTeX Font Info:    Redeclaring math accent \hat on input line 254.
) (c:/texlive/2024/texmf-dist/tex/latex/merriweather/merriweather.sty
Package: merriweather 2022/09/20 (Bob Tennent) Supports
Merriweather(Sans) font
s for all LaTeX engines.
(c:/texlive/2024/texmf-dist/tex/generic/iftex/ifxetex.sty
Package: ifxetex 2019/10/25 v0.7 ifxetex legacy package. Use iftex
instead.
) (c:/texlive/2024/texmf-dist/tex/generic/iftex/ifluatex.sty
Package: ifluatex 2019/10/25 v1.5 ifluatex legacy package. Use iftex
instead.
) (c:/texlive/2024/texmf-dist/tex/latex/base/textcomp.sty
Package: textcomp 2024/04/24 v2.1b Standard LaTeX package
) (c:/texlive/2024/texmf-dist/tex/latex/xkeyval/xkeyval.sty
Package: xkeyval 2022/06/16 v2.9 package option processing (HA)
(c:/texlive/2024/texmf-dist/tex/generic/xkeyval/xkeyval.tex
(c:/texlive/2024/te
xmf-dist/tex/generic/xkeyval/xkvutils.tex
\XKV@toks=\toks22
\XKV@tempa@toks=\toks23
)
\XKV@depth=\count271
```

```
File: xkeyval.tex 2014/12/03 v2.7a key=value parser (HA)
)) (c:/texlive/2024/texmf-dist/tex/latex/base/fontenc.sty
Package: fontenc 2021/04/29 v2.0v Standard LaTeX package
) (c:/texlive/2024/texmf-dist/tex/latex/fontaxes/fontaxes.sty
Package: fontaxes 2020/07/21 v1.0e Font selection axes
LaTeX Info: Redefining \upshape on input line 29.
LaTeX Info: Redefining \itshape on input line 31.
LaTeX Info: Redefining \slshape on input line 33.
LaTeX Info: Redefining \swshape on input line 35.
LaTeX Info: Redefining \scshape on input line 37.
LaTeX Info: Redefining \sscshape on input line 39.
LaTeX Info: Redefining \ulcshape on input line 41.
LaTeX Info: Redefining \textsw on input line 47.
LaTeX Info: Redefining \textssc on input line 48.
LaTeX Info: Redefining \textulc on input line 49.
)) (c:/texlive/2024/texmf-dist/tex/latex/mathastext/mathastext.sty
Package: mathastext 2024/07/27 v1.4b Use the text font in math mode (JFB)

Package mathastext Info: Starting the math mode configuration.
\mst@exists@muskip=\muskip17
\mst@forall@muskip=\muskip18
\mst@prime@muskip=\muskip19
\mst@do@nonletters=\toks24
\mst@undo@nonletters=\toks25
\mst@do@easynonletters=\toks26
\mst@undo@easynonletters=\toks27
\symmtoperatorfont=\mathgroup8
\symmtletterfont=\mathgroup9
(   mathastext:   ) ! and ?
(   mathastext:   ) punctuation: , . : ; and \colon
LaTeX Info: Redefining \relbar on input line 1201.
LaTeX Info: Redefining \rightarrowfill on input line 1202.
LaTeX Info: Redefining \leftarrowfill on input line 1205.
(   mathastext:   ) + and =
LaTeX Info: Redefining \Relbar on input line 1298.
(   mathastext:   ) adding = ; and + to \nfss@catcodes
(   mathastext:   ) parentheses ( ) [ ] and slash /
(   mathastext:   ) alldelims: < > \backslash \setminus | \vert \mid \{
\}
LaTeX Font Info:    Redeclaring math symbol \setminus on input line 1364.
LaTeX Info: Redefining \models on input line 1383.
(   mathastext:   ) \# \mathdollar \% \&
(   mathastext:   ) \imath and \jmath
LaTeX Font Info:    Overwriting math alphabet `\Mathnormalbold' in
version `nor
mal'
(Font)                  T1/Merriwthr-OsF/b/it --> T1/Merriwthr-OsF/b/it
on inpu
t line 2863.
LaTeX Font Info:    Overwriting math alphabet `\Mathnormalbold' in
version `bol
d'
(Font)                  T1/Merriwthr-OsF/b/it --> T1/Merriwthr-OsF/b/it
on inpu
```

```
t line 2863.
LaTeX Font Info:    Overwriting symbol font `mtletterfont' in version
`normal'
(Font)                  T1/Merriwthr-OsF/m/it --> T1/Merriwthr-OsF/m/it
on inpu
t line 2863.
LaTeX Font Info:    Overwriting symbol font `mtletterfont' in version
`bold'
(Font)                  T1/Merriwthr-OsF/m/it --> T1/Merriwthr-OsF/b/it
on inpu
t line 2863.
LaTeX Font Info:    Overwriting symbol font `mtoperatorfont' in version
`normal
'
(Font)                  T1/Merriwthr-OsF/m/n --> T1/Merriwthr-OsF/m/n on
input
line 2863.
LaTeX Font Info:    Overwriting symbol font `mtoperatorfont' in version
`bold'
(Font)                  T1/Merriwthr-OsF/m/n --> T1/Merriwthr-OsF/b/n on
input
line 2863.
LaTeX Font Info:    Overwriting math alphabet `\Mathbf' in version
`normal'
(Font)                  T1/Merriwthr-OsF/b/n --> T1/Merriwthr-OsF/b/n on
input
line 2863.
LaTeX Font Info:    Overwriting math alphabet `\Mathbf' in version `bold'
(Font)                  T1/Merriwthr-OsF/b/n --> T1/Merriwthr-OsF/b/n on
input
line 2863.
LaTeX Font Info:    Overwriting math alphabet `\Mathit' in version
`normal'
(Font)                  T1/Merriwthr-OsF/m/it --> T1/Merriwthr-OsF/m/it
on inpu
t line 2863.
LaTeX Font Info:    Overwriting math alphabet `\Mathit' in version `bold'
(Font)                  T1/Merriwthr-OsF/m/it --> T1/Merriwthr-OsF/b/it
on inpu
t line 2863.
LaTeX Font Info:    Overwriting math alphabet `\Mathsf' in version
`normal'
(Font)                  T1/MerriwthrSans-OsF/m/n --> T1/MerriwthrSans-
OsF/m/n o
n input line 2863.
LaTeX Font Info:    Overwriting math alphabet `\Mathsf' in version `bold'
(Font)                  T1/MerriwthrSans-OsF/m/n --> T1/MerriwthrSans-
OsF/b/n o
n input line 2863.
LaTeX Font Info:    Overwriting math alphabet `\Mathtt' in version
`normal'
(Font)                  T1/lmtt/m/n --> T1/lmtt/m/n on input line 2863.
LaTeX Font Info:    Overwriting math alphabet `\Mathtt' in version `bold'
(Font)                  T1/lmtt/m/n --> T1/lmtt/b/n on input line 2863.
```

```
(   mathastext:   ) Latin letters in the `normal', resp. `bold',
(   mathastext:   ) math versions are now set up to use the fonts
(   mathastext:   ) T1/Merriwthr-OsF/m/it, resp. T1/Merriwthr-OsF/b/it.
(   mathastext:   ) Other characters (digits, ...) and \log-like names
will be
(   mathastext:   ) typeset with the n shape.
(   mathastext:   ) \hbar
(   mathastext:   ) minus as endash
(   mathastext:   ) The italic option is in effect.
(   mathastext:   ) \HUGE has been (re)-defined.
(   mathastext:   ) mathastext has declared larger sizes for subscripts.
(   mathastext:   ) To keep LaTeX defaults, use option
`defaultmathsizes'.

Package mathastext Info: Loading is complete.  You can now use
\Mathastext to
(mathastext)             modify the normal and bold math versions.  Use
it
(mathastext)             with optional argument or use \MTDeclareVersion
to
(mathastext)             declare additional math versions.
) (c:/texlive/2024/texmf-dist/tex/latex/relsize/relsize.sty
Package: relsize 2013/03/29 ver 4.1
) (c:/texlive/2024/texmf-dist/tex/latex/ragged2e/ragged2e.sty
Package: ragged2e 2023/06/22 v3.6 ragged2e Package
\CenteringLeftskip=\skip52
\RaggedLeftLeftskip=\skip53
\RaggedRightLeftskip=\skip54
\CenteringRightskip=\skip55
\RaggedLeftRightskip=\skip56
\RaggedRightRightskip=\skip57
\CenteringParfillskip=\skip58
\RaggedLeftParfillskip=\skip59
\RaggedRightParfillskip=\skip60
\JustifyingParfillskip=\skip61
\CenteringParindent=\skip62
\RaggedLeftParindent=\skip63
\RaggedRightParindent=\skip64
\JustifyingParindent=\skip65
) (c:/texlive/2024/texmf-dist/tex/latex/xcolor/xcolor.sty
Package: xcolor 2023/11/15 v3.01 LaTeX color extensions (UK)
(c:/texlive/2024/texmf-dist/tex/latex/graphics-cfg/color.cfg
File: color.cfg 2016/01/02 v1.6 sample color configuration
)
Package xcolor Info: Driver file: pdftex.def on input line 274.
(c:/texlive/2024/texmf-dist/tex/latex/graphics-def/pdftex.def
File: pdftex.def 2024/04/13 v1.2c Graphics/color driver for pdftex
) (c:/texlive/2024/texmf-dist/tex/latex/graphics/mathcolor.ltx)
Package xcolor Info: Model `cmy' substituted by `cmy0' on input line
1350.
Package xcolor Info: Model `hsb' substituted by `rgb' on input line 1354.
Package xcolor Info: Model `RGB' extended on input line 1366.
Package xcolor Info: Model `HTML' substituted by `rgb' on input line
1368.
```

```
Package xcolor Info: Model `Hsb' substituted by `hsb' on input line 1369.
Package xcolor Info: Model `tHsb' substituted by `hsb' on input line
1370.
Package xcolor Info: Model `HSB' substituted by `hsb' on input line 1371.
Package xcolor Info: Model `Gray' substituted by `gray' on input line
1372.
Package xcolor Info: Model `wave' substituted by `hsb' on input line
1373.
) (c:/texlive/2024/texmf-dist/tex/latex/colortbl/colortbl.sty
Package: colortbl 2024/07/06 v1.0i Color table columns (DPC)
(c:/texlive/2024/texmf-dist/tex/latex/tools/array.sty
Package: array 2024/06/14 v2.6d Tabular extension package (FMi)
\col@sep=\dimen143
\ar@mcellbox=\box53
\extrarowheight=\dimen144
\NC@list=\toks28
\extratabsurround=\skip66
\backup@length=\skip67
\ar@cellbox=\box54
)
\everycr=\toks29
\minrowclearance=\skip68
\rownum=\count272
) (c:/texlive/2024/texmf-dist/tex/latex/graphics/graphicx.sty
Package: graphicx 2021/09/16 v1.2d Enhanced LaTeX Graphics (DPC,SPQR)
(c:/texlive/2024/texmf-dist/tex/latex/graphics/graphics.sty
Package: graphics 2024/05/23 v1.4g Standard LaTeX Graphics (DPC,SPQR)
(c:/texlive/2024/texmf-dist/tex/latex/graphics/trig.sty
Package: trig 2023/12/02 v1.11 sin cos tan (DPC)
) (c:/texlive/2024/texmf-dist/tex/latex/graphics-cfg/graphics.cfg
File: graphics.cfg 2016/06/04 v1.11 sample graphics configuration
)
Package graphics Info: Driver file: pdftex.def on input line 106.
)
\Gin@req@height=\dimen145
\Gin@req@width=\dimen146
) (c:/texlive/2024/texmf-dist/tex/latex/xpatch/xpatch.sty
(c:/texlive/2024/texm
f-dist/tex/latex/l3kernel/expl3.sty
Package: expl3 2024-05-27 L3 programming layer (loader)
(c:/texlive/2024/texmf-dist/tex/latex/l3backend/l3backend-pdftex.def
File: l3backend-pdftex.def 2024-05-08 L3 backend support: PDF output
(pdfTeX)
\l__color_backend_stack_int=\count273
\l__pdf_internal_box=\box55
))
Package: xpatch 2020/03/25 v0.3a Extending etoolbox patching commands
(c:/texlive/2024/texmf-dist/tex/latex/l3packages/xparse/xparse.sty
Package: xparse 2024-05-08 L3 Experimental document command parser
)) (c:/texlive/2024/texmf-dist/tex/latex/environ/environ.sty
Package: environ 2014/05/04 v0.3 A new way to define environments
(c:/texlive/2024/texmf-dist/tex/latex/trimspaces/trimspaces.sty
Package: trimspaces 2009/09/17 v1.1 Trim spaces around a token list
)
```

```
\@envbody=\toks30
) (c:/texlive/2024/texmf-dist/tex/latex/lastpage/lastpage.sty
Package: lastpage 2024/07/07 v2.1c lastpage: 2.09 or 2e? (HMM)
(c:/texlive/2024/texmf-dist/tex/latex/lastpage/lastpage2e.sty
Package: lastpage2e 2024/07/07 v2.1c Decide which 2e lastpage version to
use (H
MM)
(c:/texlive/2024/texmf-dist/tex/latex/lastpage/lastpagemodern.sty
Package: lastpagemodern 2024-07-07 v2.1c Refers to last page's name (HMM;
JPG)
\c@lastpagecount=\count274
)
)) (c:/texlive/2024/texmf-dist/tex/latex/graphics/rotating.sty
Package: rotating 2016/08/11 v2.16d rotated objects in LaTeX
(c:/texlive/2024/texmf-dist/tex/latex/base/ifthen.sty
Package: ifthen 2024/03/16 v1.1e Standard LaTeX ifthen package (DPC)
)
\c@r@tfl@t=\count275
\rotFPtop=\skip69
\rotFPbot=\skip70
\rot@float@box=\box56
\rot@mess@toks=\toks31
) (c:/texlive/2024/texmf-dist/tex/latex/graphics/lscape.sty
Package: lscape 2020/05/28 v3.02 Landscape Pages (DPC)
) (c:/texlive/2024/texmf-dist/tex/latex/tools/afterpage.sty
Package: afterpage 2023/07/04 v1.08 After-Page Package (DPC)
\AP@output=\toks32
\AP@partial=\box57
\AP@footins=\box58
) (c:/texlive/2024/texmf-dist/tex/latex/textpos/textpos.sty
Package: textpos 2022/07/23 v1.10.1
Package textpos Info: choosing support for LaTeX3 on input line 60.
\TP@textbox=\box59
\TP@holdbox=\box60
\TPHorizModule=\dimen147
\TPVertModule=\dimen148
\TP@margin=\dimen149
\TP@absmargin=\dimen150
Grid set 16 x 16 = 37.34424pt x 52.81541pt
\TPboxrulesize=\dimen151
\TP@ox=\dimen152
\TP@oy=\dimen153
\TP@tbargs=\toks33
TextBlockOrigin set to 0pt x 0pt
) (c:/texlive/2024/texmf-dist/tex/latex/url/url.sty
\Urlmuskip=\muskip20
Package: url 2013/09/16  ver 3.4  Verb mode for urls, etc.
) (c:/texlive/2024/texmf-dist/tex/latex/newfloat/newfloat.sty
Package: newfloat 2023/10/01 v1.2 Defining new floating environments (AR)
Package newfloat Info: `rotating' package detected.
) (c:/texlive/2024/texmf-dist/tex/latex/mdframed/mdframed.sty
Package: mdframed 2013/07/01 1.9b: mdframed
(c:/texlive/2024/texmf-dist/tex/latex/kvoptions/kvoptions.sty
```

```
Package: kvoptions 2022-06-15 v3.15 Key value format for package options
(HO)
(c:/texlive/2024/texmf-dist/tex/generic/ltxcmds/ltxcmds.sty
Package: ltxcmds 2023-12-04 v1.26 LaTeX kernel commands for general use
(HO)
) (c:/texlive/2024/texmf-dist/tex/latex/kvsetkeys/kvsetkeys.sty
Package: kvsetkeys 2022-10-05 v1.19 Key value parser (HO)
)) (c:/texlive/2024/texmf-dist/tex/latex/zref/zref-abspage.sty
Package: zref-abspage 2023-09-14 v2.35 Module abspage for zref (HO)
(c:/texlive/2024/texmf-dist/tex/latex/zref/zref-base.sty
Package: zref-base 2023-09-14 v2.35 Module base for zref (HO)
(c:/texlive/2024/texmf-dist/tex/generic/infwarerr/infwarerr.sty
Package: infwarerr 2019/12/03 v1.5 Providing info/warning/error messages
(HO)
) (c:/texlive/2024/texmf-dist/tex/generic/kvdefinekeys/kvdefinekeys.sty
Package: kvdefinekeys 2019-12-19 v1.6 Define keys (HO)
) (c:/texlive/2024/texmf-dist/tex/generic/pdftexcmds/pdftexcmds.sty
Package: pdftexcmds 2020-06-27 v0.33 Utility functions of pdfTeX for
LuaTeX (HO
)
Package pdftexcmds Info: \pdf@primitive is available.
Package pdftexcmds Info: \pdf@ifprimitive is available.
Package pdftexcmds Info: \pdfdraftmode found.
) (c:/texlive/2024/texmf-dist/tex/generic/etexcmds/etexcmds.sty
Package: etexcmds 2019/12/15 v1.7 Avoid name clashes with e-TeX commands
(HO)
) (c:/texlive/2024/texmf-dist/tex/latex/auxhook/auxhook.sty
Package: auxhook 2019-12-17 v1.6 Hooks for auxiliary files (HO)
)
Package zref Info: New property list: main on input line 767.
Package zref Info: New property: default on input line 768.
Package zref Info: New property: page on input line 769.
)
\c@abspage=\count276
Package zref Info: New property: abspage on input line 67.
) (c:/texlive/2024/texmf-dist/tex/latex/needspace/needspace.sty
Package: needspace 2010/09/12 v1.3d reserve vertical space
)
\mdf@templength=\skip71
\c@mdf@globalstyle@cnt=\count277
\mdf@skipabove@length=\skip72
\mdf@skipbelow@length=\skip73
\mdf@leftmargin@length=\skip74
\mdf@rightmargin@length=\skip75
\mdf@innerleftmargin@length=\skip76
\mdf@innerrightmargin@length=\skip77
\mdf@innertopmargin@length=\skip78
\mdf@innerbottommargin@length=\skip79
\mdf@splittopskip@length=\skip80
\mdf@splitbottomskip@length=\skip81
\mdf@outermargin@length=\skip82
\mdf@innermargin@length=\skip83
\mdf@linewidth@length=\skip84
\mdf@innerlinewidth@length=\skip85
```

```
\mdf@middlelinewidth@length=\skip86
\mdf@outerlinewidth@length=\skip87
\mdf@roundcorner@length=\skip88
\mdf@footenotedistance@length=\skip89
\mdf@userdefinedwidth@length=\skip90
\mdf@needspace@length=\skip91
\mdf@frametitleaboveskip@length=\skip92
\mdf@frametitlebelowskip@length=\skip93
\mdf@frametitlerulewidth@length=\skip94
\mdf@frametitleleftmargin@length=\skip95
\mdf@frametitlerightmargin@length=\skip96
\mdf@shadowsize@length=\skip97
\mdf@extratopheight@length=\skip98
\mdf@subtitleabovelinewidth@length=\skip99
\mdf@subtitlebelowlinewidth@length=\skip100
\mdf@subtitleaboveskip@length=\skip101
\mdf@subtitlebelowskip@length=\skip102
\mdf@subtitleinneraboveskip@length=\skip103
\mdf@subtitleinnerbelowskip@length=\skip104
\mdf@subsubtitleabovelinewidth@length=\skip105
\mdf@subsubtitlebelowlinewidth@length=\skip106
\mdf@subsubtitleaboveskip@length=\skip107
\mdf@subsubtitlebelowskip@length=\skip108
\mdf@subsubtitleinneraboveskip@length=\skip109
\mdf@subsubtitleinnerbelowskip@length=\skip110
(c:/texlive/2024/texmf-dist/tex/latex/mdframed/md-frame-0.mdf
File: md-frame-0.mdf 2013/07/01\ 1.9b: md-frame-0
)
\mdf@frametitlebox=\box61
\mdf@footnotebox=\box62
\mdf@splitbox@one=\box63
\mdf@splitbox@two=\box64
\mdf@splitbox@save=\box65
\mdfsplitboxwidth=\skip111
\mdfsplitboxtotalwidth=\skip112
\mdfsplitboxheight=\skip113
\mdfsplitboxdepth=\skip114
\mdfsplitboxtotalheight=\skip115
\mdfframetitleboxwidth=\skip116
\mdfframetitleboxtotalwidth=\skip117
\mdfframetitleboxheight=\skip118
\mdfframetitleboxdepth=\skip119
\mdfframetitleboxtotalheight=\skip120
\mdffootnoteboxwidth=\skip121
\mdffootnoteboxtotalwidth=\skip122
\mdffootnoteboxheight=\skip123
\mdffootnoteboxdepth=\skip124
\mdffootnoteboxtotalheight=\skip125
\mdftotallinewidth=\skip126
\mdfboundingboxwidth=\skip127
\mdfboundingboxtotalwidth=\skip128
\mdfboundingboxheight=\skip129
\mdfboundingboxdepth=\skip130
\mdfboundingboxtotalheight=\skip131
```

```
\mdf@freevspace@length=\skip132
\mdf@horizontalwidthofbox@length=\skip133
\mdf@verticalmarginwhole@length=\skip134
\mdf@horizontalspaceofbox=\skip135
\mdfsubtitleheight=\skip136
\mdfsubsubtitleheight=\skip137
\c@mdfcountframes=\count278

****** mdframed patching \endmdf@trivlist

****** -- success******

\mdf@envdepth=\count279
\c@mdf@env@i=\count280
\c@mdf@env@ii=\count281
\c@mdf@zref@counter=\count282
Package zref Info: New property: mdf@pagevalue on input line 895.
) (c:/texlive/2024/texmf-dist/tex/latex/titlesec/titlesec.sty
Package: titlesec 2023/10/27 v2.16 Sectioning titles
\ttl@box=\box66
\beforetitleunit=\skip138
\aftertitleunit=\skip139
\ttl@plus=\dimen154
\ttl@minus=\dimen155
\ttl@toksa=\toks34
\titlewidth=\dimen156
\titlewidthlast=\dimen157
\titlewidthfirst=\dimen158
) (c:/texlive/2024/texmf-dist/tex/latex/koma-script/scrextend.sty
Package: scrextend 2023/07/07 v3.41 KOMA-Script package (extend other
classes w
ith features of KOMA-Script classes)
(c:/texlive/2024/texmf-dist/tex/latex/koma-script/scrkbase.sty
Package: scrkbase 2023/07/07 v3.41 KOMA-Script package (KOMA-Script-
dependent b
asics and keyval usage)
(c:/texlive/2024/texmf-dist/tex/latex/koma-script/scrbase.sty
Package: scrbase 2023/07/07 v3.41 KOMA-Script package (KOMA-Script-
independent
basics and keyval usage)
(c:/texlive/2024/texmf-dist/tex/latex/koma-script/scrlfile.sty
Package: scrlfile 2023/07/07 v3.41 KOMA-Script package (file load hooks)
(c:/texlive/2024/texmf-dist/tex/latex/koma-script/scrlfile-hook.sty
Package: scrlfile-hook 2023/07/07 v3.41 KOMA-Script package (using LaTeX
hooks)

(c:/texlive/2024/texmf-dist/tex/latex/koma-script/scrlogo.sty
Package: scrlogo 2023/07/07 v3.41 KOMA-Script package (logo)
)))
Applying: [2021/05/01] Usage of raw or classic option list on input line
252.
Already applied: [0000/00/00] Usage of raw or classic option list on
input line
 368.
```

```
))
Package scrextend Info: unexpected definition of `\@makefnmark'.
(scrextend)             Trying to patch it on input line 1762.
Package scrextend Info: patch seems to be successfull on input line 1762.
)

LaTeX Font Warning: Font shape `T1/cmr/m/n' in size <7.5> not available
(Font)              size <7> substituted on input line 69.

(c:/texlive/2024/texmf-dist/tex/latex/tools/calc.sty
Package: calc 2023/07/08 v4.3 Infix arithmetic (KKT,FJ)
\calc@Acount=\count283
\calc@Bcount=\count284
\calc@Adimen=\dimen159
\calc@Bdimen=\dimen160
\calc@Askip=\skip140
\calc@Bskip=\skip141
LaTeX Info: Redefining \setlength on input line 80.
LaTeX Info: Redefining \addtolength on input line 81.
\calc@Ccount=\count285
\calc@Cskip=\skip142
) (c:/texlive/2024/texmf-dist/tex/latex/geometry/geometry.sty
Package: geometry 2020/01/02 v5.9 Page Geometry
(c:/texlive/2024/texmf-dist/tex/generic/iftex/ifvtex.sty
Package: ifvtex 2019/10/25 v1.7 ifvtex legacy package. Use iftex instead.
)
\Gm@cnth=\count286
\Gm@cntv=\count287
\c@Gm@tempcnt=\count288
\Gm@bindingoffset=\dimen161
\Gm@wd@mp=\dimen162
\Gm@odd@mp=\dimen163
\Gm@even@mp=\dimen164
\Gm@layoutwidth=\dimen165
\Gm@layoutheight=\dimen166
\Gm@layouthoffset=\dimen167
\Gm@layoutvoffset=\dimen168
\Gm@dimlist=\toks35
) (c:/texlive/2024/texmf-dist/tex/latex/preprint/authblk.sty
Package: authblk 2001/02/27 1.3 (PWD)
\affilsep=\skip143
\@affilsep=\skip144
\c@Maxaffil=\count289
\c@authors=\count290
\c@affil=\count291
) (c:/texlive/2024/texmf-dist/tex/latex/footmisc/footmisc.sty
Package: footmisc 2023/07/05 v6.0f a miscellany of footnote facilities
\FN@temptoken=\toks36
\footnotemargin=\dimen169
\@outputbox@depth=\dimen170
Package footmisc Info: Declaring symbol style bringhurst on input line
696.
Package footmisc Info: Declaring symbol style chicago on input line 704.
Package footmisc Info: Declaring symbol style wiley on input line 713.
```

```
Package footmisc Info: Declaring symbol style lamport-robust on input
line 724.

Package footmisc Info: Declaring symbol style lamport* on input line 744.
Package footmisc Info: Declaring symbol style lamport*-robust on input
line 765
.
) (c:/texlive/2024/texmf-dist/tex/latex/fancyhdr/fancyhdr.sty
Package: fancyhdr 2024/07/23 v4.3.1 Extensive control of page headers and
foote
rs
\f@nch@headwidth=\skip145
\f@nch@O@elh=\skip146
\f@nch@O@erh=\skip147
\f@nch@O@olh=\skip148
\f@nch@O@orh=\skip149
\f@nch@O@elf=\skip150
\f@nch@O@erf=\skip151
\f@nch@O@olf=\skip152
\f@nch@O@orf=\skip153
) (c:/texlive/2024/texmf-dist/tex/generic/alphalph/alphalph.sty
Package: alphalph 2019/12/09 v2.6 Convert numbers to letters (HO)
(c:/texlive/2024/texmf-dist/tex/generic/intcalc/intcalc.sty
Package: intcalc 2019/12/15 v1.3 Expandable calculations with integers
(HO)
))
\c@authorfn=\count292
(c:/texlive/2024/texmf-dist/tex/latex/abstract/abstract.sty
Package: abstract 2009/06/08 v1.2a configurable abstracts
\abstitleskip=\skip154
\absleftindent=\skip155
\absrightindent=\skip156
\absparindent=\skip157
\absparsep=\skip158
)
Package newfloat Info: New float `keypoints' with options
`placement=t!,name=kp
t' on input line 291.
\c@keypoints=\count293
\newfloat@ftype=\count294
Package newfloat Info: float type `keypoints'=8 on input line 291.
(c:/texlive/2024/texmf-dist/tex/latex/enumitem/enumitem.sty
Package: enumitem 2019/06/20 v3.9 Customized lists
\labelindent=\skip159
\enit@outerparindent=\dimen171
\enit@toks=\toks37
\enit@inbox=\box67
\enit@count@id=\count295
\enitdp@description=\count296
) (c:/texlive/2024/texmf-dist/tex/latex/quoting/quoting.sty
Package: quoting 2014/01/28 v0.1c Consolidated environment for displayed
text
\quo@toppartop=\skip160
) (c:/texlive/2024/texmf-dist/tex/latex/sttools/stfloats.sty
```

```
Package: stfloats 2017/03/27 v3.3 Improve float mechanism and
baselineskip sett
ings
\@dblbotnum=\count297
\c@dblbotnumber=\count298
) (c:/texlive/2024/texmf-dist/tex/latex/booktabs/booktabs.sty
Package: booktabs 2020/01/12 v1.61803398 Publication quality tables
\heavyrulewidth=\dimen172
\lightrulewidth=\dimen173
\cmidrulewidth=\dimen174
\belowrulesep=\dimen175
\belowbottomsep=\dimen176
\aboverulesep=\dimen177
\abovetopsep=\dimen178
\cmidrulesep=\dimen179
\cmidrulekern=\dimen180
\defaultaddspace=\dimen181
\@cmidla=\count299
\@cmidlb=\count300
\@aboverulesep=\dimen182
\@belowrulesep=\dimen183
\@thisruleclass=\count301
\@lastruleclass=\count302
\@thisrulewidth=\dimen184
) (c:/texlive/2024/texmf-dist/tex/latex/tools/tabularx.sty
Package: tabularx 2023/12/11 v2.12a `tabularx' package (DPC)
\TX@col@width=\dimen185
\TX@old@table=\dimen186
\TX@old@col=\dimen187
\TX@target=\dimen188
\TX@delta=\dimen189
\TX@cols=\count303
\TX@ftn=\toks38
)
\enitdp@tablenotes=\count304
(c:/texlive/2024/texmf-dist/tex/latex/caption/caption.sty
Package: caption 2023/08/05 v3.6o Customizing captions (AR)
(c:/texlive/2024/texmf-dist/tex/latex/caption/caption3.sty
Package: caption3 2023/07/31 v2.4d caption3 kernel (AR)
\caption@tempdima=\dimen190
\captionmargin=\dimen191
\caption@leftmargin=\dimen192
\caption@rightmargin=\dimen193
\caption@width=\dimen194
\caption@indent=\dimen195
\caption@parindent=\dimen196
\caption@hangindent=\dimen197
Package caption Info: Standard document class detected.
)
\c@caption@flags=\count305
\c@continuedfloat=\count306
Package caption Info: rotating package is loaded.
Package caption Info: scrextend package is loaded.
\caption@addmargin@hsize=\dimen198
```

```
\caption@addmargin@linewidth=\dimen199
) (c:/texlive/2024/texmf-dist/tex/latex/natbib/natbib.sty
Package: natbib 2010/09/13 8.31b (PWD, AO)
\bibhang=\skip161
\bibsep=\skip162
LaTeX Info: Redefining \cite on input line 694.
\c@NAT@ctr=\count307
)) (c:/texlive/2024/texmf-dist/tex/latex/siunitx/siunitx.sty
Package: siunitx 2024-06-24 v3.3.19 A comprehensive (SI) units package
\l__siunitx_number_uncert_offset_int=\count308
\l__siunitx_number_exponent_fixed_int=\count309
\l__siunitx_number_min_decimal_int=\count310
\l__siunitx_number_min_integer_int=\count311
\l__siunitx_number_round_precision_int=\count312
\l__siunitx_number_lower_threshold_int=\count313
\l__siunitx_number_upper_threshold_int=\count314
\l__siunitx_number_group_first_int=\count315
\l__siunitx_number_group_size_int=\count316
\l__siunitx_number_group_minimum_int=\count317
\l__siunitx_angle_tmp_dim=\dimen256
\l__siunitx_angle_marker_box=\box68
\l__siunitx_angle_unit_box=\box69
\l__siunitx_compound_count_int=\count318
(c:/texlive/2024/texmf-dist/tex/latex/translations/translations.sty
Package: translations 2022/02/05 v1.12 internationalization of LaTeX2e
packages
 (CN)
) (c:/texlive/2024/texmf-dist/tex/latex/amsmath/amstext.sty
Package: amstext 2021/08/26 v2.01 AMS text
(c:/texlive/2024/texmf-dist/tex/latex/amsmath/amsgen.sty
File: amsgen.sty 1999/11/30 v2.0 generic functions
\@emptytoks=\toks39
\ex@=\dimen257
))
\l__siunitx_table_tmp_box=\box70
\l__siunitx_table_tmp_dim=\dimen258
\l__siunitx_table_column_width_dim=\dimen259
\l__siunitx_table_integer_box=\box71
\l__siunitx_table_decimal_box=\box72
\l__siunitx_table_uncert_box=\box73
\l__siunitx_table_before_box=\box74
\l__siunitx_table_after_box=\box75
\l__siunitx_table_before_dim=\dimen260
\l__siunitx_table_carry_dim=\dimen261
\l__siunitx_unit_tmp_int=\count319
\l__siunitx_unit_position_int=\count320
\l__siunitx_unit_total_int=\count321
) (c:/texlive/2024/texmf-dist/tex/latex/amsmath/amsmath.sty
Package: amsmath 2024/05/23 v2.17q AMS math features
\@mathmargin=\skip163
For additional information on amsmath, use the `?' option.
(c:/texlive/2024/texmf-dist/tex/latex/amsmath/amsbsy.sty
Package: amsbsy 1999/11/29 v1.2d Bold Symbols
\pmbraise@=\dimen262
```

```
) (c:/texlive/2024/texmf-dist/tex/latex/amsmath/amsopn.sty
Package: amsopn 2022/04/08 v2.04 operator names
)
\inf@bad=\count322
LaTeX Info: Redefining \frac on input line 233.
\uproot@=\count323
\leftroot@=\count324
LaTeX Info: Redefining \overline on input line 398.
LaTeX Info: Redefining \colon on input line 409.
\classnum@=\count325
\DOTSCASE@=\count326
LaTeX Info: Redefining \ldots on input line 495.
LaTeX Info: Redefining \dots on input line 498.
LaTeX Info: Redefining \cdots on input line 619.
\Mathstrutbox@=\box76
\strutbox@=\box77
LaTeX Info: Redefining \big on input line 721.
LaTeX Info: Redefining \Big on input line 722.
LaTeX Info: Redefining \bigg on input line 723.
LaTeX Info: Redefining \Bigg on input line 724.
\big@size=\dimen263
LaTeX Font Info:    Redeclaring font encoding OML on input line 742.
LaTeX Font Info:    Redeclaring font encoding OMS on input line 743.
\macc@depth=\count327
LaTeX Info: Redefining \bmod on input line 904.
LaTeX Info: Redefining \pmod on input line 909.
LaTeX Info: Redefining \smash on input line 939.
LaTeX Info: Redefining \relbar on input line 969.
LaTeX Info: Redefining \Relbar on input line 970.
\c@MaxMatrixCols=\count328
\dotsspace@=\muskip21
\c@parentequation=\count329
\dspbrk@lvl=\count330
\tag@help=\toks40
\row@=\count331
\column@=\count332
\maxfields@=\count333
\andhelp@=\toks41
\eqnshift@=\dimen264
\alignsep@=\dimen265
\tagshift@=\dimen266
\tagwidth@=\dimen267
\totwidth@=\dimen268
\lineht@=\dimen269
\@envbody=\toks42
\multlinegap=\skip164
\multlinetaggap=\skip165
\mathdisplay@stack=\toks43
LaTeX Info: Redefining \[ on input line 2953.
LaTeX Info: Redefining \] on input line 2954.
) (c:/texlive/2024/texmf-dist/tex/latex/lineno/lineno.sty
Package: lineno 2023/05/20 line numbers on paragraphs v5.3
\linenopenalty=\count334
\output=\toks44
```

```
\linenoprevgraf=\count335
\linenumbersep=\dimen270
\linenumberwidth=\dimen271
\c@linenumber=\count336
\c@pagewiselinenumber=\count337
\c@LN@truepage=\count338
\c@internallinenumber=\count339
\c@internallinenumbers=\count340
\quotelinenumbersep=\dimen272
\bframerule=\dimen273
\bframesep=\dimen274
\bframebox=\box78
\linenoamsmath@ams@eqpen=\count341
LaTeX Info: Redefining \\ on input line 3180.
) (c:/texlive/2024/texmf-dist/tex/latex/orcidlink/orcidlink.sty
Package: orcidlink 2024/06/26 v1.1.0 Support ORCID's three different ID
formats
.
(c:/texlive/2024/texmf-dist/tex/latex/hyperref/hyperref.sty
Package: hyperref 2024-07-10 v7.01j Hypertext links for LaTeX
(c:/texlive/2024/texmf-dist/tex/generic/pdfescape/pdfescape.sty
Package: pdfescape 2019/12/09 v1.15 Implements pdfTeX's escape features
(HO)
) (c:/texlive/2024/texmf-dist/tex/latex/hycolor/hycolor.sty
Package: hycolor 2020-01-27 v1.10 Color options for hyperref/bookmark
(HO)
) (c:/texlive/2024/texmf-dist/tex/latex/hyperref/nameref.sty
Package: nameref 2023-11-26 v2.56 Cross-referencing by name of section
(c:/texlive/2024/texmf-dist/tex/latex/refcount/refcount.sty
Package: refcount 2019/12/15 v3.6 Data extraction from label references
(HO)
) (c:/texlive/2024/texmf-
dist/tex/generic/gettitlestring/gettitlestring.sty
Package: gettitlestring 2019/12/15 v1.6 Cleanup title references (HO)
)
\c@section@level=\count342
) (c:/texlive/2024/texmf-dist/tex/generic/stringenc/stringenc.sty
Package: stringenc 2019/11/29 v1.12 Convert strings between diff.
encodings (HO
)
)
\@linkdim=\dimen275
\Hy@linkcounter=\count343
\Hy@pagecounter=\count344
(c:/texlive/2024/texmf-dist/tex/latex/hyperref/pd1enc.def
File: pd1enc.def 2024-07-10 v7.01j Hyperref: PDFDocEncoding definition
(HO)
Now handling font encoding PD1 ...
... no UTF-8 mapping file for font encoding PD1
)
\Hy@SavedSpaceFactor=\count345
(c:/texlive/2024/texmf-dist/tex/latex/hyperref/puenc.def
File: puenc.def 2024-07-10 v7.01j Hyperref: PDF Unicode definition (HO)
Now handling font encoding PU ...
```

```
... no UTF-8 mapping file for font encoding PU
)
Package hyperref Info: Hyper figures OFF on input line 4157.
Package hyperref Info: Link nesting OFF on input line 4162.
Package hyperref Info: Hyper index ON on input line 4165.
Package hyperref Info: Plain pages OFF on input line 4172.
Package hyperref Info: Backreferencing OFF on input line 4177.
Package hyperref Info: Implicit mode ON; LaTeX internals redefined.
Package hyperref Info: Bookmarks ON on input line 4424.
\c@Hy@tempcnt=\count346
LaTeX Info: Redefining \url on input line 4763.
\XeTeXLinkMargin=\dimen276
(c:/texlive/2024/texmf-dist/tex/generic/bitset/bitset.sty
Package: bitset 2019/12/09 v1.3 Handle bit-vector datatype (HO)
(c:/texlive/2024/texmf-dist/tex/generic/bigintcalc/bigintcalc.sty
Package: bigintcalc 2019/12/15 v1.5 Expandable calculations on big
integers (HO
)
))
\Fld@menulength=\count347
\Field@Width=\dimen277
\Fld@charsize=\dimen278
Package hyperref Info: Hyper figures OFF on input line 6042.
Package hyperref Info: Link nesting OFF on input line 6047.
Package hyperref Info: Hyper index ON on input line 6050.
Package hyperref Info: backreferencing OFF on input line 6057.
Package hyperref Info: Link coloring OFF on input line 6062.
Package hyperref Info: Link coloring with OCG OFF on input line 6067.
Package hyperref Info: PDF/A mode OFF on input line 6072.
(c:/texlive/2024/texmf-dist/tex/latex/base/atbegshi-ltx.sty
Package: atbegshi-ltx 2021/01/10 v1.0c Emulation of the original atbegshi
package with kernel methods
)
\Hy@abspage=\count348
\c@Item=\count349
\c@Hfootnote=\count350
)
Package hyperref Info: Driver (autodetected): hpdftex.
(c:/texlive/2024/texmf-dist/tex/latex/hyperref/hpdftex.def
File: hpdftex.def 2024-07-10 v7.01j Hyperref driver for pdfTeX
(c:/texlive/2024/texmf-dist/tex/latex/base/atveryend-ltx.sty
Package: atveryend-ltx 2020/08/19 v1.0a Emulation of the original
atveryend pac
kage
with kernel methods
)
\HyAnn@Count=\count351
\Fld@listcount=\count352
\c@bookmark@seq@number=\count353
(c:/texlive/2024/texmf-dist/tex/latex/rerunfilecheck/rerunfilecheck.sty
Package: rerunfilecheck 2022-07-10 v1.10 Rerun checks for auxiliary files
(HO)
(c:/texlive/2024/texmf-dist/tex/generic/uniquecounter/uniquecounter.sty
```

```
Package: uniquecounter 2019/12/15 v1.4 Provide unlimited unique counter
(HO)
)
Package uniquecounter Info: New unique counter `rerunfilecheck' on input
line 2
85.
)
\Hy@SectionHShift=\skip166
) (c:/texlive/2024/texmf-dist/tex/latex/pgf/frontendlayer/tikz.sty
(c:/texlive/
2024/texmf-dist/tex/latex/pgf/basiclayer/pgf.sty (c:/texlive/2024/texmf-
dist/te
x/latex/pgf/utilities/pgfrcs.sty (c:/texlive/2024/texmf-
dist/tex/generic/pgf/ut
ilities/pgfutil-common.tex
\pgfutil@everybye=\toks45
\pgfutil@tempdima=\dimen279
\pgfutil@tempdimb=\dimen280
) (c:/texlive/2024/texmf-dist/tex/generic/pgf/utilities/pgfutil-latex.def
\pgfutil@abb=\box79
) (c:/texlive/2024/texmf-dist/tex/generic/pgf/utilities/pgfrcs.code.tex
(c:/tex
live/2024/texmf-dist/tex/generic/pgf/pgf.revision.tex)
Package: pgfrcs 2023-01-15 v3.1.10 (3.1.10)
))
Package: pgf 2023-01-15 v3.1.10 (3.1.10)
(c:/texlive/2024/texmf-dist/tex/latex/pgf/basiclayer/pgfcore.sty
(c:/texlive/20
24/texmf-dist/tex/latex/pgf/systemlayer/pgfsys.sty
(c:/texlive/2024/texmf-dist/
tex/generic/pgf/systemlayer/pgfsys.code.tex
Package: pgfsys 2023-01-15 v3.1.10 (3.1.10)
(c:/texlive/2024/texmf-dist/tex/generic/pgf/utilities/pgfkeys.code.tex
\pgfkeys@pathtoks=\toks46
\pgfkeys@temptoks=\toks47

(c:/texlive/2024/texmf-
dist/tex/generic/pgf/utilities/pgfkeyslibraryfiltered.co
de.tex
\pgfkeys@tmptoks=\toks48
))
\pgf@x=\dimen281
\pgf@y=\dimen282
\pgf@xa=\dimen283
\pgf@ya=\dimen284
\pgf@xb=\dimen285
\pgf@yb=\dimen286
\pgf@xc=\dimen287
\pgf@yc=\dimen288
\pgf@xd=\dimen289
\pgf@yd=\dimen290
\w@pgf@writea=\write3
\r@pgf@reada=\read2
\c@pgf@counta=\count354
```

```
\c@pgf@countb=\count355
\c@pgf@countc=\count356
\c@pgf@countd=\count357
\t@pgf@toka=\toks49
\t@pgf@tokb=\toks50
\t@pgf@tokc=\toks51
\pgf@sys@id@count=\count358
(c:/texlive/2024/texmf-dist/tex/generic/pgf/systemlayer/pgf.cfg
File: pgf.cfg 2023-01-15 v3.1.10 (3.1.10)
)
Driver file for pgf: pgfsys-pdftex.def
(c:/texlive/2024/texmf-dist/tex/generic/pgf/systemlayer/pgfsys-pdftex.def
File: pgfsys-pdftex.def 2023-01-15 v3.1.10 (3.1.10)
(c:/texlive/2024/texmf-dist/tex/generic/pgf/systemlayer/pgfsys-common-
pdf.def
File: pgfsys-common-pdf.def 2023-01-15 v3.1.10 (3.1.10)
)))
(c:/texlive/2024/texmf-
dist/tex/generic/pgf/systemlayer/pgfsyssoftpath.code.tex
File: pgfsyssoftpath.code.tex 2023-01-15 v3.1.10 (3.1.10)
\pgfsyssoftpath@smallbuffer@items=\count359
\pgfsyssoftpath@bigbuffer@items=\count360
)
(c:/texlive/2024/texmf-
dist/tex/generic/pgf/systemlayer/pgfsysprotocol.code.tex
File: pgfsysprotocol.code.tex 2023-01-15 v3.1.10 (3.1.10)
)) (c:/texlive/2024/texmf-
dist/tex/generic/pgf/basiclayer/pgfcore.code.tex
Package: pgfcore 2023-01-15 v3.1.10 (3.1.10)
(c:/texlive/2024/texmf-dist/tex/generic/pgf/math/pgfmath.code.tex
(c:/texlive/2
024/texmf-dist/tex/generic/pgf/math/pgfmathutil.code.tex)
(c:/texlive/2024/texm
f-dist/tex/generic/pgf/math/pgfmathparser.code.tex
\pgfmath@dimen=\dimen291
\pgfmath@count=\count361
\pgfmath@box=\box80
\pgfmath@toks=\toks52
\pgfmath@stack@operand=\toks53
\pgfmath@stack@operation=\toks54
) (c:/texlive/2024/texmf-
dist/tex/generic/pgf/math/pgfmathfunctions.code.tex)
(c:/texlive/2024/texmf-
dist/tex/generic/pgf/math/pgfmathfunctions.basic.code.te
x)
(c:/texlive/2024/texmf-
dist/tex/generic/pgf/math/pgfmathfunctions.trigonometric
.code.tex)
(c:/texlive/2024/texmf-
dist/tex/generic/pgf/math/pgfmathfunctions.random.code.t
ex)
(c:/texlive/2024/texmf-
dist/tex/generic/pgf/math/pgfmathfunctions.comparison.co
de.tex)
```

```
(c:/texlive/2024/texmf-
dist/tex/generic/pgf/math/pgfmathfunctions.base.code.tex
)
(c:/texlive/2024/texmf-
dist/tex/generic/pgf/math/pgfmathfunctions.round.code.te
x)
(c:/texlive/2024/texmf-
dist/tex/generic/pgf/math/pgfmathfunctions.misc.code.tex
)
(c:/texlive/2024/texmf-
dist/tex/generic/pgf/math/pgfmathfunctions.integerarithm
etics.code.tex) (c:/texlive/2024/texmf-
dist/tex/generic/pgf/math/pgfmathcalc.co
de.tex) (c:/texlive/2024/texmf-
dist/tex/generic/pgf/math/pgfmathfloat.code.tex
\c@pgfmathroundto@lastzeros=\count362
)) (c:/texlive/2024/texmf-dist/tex/generic/pgf/math/pgfint.code.tex)
(c:/texliv
e/2024/texmf-dist/tex/generic/pgf/basiclayer/pgfcorepoints.code.tex
File: pgfcorepoints.code.tex 2023-01-15 v3.1.10 (3.1.10)
\pgf@picminx=\dimen292
\pgf@picmaxx=\dimen293
\pgf@picminy=\dimen294
\pgf@picmaxy=\dimen295
\pgf@pathminx=\dimen296
\pgf@pathmaxx=\dimen297
\pgf@pathminy=\dimen298
\pgf@pathmaxy=\dimen299
\pgf@xx=\dimen300
\pgf@xy=\dimen301
\pgf@yx=\dimen302
\pgf@yy=\dimen303
\pgf@zx=\dimen304
\pgf@zy=\dimen305
)
(c:/texlive/2024/texmf-
dist/tex/generic/pgf/basiclayer/pgfcorepathconstruct.cod
e.tex
File: pgfcorepathconstruct.code.tex 2023-01-15 v3.1.10 (3.1.10)
\pgf@path@lastx=\dimen306
\pgf@path@lasty=\dimen307
)
(c:/texlive/2024/texmf-
dist/tex/generic/pgf/basiclayer/pgfcorepathusage.code.te
x
File: pgfcorepathusage.code.tex 2023-01-15 v3.1.10 (3.1.10)
\pgf@shorten@end@additional=\dimen308
\pgf@shorten@start@additional=\dimen309
) (c:/texlive/2024/texmf-
dist/tex/generic/pgf/basiclayer/pgfcorescopes.code.tex
File: pgfcorescopes.code.tex 2023-01-15 v3.1.10 (3.1.10)
\pgfpic=\box81
\pgf@hbox=\box82
\pgf@layerbox@main=\box83
```

```
\pgf@picture@serial@count=\count363
)
(c:/texlive/2024/texmf-
dist/tex/generic/pgf/basiclayer/pgfcoregraphicstate.code
.tex
File: pgfcoregraphicstate.code.tex 2023-01-15 v3.1.10 (3.1.10)
\pgflinewidth=\dimen310
)
(c:/texlive/2024/texmf-
dist/tex/generic/pgf/basiclayer/pgfcoretransformations.c
ode.tex
File: pgfcoretransformations.code.tex 2023-01-15 v3.1.10 (3.1.10)
\pgf@pt@x=\dimen311
\pgf@pt@y=\dimen312
\pgf@pt@temp=\dimen313
) (c:/texlive/2024/texmf-
dist/tex/generic/pgf/basiclayer/pgfcorequick.code.tex
File: pgfcorequick.code.tex 2023-01-15 v3.1.10 (3.1.10)
) (c:/texlive/2024/texmf-
dist/tex/generic/pgf/basiclayer/pgfcoreobjects.code.te
x
File: pgfcoreobjects.code.tex 2023-01-15 v3.1.10 (3.1.10)
)
(c:/texlive/2024/texmf-
dist/tex/generic/pgf/basiclayer/pgfcorepathprocessing.co
de.tex
File: pgfcorepathprocessing.code.tex 2023-01-15 v3.1.10 (3.1.10)
) (c:/texlive/2024/texmf-
dist/tex/generic/pgf/basiclayer/pgfcorearrows.code.tex
File: pgfcorearrows.code.tex 2023-01-15 v3.1.10 (3.1.10)
\pgfarrowsep=\dimen314
) (c:/texlive/2024/texmf-
dist/tex/generic/pgf/basiclayer/pgfcoreshade.code.tex
File: pgfcoreshade.code.tex 2023-01-15 v3.1.10 (3.1.10)
\pgf@max=\dimen315
\pgf@sys@shading@range@num=\count364
\pgf@shadingcount=\count365
) (c:/texlive/2024/texmf-
dist/tex/generic/pgf/basiclayer/pgfcoreimage.code.tex
File: pgfcoreimage.code.tex 2023-01-15 v3.1.10 (3.1.10)
)
(c:/texlive/2024/texmf-
dist/tex/generic/pgf/basiclayer/pgfcoreexternal.code.tex
File: pgfcoreexternal.code.tex 2023-01-15 v3.1.10 (3.1.10)
\pgfexternal@startupbox=\box84
) (c:/texlive/2024/texmf-
dist/tex/generic/pgf/basiclayer/pgfcorelayers.code.tex
File: pgfcorelayers.code.tex 2023-01-15 v3.1.10 (3.1.10)
)
(c:/texlive/2024/texmf-
dist/tex/generic/pgf/basiclayer/pgfcoretransparency.code
.tex
File: pgfcoretransparency.code.tex 2023-01-15 v3.1.10 (3.1.10)
)
```

```
(c:/texlive/2024/texmf-
dist/tex/generic/pgf/basiclayer/pgfcorepatterns.code.tex
File: pgfcorepatterns.code.tex 2023-01-15 v3.1.10 (3.1.10)
) (c:/texlive/2024/texmf-
dist/tex/generic/pgf/basiclayer/pgfcorerdf.code.tex
File: pgfcorerdf.code.tex 2023-01-15 v3.1.10 (3.1.10)
))) (c:/texlive/2024/texmf-
dist/tex/generic/pgf/modules/pgfmoduleshapes.code.te
x
File: pgfmoduleshapes.code.tex 2023-01-15 v3.1.10 (3.1.10)
\pgfnodeparttextbox=\box85
) (c:/texlive/2024/texmf-
dist/tex/generic/pgf/modules/pgfmoduleplot.code.tex
File: pgfmoduleplot.code.tex 2023-01-15 v3.1.10 (3.1.10)
)
(c:/texlive/2024/texmf-dist/tex/latex/pgf/compatibility/pgfcomp-version-
0-65.st
y
Package: pgfcomp-version-0-65 2023-01-15 v3.1.10 (3.1.10)
\pgf@nodesepstart=\dimen316
\pgf@nodesepend=\dimen317
)
(c:/texlive/2024/texmf-dist/tex/latex/pgf/compatibility/pgfcomp-version-
1-18.st
y
Package: pgfcomp-version-1-18 2023-01-15 v3.1.10 (3.1.10)
)) (c:/texlive/2024/texmf-dist/tex/latex/pgf/utilities/pgffor.sty
(c:/texlive/2
024/texmf-dist/tex/latex/pgf/utilities/pgfkeys.sty
(c:/texlive/2024/texmf-dist/
tex/generic/pgf/utilities/pgfkeys.code.tex)) (c:/texlive/2024/texmf-
dist/tex/la
tex/pgf/math/pgfmath.sty (c:/texlive/2024/texmf-
dist/tex/generic/pgf/math/pgfma
th.code.tex)) (c:/texlive/2024/texmf-
dist/tex/generic/pgf/utilities/pgffor.code
.tex
Package: pgffor 2023-01-15 v3.1.10 (3.1.10)
\pgffor@iter=\dimen318
\pgffor@skip=\dimen319
\pgffor@stack=\toks55
\pgffor@toks=\toks56
)) (c:/texlive/2024/texmf-
dist/tex/generic/pgf/frontendlayer/tikz/tikz.code.tex
Package: tikz 2023-01-15 v3.1.10 (3.1.10)

(c:/texlive/2024/texmf-
dist/tex/generic/pgf/libraries/pgflibraryplothandlers.co
de.tex
File: pgflibraryplothandlers.code.tex 2023-01-15 v3.1.10 (3.1.10)
\pgf@plot@mark@count=\count366
\pgfplotmarksize=\dimen320
)
\tikz@lastx=\dimen321
```

```
\tikz@lasty=\dimen322
\tikz@lastxsaved=\dimen323
\tikz@lastysaved=\dimen324
\tikz@lastmovetox=\dimen325
\tikz@lastmovetoy=\dimen326
\tikzleveldistance=\dimen327
\tikzsiblingdistance=\dimen328
\tikz@figbox=\box86
\tikz@figbox@bg=\box87
\tikz@tempbox=\box88
\tikz@tempbox@bg=\box89
\tikztreelevel=\count367
\tiknumberofchildren=\count368
\tiknumberofcurrentchild=\count369
\tikz@fig@count=\count370
(c:/texlive/2024/texmf-
dist/tex/generic/pgf/modules/pgfmodulematrix.code.tex
File: pgfmodulematrix.code.tex 2023-01-15 v3.1.10 (3.1.10)
\pgfmatrixcurrentrow=\count371
\pgfmatrixcurrentcolumn=\count372
\pgf@matrix@numberofcolumns=\count373
)
\tikz@expandcount=\count374

(c:/texlive/2024/texmf-
dist/tex/generic/pgf/frontendlayer/tikz/libraries/tikzli
brarytopaths.code.tex
File: tikzlibrarytopaths.code.tex 2023-01-15 v3.1.10 (3.1.10)
)))
(c:/texlive/2024/texmf-
dist/tex/generic/pgf/frontendlayer/tikz/libraries/tikzli
brarysvg.path.code.tex
File: tikzlibrarysvg.path.code.tex 2023-01-15 v3.1.10 (3.1.10)

(c:/texlive/2024/texmf-
dist/tex/generic/pgf/libraries/pgflibrarysvg.path.code.t
ex
File: pgflibrarysvg.path.code.tex 2023-01-15 v3.1.10 (3.1.10)
(c:/texlive/2024/texmf-
dist/tex/generic/pgf/modules/pgfmoduleparser.code.tex
File: pgfmoduleparser.code.tex 2023-01-15 v3.1.10 (3.1.10)
\pgfparserdef@arg@count=\count375
)
\pgf@lib@svg@last@x=\dimen329
\pgf@lib@svg@last@y=\dimen330
\pgf@lib@svg@last@c@x=\dimen331
\pgf@lib@svg@last@c@y=\dimen332
\pgf@lib@svg@count=\count376
\pgf@lib@svg@max@num=\count377
))
\@curXheight=\skip167
)

! LaTeX Error: Option clash for package hyperref.
```

```
See the LaTeX manual or LaTeX Companion for explanation.
Type  H <return>  for immediate help.
 ...

l.53 \begin{document}

The package hyperref has already been loaded with options:
  []
There has now been an attempt to load it with options
  [colorlinks,allcolors=black,urlcolor=blue]
Adding the global options:
  ,colorlinks,allcolors=black,urlcolor=blue
to your \documentclass declaration may fix this.
Try typing  <return>  to proceed.

Package translations Info: No language package found. I am going to use
`englis
h' as default language. on input line 53.
LaTeX Font Info:    Trying to load font information for T1+Merriwthr-OsF
on inp
ut line 53.
(c:/texlive/2024/texmf-dist/tex/latex/merriweather/T1Merriwthr-OsF.fd
File: T1Merriwthr-OsF.fd 2020/08/30 (autoinst) Font definitions for
T1/Merriwth
r-OsF.
)
LaTeX Font Info:    Font shape `T1/Merriwthr-OsF/m/n' will be
(Font)              scaled to size 7.5pt on input line 53.
(./main.aux)
\openout1 = `main.aux'.

LaTeX Font Info:    Checking defaults for OML/cmm/m/it on input line 53.
LaTeX Font Info:    ... okay on input line 53.
LaTeX Font Info:    Checking defaults for OMS/cmsy/m/n on input line 53.
LaTeX Font Info:    ... okay on input line 53.
LaTeX Font Info:    Checking defaults for OT1/cmr/m/n on input line 53.
LaTeX Font Info:    ... okay on input line 53.
LaTeX Font Info:    Checking defaults for T1/cmr/m/n on input line 53.
LaTeX Font Info:    ... okay on input line 53.
LaTeX Font Info:    Checking defaults for TS1/cmr/m/n on input line 53.
LaTeX Font Info:    ... okay on input line 53.
LaTeX Font Info:    Checking defaults for OMX/cmex/m/n on input line 53.
LaTeX Font Info:    ... okay on input line 53.
LaTeX Font Info:    Checking defaults for U/cmr/m/n on input line 53.
LaTeX Font Info:    ... okay on input line 53.
LaTeX Font Info:    Checking defaults for PD1/pdf/m/n on input line 53.
LaTeX Font Info:    ... okay on input line 53.
LaTeX Font Info:    Checking defaults for PU/pdf/m/n on input line 53.
LaTeX Font Info:    ... okay on input line 53.
LaTeX Info: Redefining \microtypecontext on input line 53.
Package microtype Info: Applying patch `item' on input line 53.
Package microtype Info: Applying patch `toc' on input line 53.
Package microtype Info: Applying patch `eqnum' on input line 53.
```

```
Package microtype Info: Applying patch `footnote' on input line 53.
Package microtype Info: Applying patch `verbatim' on input line 53.
Package microtype Info: Generating PDF output.
Package microtype Info: Character protrusion enabled (level 2).
Package microtype Info: Using default protrusion set `alltext'.
Package microtype Info: Automatic font expansion enabled (level 2),
(microtype)             stretch: 20, shrink: 20, step: 1, non-selected.
Package microtype Info: Using default expansion set `alltext-nott'.
LaTeX Info: Redefining \showhyphens on input line 53.
Package microtype Info: No adjustment of tracking.
Package microtype Info: No adjustment of interword spacing.
Package microtype Info: No adjustment of character kerning.
Package microtype Info: Loading generic protrusion settings for font
family
(microtype)             `Merriwthr-OsF' (encoding: T1).
(microtype)             For optimal results, create family-specific
settings.
(microtype)             See the microtype manual for details.
LaTeX Font Info:    Redeclaring symbol font `operators' on input line 53.
LaTeX Font Info:    Encoding `OT1' has changed to `T1' for symbol font
(Font)              `operators' in the math version `normal' on input
line 53.
LaTeX Font Info:    Overwriting symbol font `operators' in version
`normal'
(Font)                  OT1/cmr/m/n --> T1/Merriwthr-OsF/m/up on input
line 53.

LaTeX Font Info:    Encoding `OT1' has changed to `T1' for symbol font
(Font)              `operators' in the math version `bold' on input line
53.
LaTeX Font Info:    Overwriting symbol font `operators' in version `bold'
(Font)                  OT1/cmr/bx/n --> T1/Merriwthr-OsF/m/up on input
line 53
.
LaTeX Font Info:    Overwriting symbol font `operators' in version `bold'
(Font)                  T1/Merriwthr-OsF/m/up --> T1/Merriwthr-OsF/b/up
on inpu
t line 53.
LaTeX Font Info:    Redeclaring math alphabet \mathbf on input line 53.
LaTeX Font Info:    Overwriting math alphabet `\mathbf' in version
`normal'
(Font)                  OT1/cmr/bx/n --> T1/Merriwthr-OsF/b/up on input
line 53
.
LaTeX Font Info:    Overwriting math alphabet `\mathbf' in version `bold'
(Font)                  OT1/cmr/bx/n --> T1/Merriwthr-OsF/b/up on input
line 53
.
LaTeX Font Info:    Redeclaring math alphabet \mathsf on input line 53.
LaTeX Font Info:    Overwriting math alphabet `\mathsf' in version
`normal'
(Font)                  OT1/cmss/m/n --> T1/MerriwthrSans-OsF/m/up on
input lin
e 53.
```

```
LaTeX Font Info:    Overwriting math alphabet `\mathsf' in version `bold'
(Font)                  OT1/cmss/bx/n --> T1/MerriwthrSans-OsF/m/up on
input li
ne 53.
LaTeX Font Info:    Redeclaring math alphabet \mathit on input line 53.
LaTeX Font Info:    Overwriting math alphabet `\mathit' in version
`normal'
(Font)                  OT1/cmr/m/it --> T1/Merriwthr-OsF/m/it on input
line 53
.
LaTeX Font Info:    Overwriting math alphabet `\mathit' in version `bold'
(Font)                  OT1/cmr/bx/it --> T1/Merriwthr-OsF/m/it on input
line 5
3.
LaTeX Font Info:    Redeclaring math alphabet \mathtt on input line 53.
LaTeX Font Info:    Overwriting math alphabet `\mathtt' in version
`normal'
(Font)                  OT1/cmtt/m/n --> T1/lmtt/m/up on input line 53.
LaTeX Font Info:    Overwriting math alphabet `\mathtt' in version `bold'
(Font)                  OT1/cmtt/m/n --> T1/lmtt/m/up on input line 53.
LaTeX Font Info:    Overwriting math alphabet `\mathsf' in version `bold'
(Font)                  T1/MerriwthrSans-OsF/m/up --> T1/MerriwthrSans-
OsF/b/up
 on input line 53.
LaTeX Font Info:    Overwriting math alphabet `\mathit' in version `bold'
(Font)                  T1/Merriwthr-OsF/m/it --> T1/Merriwthr-OsF/b/it
on inpu
t line 53.
\c@mv@tabular=\count378
\c@mv@boldtabular=\count379
(c:/texlive/2024/texmf-dist/tex/context/base/mkii/supp-pdf.mkii
[Loading MPS to PDF converter (version 2006.09.02).]
\scratchcounter=\count380
\scratchdimen=\dimen333
\scratchbox=\box90
\nofMPsegments=\count381
\nofMParguments=\count382
\everyMPshowfont=\toks57
\MPscratchCnt=\count383
\MPscratchDim=\dimen334
\MPnumerator=\count384
\makeMPintoPDFobject=\count385
\everyMPtoPDFconversion=\toks58
) (c:/texlive/2024/texmf-dist/tex/latex/epstopdf-pkg/epstopdf-base.sty
Package: epstopdf-base 2020-01-24 v2.11 Base part for package epstopdf
Package epstopdf-base Info: Redefining graphics rule for `.eps' on input
line 4
85.
(c:/texlive/2024/texmf-dist/tex/latex/latexconfig/epstopdf-sys.cfg
File: epstopdf-sys.cfg 2010/07/13 v1.3 Configuration of (r)epstopdf for
TeX Liv
e
))
*geometry* driver: auto-detecting
```

```
*geometry* detected driver: pdftex
*geometry* verbose mode - [ preamble ] result:
* driver: pdftex
* paper: a4paper
* layout: <same size as paper>
* layoutoffset:(h,v)=(0.0pt,0.0pt)
* modes: includefoot twoside
* h-part:(L,W,R)=(54.64pt, 488.22787pt, 54.64pt)
* v-part:(T,H,B)=(66.0pt, 745.04684pt, 34.0pt)
* \paperwidth=597.50787pt
* \paperheight=845.04684pt
* \textwidth=488.22787pt
* \textheight=715.04684pt
* \oddsidemargin=-17.62999pt
* \evensidemargin=-17.62999pt
* \topmargin=-47.76999pt
* \headheight=17.5pt
* \headsep=24.0pt
* \topskip=10.0pt
* \footskip=30.0pt
* \marginparwidth=48.0pt
* \marginparsep=10.0pt
* \columnsep=18.0pt
* \skip\footins=22.0pt plus 2.0pt
* \hoffset=0.0pt
* \voffset=0.0pt
* \mag=1000
* \@twocolumntrue
* \@twosidetrue
* \@mparswitchtrue
* \@reversemarginfalse
* (1in=72.27pt=25.4mm, 1cm=28.453pt)

Package caption Info: Begin \AtBeginDocument code.
Package caption Info: hyperref package is loaded.
Package caption Info: End \AtBeginDocument code.

(c:/texlive/2024/texmf-dist/tex/latex/translations/translations-basic-
dictionar
y-english.trsl
File: translations-basic-dictionary-english.trsl (english translation
file `tra
nslations-basic-dictionary')
)
Package translations Info: loading dictionary `translations-basic-
dictionary' f
or `english'. on input line 53.
Package hyperref Info: Link coloring OFF on input line 53.
(./main.out) (./main.out)
\@outlinefile=\write4
\openout4 = `main.out'.

\@gscitedetails=\box91
\@gscitedetailsheight=\skip168
```

```
\@gsheadbox=\box92
\@gsheadboxheight=\skip169
LaTeX Font Info:    Font shape `T1/Merriwthr-OsF/b/n' will be
(Font)              scaled to size 6.5pt on input line 53.
LaTeX Font Info:    Calculating math sizes for size <7.5> on input line
53.

LaTeX Font Warning: Font shape `T1/Merriwthr-OsF/m/up' undefined
(Font)              using `T1/Merriwthr-OsF/m/n' instead on input line
53.

LaTeX Font Info:    Font shape `T1/Merriwthr-OsF/m/up' will be
(Font)              scaled to size 6.24973pt on input line 53.
LaTeX Font Info:    Font shape `T1/Merriwthr-OsF/m/up' will be
(Font)              scaled to size 5.24997pt on input line 53.
LaTeX Font Info:    Trying to load font information for U+eur on input
line 53.

(c:/texlive/2024/texmf-dist/tex/latex/amsfonts/ueur.fd
File: ueur.fd 2013/01/14 v3.01 Euler Roman
) (c:/texlive/2024/texmf-dist/tex/latex/microtype/mt-eur.cfg
File: mt-eur.cfg 2006/07/31 v1.1 microtype config. file: AMS Euler Roman
(RS)
)

LaTeX Font Warning: Font shape `OMS/cmsy/m/n' in size <7.5> not available
(Font)              size <7> substituted on input line 53.

LaTeX Font Info:    Trying to load font information for U+euf on input
line 53.

(c:/texlive/2024/texmf-dist/tex/latex/amsfonts/ueuf.fd
File: ueuf.fd 2013/01/14 v3.01 Euler Fraktur
) (c:/texlive/2024/texmf-dist/tex/latex/microtype/mt-euf.cfg
File: mt-euf.cfg 2006/07/03 v1.1 microtype config. file: AMS Euler
Fraktur (RS)

)
LaTeX Font Info:    Trying to load font information for U+eus on input
line 53.

(c:/texlive/2024/texmf-dist/tex/latex/amsfonts/ueus.fd
File: ueus.fd 2013/01/14 v3.01 Euler Script
) (c:/texlive/2024/texmf-dist/tex/latex/microtype/mt-eus.cfg
File: mt-eus.cfg 2006/07/28 v1.2 microtype config. file: AMS Euler Script
(RS)
)
LaTeX Font Info:    Trying to load font information for U+euex on input
line 53
.
(c:/texlive/2024/texmf-dist/tex/latex/amsfonts/ueuex.fd
File: ueuex.fd 2013/01/14 v3.01 Euler extra symbols
)
```

```
LaTeX Font Warning: Font shape `OML/cmm/m/it' in size <7.5> not available
(Font)              size <7> substituted on input line 53.

LaTeX Font Info:    Font shape `T1/Merriwthr-OsF/m/n' will be
(Font)              scaled to size 6.24973pt on input line 53.
LaTeX Font Info:    Font shape `T1/Merriwthr-OsF/m/n' will be
(Font)              scaled to size 5.24997pt on input line 53.
LaTeX Font Info:    Font shape `T1/Merriwthr-OsF/m/it' will be
(Font)              scaled to size 7.5pt on input line 53.
LaTeX Font Info:    Font shape `T1/Merriwthr-OsF/m/it' will be
(Font)              scaled to size 6.24973pt on input line 53.
LaTeX Font Info:    Font shape `T1/Merriwthr-OsF/m/it' will be
(Font)              scaled to size 5.24997pt on input line 53.
LaTeX Font Info:    Font shape `T1/Merriwthr-OsF/m/n' will be
(Font)              scaled to size 8.0pt on input line 53.
LaTeX Font Info:    Font shape `T1/Merriwthr-OsF/m/it' will be
(Font)              scaled to size 8.0pt on input line 53.
LaTeX Font Info:    Font shape `T1/Merriwthr-OsF/b/it' will be
(Font)              scaled to size 8.0pt on input line 53.
TextBlockOrigin set to 4pc+6.64pt x 4pc+6pt
<oup.pdf, id=104, 597.50829pt x 845.0471pt>
File: oup.pdf Graphic file (type pdf)
<use oup.pdf>
Package pdftex.def Info: oup.pdf  used on input line 68.
(pdftex.def)             Requested size: 41.03665pt x 58.038pt.
<gigascience-logo.pdf, id=105, 99.37125pt x 33.12375pt>
File: gigascience-logo.pdf Graphic file (type pdf)
<use gigascience-logo.pdf>
Package pdftex.def Info: gigascience-logo.pdf  used on input line 68.
(pdftex.def)             Requested size: 126.00902pt x 42.0pt.

Overfull \hbox (54.64pt too wide) in paragraph at lines 68--68
[][]
 []

LaTeX Font Info:    Font shape `T1/Merriwthr-OsF/m/n' will be
(Font)              scaled to size 14.0pt on input line 68.
LaTeX Font Info:    Font shape `T1/Merriwthr-OsF/m/n' will be
(Font)              scaled to size 8.99997pt on input line 68.
LaTeX Font Info:    Calculating math sizes for size <14> on input line
68.
LaTeX Font Info:    Font shape `T1/Merriwthr-OsF/m/up' will be
(Font)              scaled to size 14.0pt on input line 68.
LaTeX Font Info:    Font shape `T1/Merriwthr-OsF/m/up' will be
(Font)              scaled to size 11.66617pt on input line 68.
LaTeX Font Info:    Font shape `T1/Merriwthr-OsF/m/up' will be
(Font)              scaled to size 9.79996pt on input line 68.
LaTeX Font Info:    Font shape `T1/Merriwthr-OsF/m/n' will be
(Font)              scaled to size 11.66617pt on input line 68.
LaTeX Font Info:    Font shape `T1/Merriwthr-OsF/m/n' will be
(Font)              scaled to size 9.79996pt on input line 68.
LaTeX Font Info:    Font shape `T1/Merriwthr-OsF/m/it' will be
(Font)              scaled to size 14.0pt on input line 68.
LaTeX Font Info:    Font shape `T1/Merriwthr-OsF/m/it' will be
```

```
(Font)               scaled to size 11.66617pt on input line 68.
LaTeX Font Info:    Font shape `T1/Merriwthr-OsF/m/it' will be
(Font)               scaled to size 9.79996pt on input line 68.
LaTeX Font Info:    Font shape `T1/Merriwthr-OsF/b/n' will be
(Font)               scaled to size 18.0pt on input line 68.
LaTeX Font Info:    Font shape `T1/Merriwthr-OsF/m/n' will be
(Font)               scaled to size 13.0pt on input line 68.
LaTeX Font Info:    Calculating math sizes for size <13> on input line
68.
LaTeX Font Info:    Font shape `T1/Merriwthr-OsF/m/up' will be
(Font)               scaled to size 13.0pt on input line 68.
LaTeX Font Info:    Font shape `T1/Merriwthr-OsF/m/up' will be
(Font)               scaled to size 10.83287pt on input line 68.
LaTeX Font Info:    Font shape `T1/Merriwthr-OsF/m/up' will be
(Font)               scaled to size 9.09996pt on input line 68.

LaTeX Font Warning: Font shape `OMS/cmsy/m/n' in size <13> not available
(Font)               size <12> substituted on input line 68.


LaTeX Font Warning: Font shape `OMX/cmex/m/n' in size <13> not available
(Font)               size <12> substituted on input line 68.


LaTeX Font Warning: Font shape `OML/cmm/m/it' in size <13> not available
(Font)               size <12> substituted on input line 68.

LaTeX Font Info:    Font shape `T1/Merriwthr-OsF/m/n' will be
(Font)               scaled to size 10.83287pt on input line 68.
LaTeX Font Info:    Font shape `T1/Merriwthr-OsF/m/n' will be
(Font)               scaled to size 9.09996pt on input line 68.
LaTeX Font Info:    Font shape `T1/Merriwthr-OsF/m/it' will be
(Font)               scaled to size 13.0pt on input line 68.
LaTeX Font Info:    Font shape `T1/Merriwthr-OsF/m/it' will be
(Font)               scaled to size 10.83287pt on input line 68.
LaTeX Font Info:    Font shape `T1/Merriwthr-OsF/m/it' will be
(Font)               scaled to size 9.09996pt on input line 68.
LaTeX Font Info:    Trying to load font information for TS1+Merriwthr-OsF
on in
put line 68.
(c:/texlive/2024/texmf-dist/tex/latex/merriweather/TS1Merriwthr-OsF.fd
File: TS1Merriwthr-OsF.fd 2020/08/30 (autoinst) Font definitions for
TS1/Merriw
thr-OsF.
)
LaTeX Font Info:    Font shape `TS1/Merriwthr-OsF/m/n' will be
(Font)               scaled to size 10.83287pt on input line 68.
Package microtype Info: Loading generic protrusion settings for font
family
(microtype)             `Merriwthr-OsF' (encoding: TS1).
(microtype)             For optimal results, create family-specific
settings.
(microtype)             See the microtype manual for details.
LaTeX Font Info:    Font shape `T1/Merriwthr-OsF/m/n' will be
```

```
(Font)                scaled to size 9.0pt on input line 68.
LaTeX Font Info:    Font shape `T1/Merriwthr-OsF/m/up' will be
(Font)                scaled to size 9.0pt on input line 68.
LaTeX Font Info:    Font shape `T1/Merriwthr-OsF/m/up' will be
(Font)                scaled to size 7.0pt on input line 68.
LaTeX Font Info:    Font shape `T1/Merriwthr-OsF/m/up' will be
(Font)                scaled to size 5.0pt on input line 68.
LaTeX Font Info:    Font shape `T1/Merriwthr-OsF/m/n' will be
(Font)                scaled to size 7.0pt on input line 68.
LaTeX Font Info:    Font shape `T1/Merriwthr-OsF/m/n' will be
(Font)                scaled to size 5.0pt on input line 68.
LaTeX Font Info:    Font shape `T1/Merriwthr-OsF/m/it' will be
(Font)                scaled to size 9.0pt on input line 68.
LaTeX Font Info:    Font shape `T1/Merriwthr-OsF/m/it' will be
(Font)                scaled to size 7.0pt on input line 68.
LaTeX Font Info:    Font shape `T1/Merriwthr-OsF/m/it' will be
(Font)                scaled to size 5.0pt on input line 68.
LaTeX Font Info:    Font shape `T1/Merriwthr-OsF/m/n' will be
(Font)                scaled to size 6.5pt on input line 68.
LaTeX Font Info:    Calculating math sizes for size <6.5> on input line
68.
LaTeX Font Info:    Font shape `T1/Merriwthr-OsF/m/up' will be
(Font)                scaled to size 6.5pt on input line 68.
LaTeX Font Info:    Font shape `T1/Merriwthr-OsF/m/up' will be
(Font)                scaled to size 5.41643pt on input line 68.
LaTeX Font Info:    Font shape `T1/Merriwthr-OsF/m/up' will be
(Font)                scaled to size 4.54997pt on input line 68.

LaTeX Font Warning: Font shape `OMS/cmsy/m/n' in size <6.5> not available
(Font)                size <6> substituted on input line 68.


LaTeX Font Warning: Font shape `OMS/cmsy/m/n' in size <5.41643> not
available
(Font)                size <5> substituted on input line 68.


LaTeX Font Warning: Font shape `OMS/cmsy/m/n' in size <4.54997> not
available
(Font)                size <5> substituted on input line 68.


LaTeX Font Warning: Font shape `OML/cmm/m/it' in size <6.5> not available
(Font)                size <6> substituted on input line 68.


LaTeX Font Warning: Font shape `OML/cmm/m/it' in size <5.41643> not
available
(Font)                size <5> substituted on input line 68.


LaTeX Font Warning: Font shape `OML/cmm/m/it' in size <4.54997> not
available
(Font)                size <5> substituted on input line 68.
```

```
LaTeX Font Info:    Font shape `T1/Merriwthr-OsF/m/n' will be
(Font)              scaled to size 5.41643pt on input line 68.
LaTeX Font Info:    Font shape `T1/Merriwthr-OsF/m/n' will be
(Font)              scaled to size 4.54997pt on input line 68.
LaTeX Font Info:    Font shape `T1/Merriwthr-OsF/m/it' will be
(Font)              scaled to size 6.5pt on input line 68.
LaTeX Font Info:    Font shape `T1/Merriwthr-OsF/m/it' will be
(Font)              scaled to size 5.41643pt on input line 68.
LaTeX Font Info:    Font shape `T1/Merriwthr-OsF/m/it' will be
(Font)              scaled to size 4.54997pt on input line 68.
LaTeX Font Info:    Font shape `TS1/Merriwthr-OsF/m/n' will be
(Font)              scaled to size 5.41643pt on input line 68.

Overfull \hbox (54.64pt too wide) in paragraph at lines 68--68
[][][]
 []

LaTeX Font Info:    Font shape `T1/Merriwthr-OsF/b/n' will be
(Font)              scaled to size 10.0pt on input line 68.
LaTeX Font Info:    Font shape `T1/Merriwthr-OsF/b/n' will be
(Font)              scaled to size 8.0pt on input line 68.
LaTeX Font Info:    Trying to load font information for T1+lmtt on input
line 6
8.
(c:/texlive/2024/texmf-dist/tex/latex/lm/t1lmtt.fd
File: t1lmtt.fd 2015/05/01 v1.6.1 Font defs for Latin Modern
)
Package microtype Info: Loading generic protrusion settings for font
family
(microtype)             `lmtt' (encoding: T1).
(microtype)             For optimal results, create family-specific
settings.
(microtype)             See the microtype manual for details.
LaTeX Font Info:    Font shape `T1/Merriwthr-OsF/m/up' will be
(Font)              scaled to size 8.0pt on input line 68.
LaTeX Font Info:    Font shape `T1/Merriwthr-OsF/m/up' will be
(Font)              scaled to size 6.0pt on input line 68.
LaTeX Font Info:    Font shape `T1/Merriwthr-OsF/m/n' will be
(Font)              scaled to size 6.0pt on input line 68.
LaTeX Font Info:    Font shape `T1/Merriwthr-OsF/m/it' will be
(Font)              scaled to size 6.0pt on input line 68.

Overfull \hbox (54.64pt too wide) in paragraph at lines 68--68
[][][]
 []

Package mdframed Info: mdframed works in twoside mode on input line 70.
LaTeX Font Info:    Font shape `T1/Merriwthr-OsF/b/n' will be
(Font)              scaled to size 8.2pt on input line 70.
LaTeX Font Info:    Font shape `TS1/Merriwthr-OsF/m/n' will be
(Font)              scaled to size 7.5pt on input line 72.
Package mdframed Info: mdframed inside float
 mdframed uses option nobreak mdframed on input line 77.
```

```
Package mdframed Info: mdframed inside a box
 mdframed uses option nobreak mdframed on input line 77.
LaTeX Font Info:    Font shape `T1/Merriwthr-OsF/m/n' will be
(Font)              scaled to size 10.0pt on input line 83.
LaTeX Font Info:    Font shape `T1/Merriwthr-OsF/m/n' will be
(Font)              scaled to size 3.75pt on input line 83.
LaTeX Font Info:    Trying to load font information for T1+MerriwthrSans-
OsF on
 input line 83.
(c:/texlive/2024/texmf-dist/tex/latex/merriweather/T1MerriwthrSans-OsF.fd
File: T1MerriwthrSans-OsF.fd 2020/08/30 (autoinst) Font definitions for
T1/Merr
iwthrSans-OsF.
)
LaTeX Font Info:    Font shape `T1/MerriwthrSans-OsF/m/n' will be
(Font)              scaled to size 3.75pt on input line 83.
Package microtype Info: Loading generic protrusion settings for font
family
(microtype)             `MerriwthrSans-OsF' (encoding: T1).
(microtype)             For optimal results, create family-specific
settings.
(microtype)             See the microtype manual for details.
LaTeX Font Info:    Font shape `T1/Merriwthr-OsF/b/n' will be
(Font)              scaled to size 7.5pt on input line 84.

Package natbib Warning: Citation `chaffey2003alberts' on page 1 undefined
on in
put line 84.


Package natbib Warning: Citation `murphy2016janeway' on page 1 undefined
on inp
ut line 84.


Package natbib Warning: Citation `abbas2014cellular' on page 1 undefined
on inp
ut line 84.


Package natbib Warning: Citation `shao2020high' on page 1 undefined on
input li
ne 88.


Package natbib Warning: Citation `2020mhcflurry' on page 1 undefined on
input l
ine 88.


Package natbib Warning: Citation `liu2021deepseqpanii' on page 1
undefined on i
nput line 88.
```

```
Package natbib Warning: Citation `nguyen2021pockets' on page 1 undefined on inp
ut line 88.



LaTeX Warning: File `figures/main_figure.pdf' not found on input line 101.


! Package pdftex.def Error: File `figures/main_figure.pdf' not found: using dra
ft setting.

See the pdftex.def package documentation for explanation.
Type  H <return>  for immediate help.
 ...

l.101 ...dth=1\textwidth]{figures/main_figure.pdf}

Try typing  <return>  to proceed.
If that doesn't work, type  X <return>  to quit.

LaTeX Font Info:    Font shape `T1/Merriwthr-OsF/b/n' will be
(Font)              scaled to size 6.0pt on input line 102.

Package natbib Warning: Citation `shao2020high' on page 1 undefined on input li
ne 106.


Package natbib Warning: Citation `mei2021anthem' on page 1 undefined on input l
ine 106.


Package natbib Warning: Citation `2020mhcflurry' on page 1 undefined on input l
ine 106.


Package natbib Warning: Citation `reynisson2020netmhcpan' on page 1 undefined o
n input line 106.


Package natbib Warning: Citation `reynisson2020netmhcpan' on page 1 undefined o
n input line 106.
```

```
Package natbib Warning: Citation `liu2021deepseqpanii' on page 1
undefined on i
nput line 106.


Package natbib Warning: Citation `chu2022transformer' on page 1 undefined
on in
put line 106.


Package natbib Warning: Citation `tadros2024predicting' on page 1
undefined on
input line 106.


Package natbib Warning: Citation `racle2023machine' on page 1 undefined
on inpu
t line 106.



LaTeX Font Info:    Font shape `T1/Merriwthr-OsF/m/n' will be
(Font)              scaled to size 7.8pt on input line 108.
LaTeX Font Info:    Font shape `T1/Merriwthr-OsF/b/n' will be
(Font)              scaled to size 7.8pt on input line 108.
[1{c:/texlive/2024/texmf-
var/fonts/map/pdftex/updmap/pdftex.map}{c:/texlive/202
4/texmf-
dist/fonts/enc/dvips/merriweather/merriwthr_posqbl.enc}{c:/texlive/2024
/texmf-
dist/fonts/enc/dvips/merriweather/merriwthr_owzwzj.enc}{c:/texlive/2024/
texmf-dist/fonts/enc/dvips/lm/lm-ec.enc}


{c:/texlive/2024/texmf-
dist/fonts/enc/dvips/merriweather/merriwthr_ags7qn.enc}
<./oup.pdf> <./gigascience-logo.pdf>]
LaTeX Font Info:    Font shape `T1/Merriwthr-OsF/m/it' will be
(Font)              scaled to size 7.8pt on input line 108.
[2]

Package natbib Warning: Citation `vaswani2017attentionisallyouneed' on
page 3 u
ndefined on input line 113.


Package natbib Warning: Citation `he2016deep' on page 3 undefined on
input line
 113.


Package natbib Warning: Citation `lin2023evolutionary' on page 3
undefined on i
nput line 113.
```

```
Package natbib Warning: Citation `kim2014convolutional' on page 3
undefined on
input line 113.


Package natbib Warning: Citation `lai2015recurrent' on page 3 undefined
on inpu
t line 113.


Package natbib Warning: Citation `johnson2017deep' on page 3 undefined on
input
 line 113.


Package natbib Warning: Citation `zhou2016attention' on page 3 undefined
on inp
ut line 113.

LaTeX Font Info:    Font shape `T1/Merriwthr-OsF/b/n' will be
(Font)              scaled to size 8.5pt on input line 131.
LaTeX Font Info:    Font shape `T1/Merriwthr-OsF/m/n' will be
(Font)              scaled to size 8.5pt on input line 131.
LaTeX Font Info:    Font shape `T1/Merriwthr-OsF/b/sl' in size <7.5> not
availa
ble
(Font)              Font shape `T1/Merriwthr-OsF/b/it' tried instead on
input l
ine 133.
LaTeX Font Info:    Font shape `T1/Merriwthr-OsF/b/it' will be
(Font)              scaled to size 7.5pt on input line 133.

Package natbib Warning: Citation `vita2019immune' on page 3 undefined on
input
line 134.


Package natbib Warning: Citation `kocsalouglu2023cancer' on page 3
undefined on
 input line 134.


Package natbib Warning: Citation `shugay2018vdjdb' on page 3 undefined on
input
 line 134.


Package natbib Warning: Citation `nolan2020large' on page 3 undefined on
input
line 134.
```

```
Package natbib Warning: Citation `lu2022dbpepneo2' on page 3 undefined on input
 line 134.


Package natbib Warning: Citation `xia2021nepdb' on page 3 undefined on input li
ne 134.




Package natbib Warning: Citation `reynisson2020netmhcpan' on page 3 undefined o
n input line 152.


Package natbib Warning: Citation `reynisson2020netmhcpan' on page 3 undefined o
n input line 152.


Package natbib Warning: Citation `vita2019immune' on page 3 undefined on input
line 154.


Package natbib Warning: Citation `buchfink2015fast' on page 3 undefined on inpu
t line 154.


Package natbib Warning: Citation `pruitt2007ncbi' on page 3 undefined on input
line 154.


Package natbib Warning: Citation `fu2012cd' on page 3 undefined on input line 1
56.

LaTeX Font Info:    Font shape `T1/Merriwthr-OsF/b/n' will be
(Font)              scaled to size 7.0pt on input line 168.

Package natbib Warning: Citation `chen2024xtrimopglm' on page 3 undefined on in
put line 183.


Package natbib Warning: Citation `lin2023evolutionary' on page 3 undefined on i
nput line 183.
```

```
Package natbib Warning: Citation `ProtTrans' on page 3 undefined on input
line
183.


Package natbib Warning: Citation `Brown2020' on page 3 undefined on input
line
183.


Package natbib Warning: Citation `du2023unidl4biopep' on page 3 undefined
on in
put line 183.


Package natbib Warning: Citation `xu2024ptransips' on page 3 undefined on
input
 line 183.


Package natbib Warning: Citation `jumper2021highly' on page 3 undefined
on inpu
t line 183.


Package natbib Warning: Citation `mirdita2022colabfold' on page 3
undefined on
input line 183.


Package natbib Warning: Citation `lin2023evolutionary' on page 3
undefined on i
nput line 185.



[3]

Package natbib Warning: Citation `vaswani2017attentionisallyouneed' on
page 4 u
ndefined on input line 194.


Package natbib Warning: Citation `devlin2018bert' on page 4 undefined on
input
line 194.

LaTeX Font Info:    Font shape `T1/Merriwthr-OsF/m/up' will be
(Font)              scaled to size 7.5pt on input line 194.
LaTeX Font Info:    Font shape `T1/Merriwthr-OsF/b/n' will be
(Font)              scaled to size 6.24973pt on input line 194.
LaTeX Font Info:    Font shape `T1/Merriwthr-OsF/b/n' will be
(Font)              scaled to size 5.24997pt on input line 194.
! Undefined control sequence.
```

```
l.194 ... represented as a $\mathbf{E} \in \mathbb
                                                  {R}^{1280 \times
peptide\_...
The control sequence at the end of the top line
of your error message was never \def'ed. If you have
misspelled it (e.g., `\hobx'), type `I' and the correct
spelling (e.g., `I\hbox'). Otherwise just continue,
and I'll forget about whatever was undefined.


LaTeX Warning: File `figures/HLA_I_allele_frequency.pdf' not found on
input lin
e 199.


! Package pdftex.def Error: File `figures/HLA_I_allele_frequency.pdf' not
found
: using draft setting.

See the pdftex.def package documentation for explanation.
Type  H <return>  for immediate help.
 ...

l.199 ...idth]{figures/HLA_I_allele_frequency.pdf}

Try typing  <return>  to proceed.
If that doesn't work, type  X <return>  to quit.

! Missing $ inserted.
<inserted text>
               $
l.215

I've inserted a begin-math/end-math symbol since I think
you left one out. Proceed, with fingers crossed.

! Display math should end with $$.
<to be read again>
                   \tex_par:D
l.215

The `$' that I just saw supposedly matches a previous `$$'.
So I shall assume that you typed `$$' both times.

! Missing $ inserted.
<inserted text>
               $
l.216 \text{where} \; \text{head}_
                                  j = \text{Attention}(QW_{Q_j},
KW_{K_j}, V...
I've inserted a begin-math/end-math symbol since I think
you left one out. Proceed, with fingers crossed.

! You can't use `\eqno' in math mode.
```

```
\veqno ->\@kernel@eqno
                       \aftergroup \ignorespaces
l.217 \end{equation}

Sorry, but I'm not programmed to handle this case;
I'll just pretend that you didn't ask for it.
If you're in the wrong mode, you might be able to
return to the right one by typing `I}' or `I$' or `I\par'.

! Missing $ inserted.
<inserted text>
                $
l.217 \end{equation}

I've inserted something that you may have forgotten.
(See the <inserted text> above.)
With luck, this will get me unwedged. But if you
really didn't forget anything, try typing `2' now; then
my insertion and my current dilemma will both disappear.

! Undefined control sequence.
l.226 ... symmetric matrix $\mathbf{S} \in \mathbb
                                                  {R}^{peptide\_length
\time...
The control sequence at the end of the top line
of your error message was never \def'ed. If you have
misspelled it (e.g., `\hobx'), type `I' and the correct
spelling (e.g., `I\hbox'). Otherwise just continue,
and I'll forget about whatever was undefined.


Underfull \hbox (badness 10000) in paragraph at lines 231--232

 []

! Undefined control sequence.
l.233 where $\mathbf{x} \in \mathbb
                                   {R}^{M \times D}$ and $M$ is the
number o...
The control sequence at the end of the top line
of your error message was never \def'ed. If you have
misspelled it (e.g., `\hobx'), type `I' and the correct
spelling (e.g., `I\hbox'). Otherwise just continue,
and I'll forget about whatever was undefined.



! Undefined control sequence.
l.239 where $\mathbf{z}_1 \in \mathbb
                                     {R}^{M \times 256}$.
The control sequence at the end of the top line
of your error message was never \def'ed. If you have
misspelled it (e.g., `\hobx'), type `I' and the correct
spelling (e.g., `I\hbox'). Otherwise just continue,
```

```
and I'll forget about whatever was undefined.

! Undefined control sequence.
l.248 where $\mathbf{x}_1 \in \mathbb
                                     {R}^{M/2 \times 256}$.
The control sequence at the end of the top line
of your error message was never \def'ed. If you have
misspelled it (e.g., `\hobx'), type `I' and the correct
spelling (e.g., `I\hbox'). Otherwise just continue,
and I'll forget about whatever was undefined.

! Undefined control sequence.
l.254 where $\mathbf{z}_2 \in \mathbb
                                     {R}^{M/2 \times 256}$.
The control sequence at the end of the top line
of your error message was never \def'ed. If you have
misspelled it (e.g., `\hobx'), type `I' and the correct
spelling (e.g., `I\hbox'). Otherwise just continue,
and I'll forget about whatever was undefined.

! Undefined control sequence.
l.263 where $\mathbf{z}_{l+1} \in \mathbb
                                         {R}^{M/2^{l} \times 256}$.
The control sequence at the end of the top line
of your error message was never \def'ed. If you have
misspelled it (e.g., `\hobx'), type `I' and the correct
spelling (e.g., `I\hbox'). Otherwise just continue,
and I'll forget about whatever was undefined.


Package natbib Warning: Citation `boudiaf2020transductive' on page 4
undefined
on input line 279.


Underfull \vbox (badness 10000) has occurred while \output is active []



[4] [5]


Package natbib Warning: Citation `kim2014convolutional' on page 6
undefined on
input line 346.


Package natbib Warning: Citation `lai2015recurrent' on page 6 undefined
on inpu
t line 346.


Package natbib Warning: Citation `johnson2017deep' on page 6 undefined on
input
```

```
 line 346.


Package natbib Warning: Citation `zhou2016attention' on page 6 undefined
on inp
ut line 346.


Package natbib Warning: Citation `racle2019robust' on page 6 undefined on
input
 line 352.


Package natbib Warning: Citation `nagler2021identification' on page 6
undefined
 on input line 352.


Package natbib Warning: Citation `yang2024mhcii' on page 6 undefined on
input l
ine 352.


Package natbib Warning: Citation `racle2023machine' on page 6 undefined
on inpu
t line 352.


LaTeX Warning: File `figures/Compare_study.pdf' not found on input line
388.


! Package pdftex.def Error: File `figures/Compare_study.pdf' not found:
using d
raft setting.

See the pdftex.def package documentation for explanation.
Type  H <return>  for immediate help.
 ...

l.388 ...h=1\textwidth]{figures/Compare_study.pdf}

Try typing  <return>  to proceed.
If that doesn't work, type  X <return>  to quit.



[6] [7]

Package natbib Warning: Citation `2020mhcflurry' on page 8 undefined on
input l
ine 408.
```

Package natbib Warning: Citation `reynisson2020netmhcpan' on page 8
undefined o
n input line 408.

Package natbib Warning: Citation `reynisson2020netmhcpan' on page 8
undefined o
n input line 408.

Package natbib Warning: Citation `chu2022transformer' on page 8 undefined
on in
put line 408.

Package natbib Warning: Citation `mei2021anthem' on page 8 undefined on
input l
ine 408.

Package natbib Warning: Citation `tadros2024predicting' on page 8
undefined on
input line 408.

Package natbib Warning: Citation `liu2021deepseqpanii' on page 8
undefined on i
nput line 408.

Package natbib Warning: Citation `racle2023machine' on page 8 undefined
on inpu
t line 408.

Package natbib Warning: Citation `shao2020high' on page 8 undefined on
input li
ne 408.

Package natbib Warning: Citation `kocsalouglu2023cancer' on page 8
undefined on
 input line 408.

Package natbib Warning: Citation `shugay2018vdjdb' on page 8 undefined on
input
 line 408.

Package natbib Warning: Citation `nolan2020large' on page 8 undefined on
input
line 408.

```
Package natbib Warning: Citation `lu2022dbpepneo2' on page 8 undefined on
input
 line 408.


Overfull \hbox (4.64067pt too wide) in paragraph at lines 442--442
[]|\T1/Merriwthr-OsF/m/n/7 (-20) NetMHCpan4.1b|
 []


Overfull \hbox (9.9608pt too wide) in paragraph at lines 442--442
[]|\T1/Merriwthr-OsF/m/n/7 (-20) NetMHCIIpan4.3b|
 []




LaTeX Warning: File `figures/PCA_embedding.pdf' not found on input line
449.


! Package pdftex.def Error: File `figures/PCA_embedding.pdf' not found:
using d
raft setting.

See the pdftex.def package documentation for explanation.
Type  H <return>  for immediate help.
 ...

l.449 ....85\textwidth]{figures/PCA_embedding.pdf}

Try typing  <return>  to proceed.
If that doesn't work, type  X <return>  to quit.


Overfull \hbox (4.64067pt too wide) in paragraph at lines 473--473
[]|\T1/Merriwthr-OsF/m/n/7 (-20) NetMHCpan4.1b|
 []


Package natbib Warning: Citation `mackiewicz1993principal' on page 8
undefined
on input line 487.


Underfull \hbox (badness 10000) in paragraph at lines 555--555
[]|\T1/Merriwthr-OsF/m/n/7 (+20) w/ Trans-former
 []


Underfull \hbox (badness 10000) in paragraph at lines 555--555
[]|\T1/Merriwthr-OsF/m/n/7 (+20) w/ struc-ture
 []
```

```
Underfull \hbox (badness 4765) in paragraph at lines 555--555
\T1/Merriwthr-OsF/m/n/7 (+20) pre-trained em-
 []


Underfull \hbox (badness 10000) in paragraph at lines 555--555
[]|\T1/Merriwthr-OsF/m/n/7 (+20) w/ se-quence
 []


Underfull \hbox (badness 4765) in paragraph at lines 555--555
\T1/Merriwthr-OsF/m/n/7 (+20) pre-trained em-
 []


Underfull \hbox (badness 10000) in paragraph at lines 555--555
[]|\T1/Merriwthr-OsF/m/n/7 (+20) w/ any em-bed-
 []


Underfull \hbox (badness 10000) in paragraph at lines 555--555
[]|\T1/Merriwthr-OsF/m/n/7 (+20) w/ Trans-former
 []


Underfull \hbox (badness 10000) in paragraph at lines 555--555
[]|\T1/Merriwthr-OsF/m/n/7 (+20) w/ struc-ture
 []


Underfull \hbox (badness 4765) in paragraph at lines 555--555
\T1/Merriwthr-OsF/m/n/7 (+20) pre-trained em-
 []


Underfull \hbox (badness 10000) in paragraph at lines 555--555
[]|\T1/Merriwthr-OsF/m/n/7 (+20) w/ se-quence
 []


Underfull \hbox (badness 4765) in paragraph at lines 555--555
\T1/Merriwthr-OsF/m/n/7 (+20) pre-trained em-
 []


Underfull \hbox (badness 10000) in paragraph at lines 555--555
[]|\T1/Merriwthr-OsF/m/n/7 (+20) w/ any em-bed-
 []


Package natbib Warning: Citation `xia2021nepdb' on page 8 undefined on
input li
```

```
ne 558.



[8] [9]

Package natbib Warning: Citation `westhof1984correlation' on page 10
undefined
on input line 576.


Package natbib Warning: Citation `kim2021epitopes' on page 10 undefined
on inpu
t line 576.


Package natbib Warning: Citation `klatt2020solving' on page 10 undefined
on inp
ut line 576.




LaTeX Warning: File `figures/Helix_content.pdf' not found on input line
699.


! Package pdftex.def Error: File `figures/Helix_content.pdf' not found:
using d
raft setting.

See the pdftex.def package documentation for explanation.
Type  H <return>  for immediate help.
 ...

l.699 ...ics[scale=0.7]{figures/Helix_content.pdf}

Try typing  <return>  to proceed.
If that doesn't work, type  X <return>  to quit.


Package natbib Warning: Citation `cock2009biopython' on page 10 undefined
on in
put line 708.


Package natbib Warning: Citation `vihinen1994accuracy' on page 10
undefined on
input line 708.


Package natbib Warning: Citation `chen2016xgboost' on page 10 undefined
on inpu
t line 708.
```

```
[10]

Package natbib Warning: Citation `marino2022visualizing' on page 11
undefined o
n input line 717.


Package natbib Warning: Citation `anjana2012aromatic' on page 11
undefined on i
nput line 717.


Package natbib Warning: Citation `baker2012hydrogen' on page 11 undefined
on in
put line 717.


Package natbib Warning: Citation `grimaldi2015stability' on page 11
undefined o
n input line 717.


Package natbib Warning: Citation `drelich2011hydrophilic' on page 11
undefined
on input line 717.


Package natbib Warning: Citation `marino2022visualizing' on page 11
undefined o
n input line 728.


Package natbib Warning: Citation `anjana2012aromatic' on page 11
undefined on i
nput line 728.


Package natbib Warning: Citation `perticaroli2013secondary' on page 11
undefine
d on input line 728.


Package natbib Warning: Citation `mamonova2013stability' on page 11
undefined o
n input line 728.



Underfull \vbox (badness 2237) has occurred while \output is active []
```

```
[11]
Underfull \hbox (badness 10000) in paragraph at lines 762--763
[]\T1/Merriwthr-OsF/m/up/7.5 (+20) Project home page: []$\T1/lmtt/m/n/7.5
https
 : / / github . com / SkywalkerLuke /
 []


Package natbib Warning: Citation `vita2019immune' on page 12 undefined on
input
 line 780.


Package natbib Warning: Citation `kocsalouglu2023cancer' on page 12
undefined o
n input line 780.


Package natbib Warning: Citation `shugay2018vdjdb' on page 12 undefined
on inpu
t line 780.


Package natbib Warning: Citation `nolan2020large' on page 12 undefined on
input
 line 780.


Package natbib Warning: Citation `lu2022dbpepneo2' on page 12 undefined
on inpu
t line 780.


Package natbib Warning: Citation `xia2021nepdb' on page 12 undefined on
input l
ine 780.


Package natbib Warning: Citation `https://doi.org/10.5524/102633' on page
12 un
defined on input line 780.


Package natbib Warning: Citation `TransHLA2024' on page 12 undefined on
input l
ine 780.

No file main.bbl.

Package natbib Warning: There were undefined citations.
```

```
[12

]
enddocument/afterlastpage: lastpage setting LastPage.
(./main.aux)
 ***********
LaTeX2e <2024-06-01> patch level 2
L3 programming layer <2020/03/25>
 ***********

LaTeX Font Warning: Size substitutions with differences
(Font)              up to 1.0pt have occurred.


LaTeX Font Warning: Some font shapes were not available, defaults
substituted.

Package rerunfilecheck Info: File `main.out' has not changed.
(rerunfilecheck)             Checksum:
40C4E8BABD049C3D6232BBEC3599D30D;5668.
 )
Here is how much of TeX's memory you used:
 35819 strings out of 473583
 726593 string characters out of 5732343
 2020908 words of memory out of 5000000
 57369 multiletter control sequences out of 15000+600000
 1993875 words of font info for 607 fonts, out of 8000000 for 9000
 1141 hyphenation exceptions out of 8191
 123i,13n,131p,2893b,985s stack positions out of
10000i,1000n,20000p,200000b,200000s
<c:/texlive/2024/texmf-dist/fonts/type1/sorkin/merriweather/Merriwthr-
Bold.pf
b><c:/texlive/2024/texmf-dist/fonts/type1/sorkin/merriweather/Merriwthr-
BoldIta
lic.pfb><c:/texlive/2024/texmf-
dist/fonts/type1/sorkin/merriweather/Merriwthr-I
talic.pfb><c:/texlive/2024/texmf-
dist/fonts/type1/sorkin/merriweather/Merriwthr
-Regular.pfb><c:/texlive/2024/texmf-
dist/fonts/type1/sorkin/merriweather/Merriw
thrSans-Regular.pfb><c:/texlive/2024/texmf-
dist/fonts/type1/public/amsfonts/cme
xtra/cmex7.pfb><c:/texlive/2024/texmf-
dist/fonts/type1/public/amsfonts/cm/cmsy6
.pfb><c:/texlive/2024/texmf-
dist/fonts/type1/public/amsfonts/cm/cmsy7.pfb><c:/t
exlive/2024/texmf-
dist/fonts/type1/public/amsfonts/euler/euex8.pfb><c:/texlive/
2024/texmf-
dist/fonts/type1/public/amsfonts/euler/eurm7.pfb><c:/texlive/2024/te
xmf-dist/fonts/type1/public/lm/lmtt8.pfb>
```

```
Output written on main.pdf (12 pages, 536645 bytes).
PDF statistics:
 350 PDF objects out of 1000 (max. 8388607)
 297 compressed objects within 3 object streams
 68 named destinations out of 1000 (max. 500000)
 226519 words of extra memory for PDF output out of 266212 (max.
10000000)
```

Click here to access/download;LaTeX - Figure (eps, ps, etc.);flexibility_property.pdf

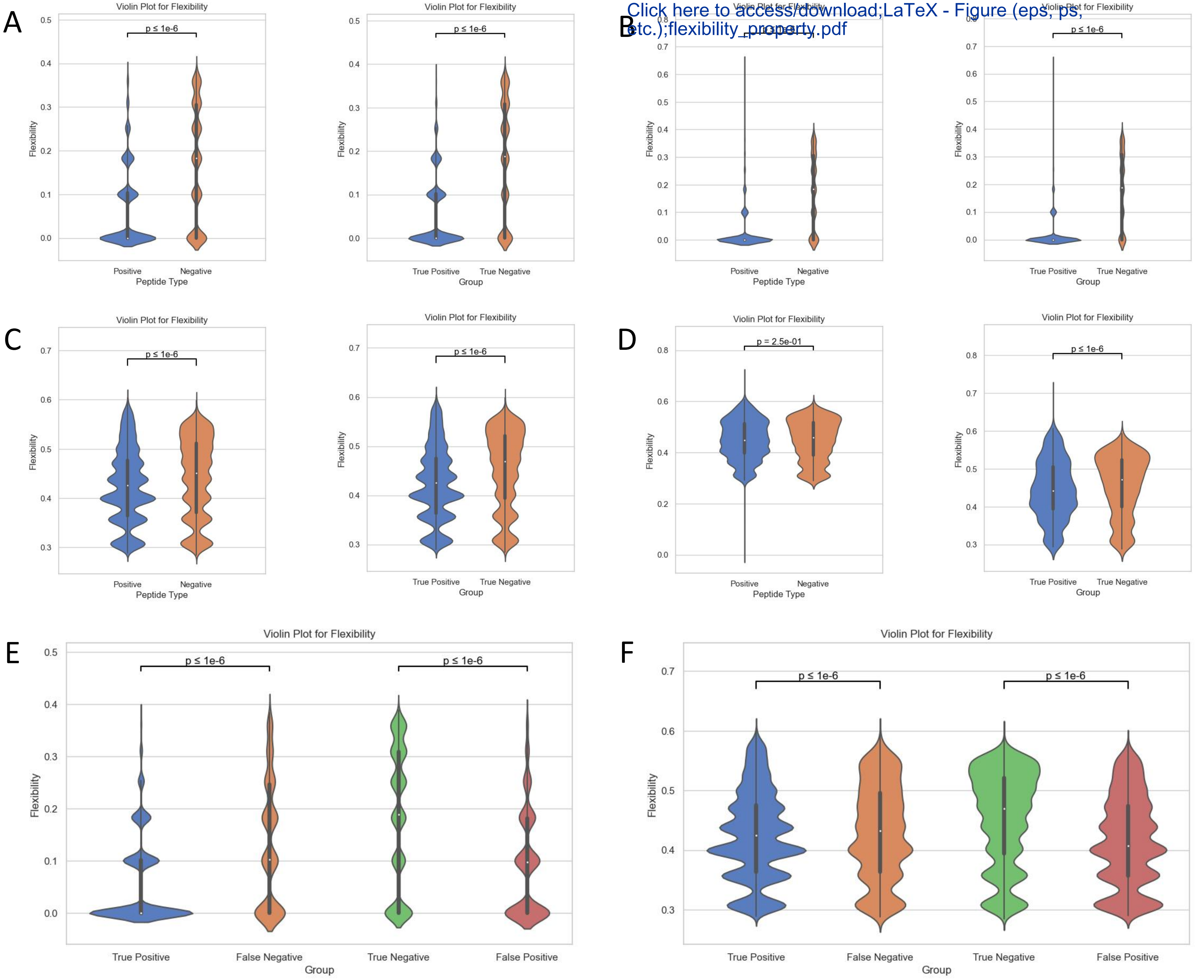

Click here to access/download;LaTeX - Figure (eps, ps, etc.);Helix_content.pdf

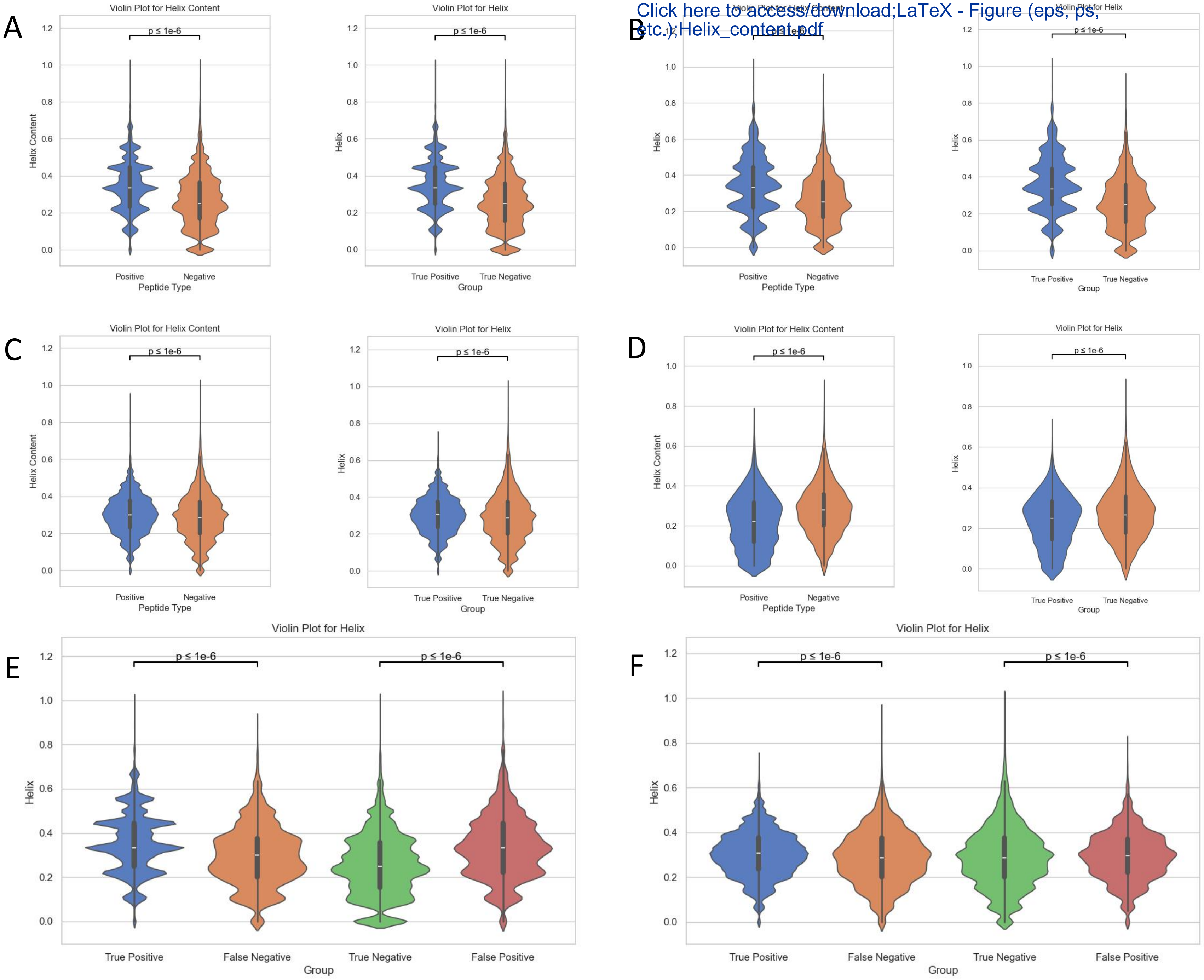

Click here to access/download;LaTeX - Figure (eps, ps, etc.);HLA_I_allele_frequency.pdf

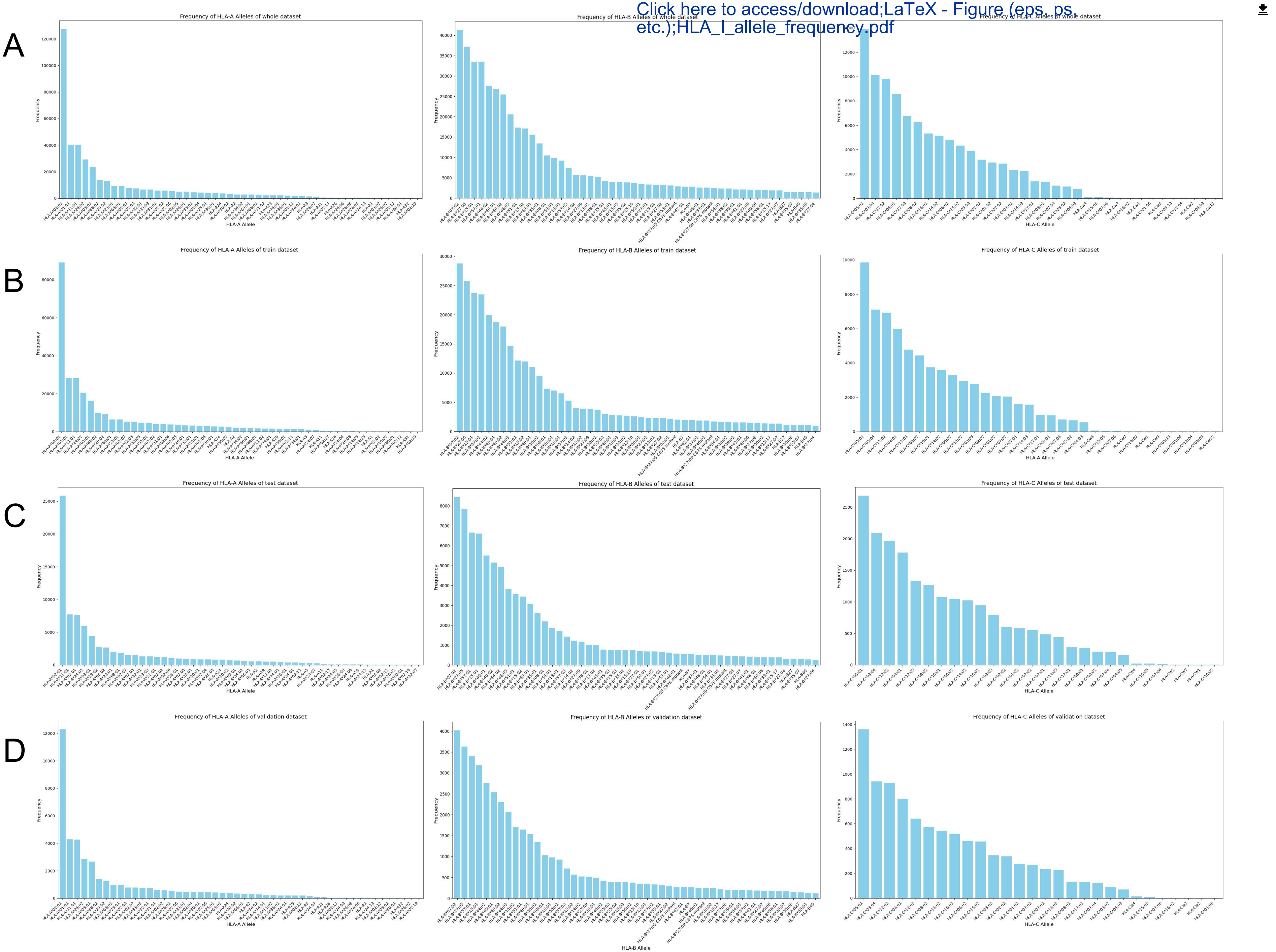

Click here to access/download;LaTeX - Figure (eps, ps, etc.);HLA_I_allele_frequency.png

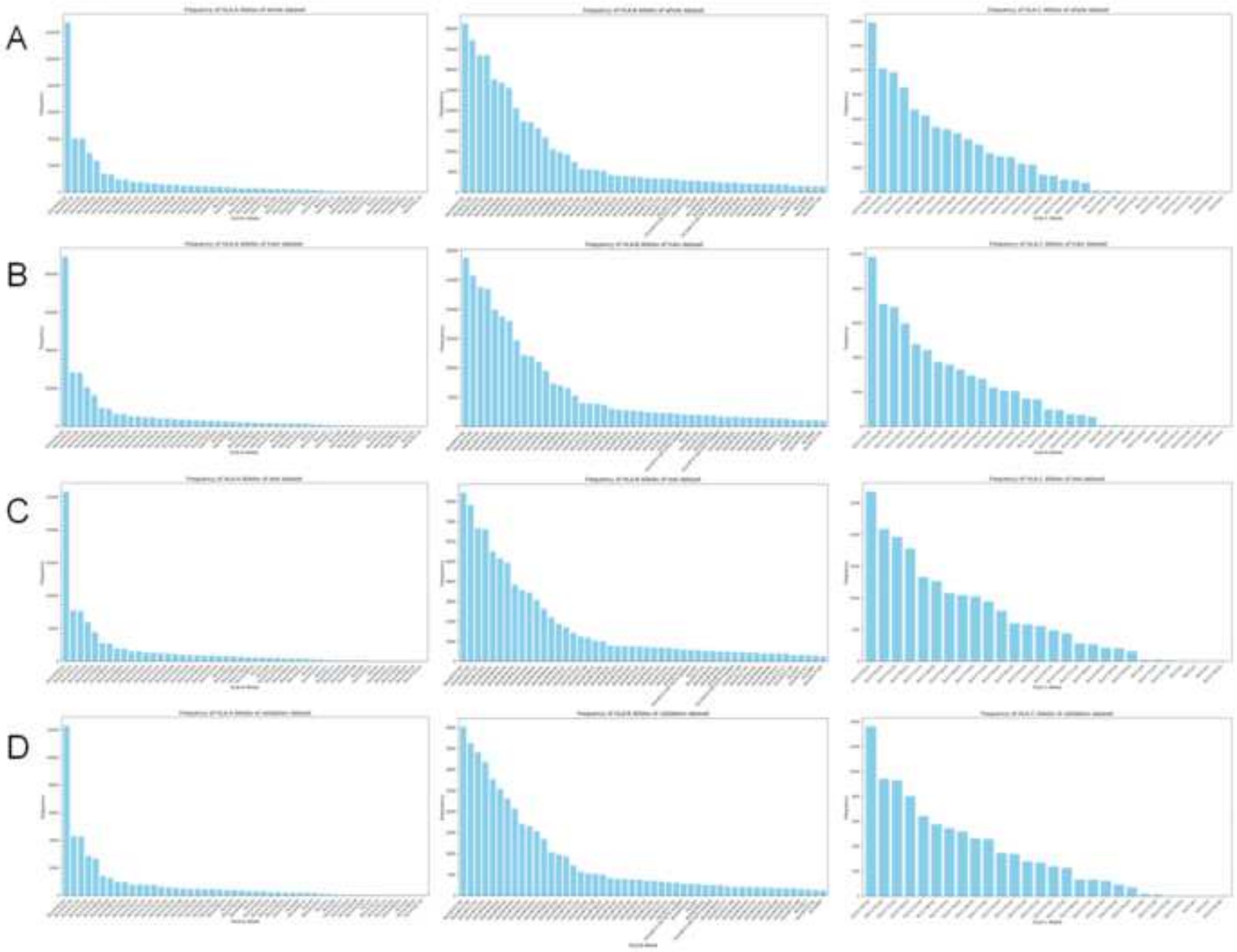

Click here to access/download;LaTeX - Figure (eps, ps, etc.);HLA_II_allele_frequency.pdf

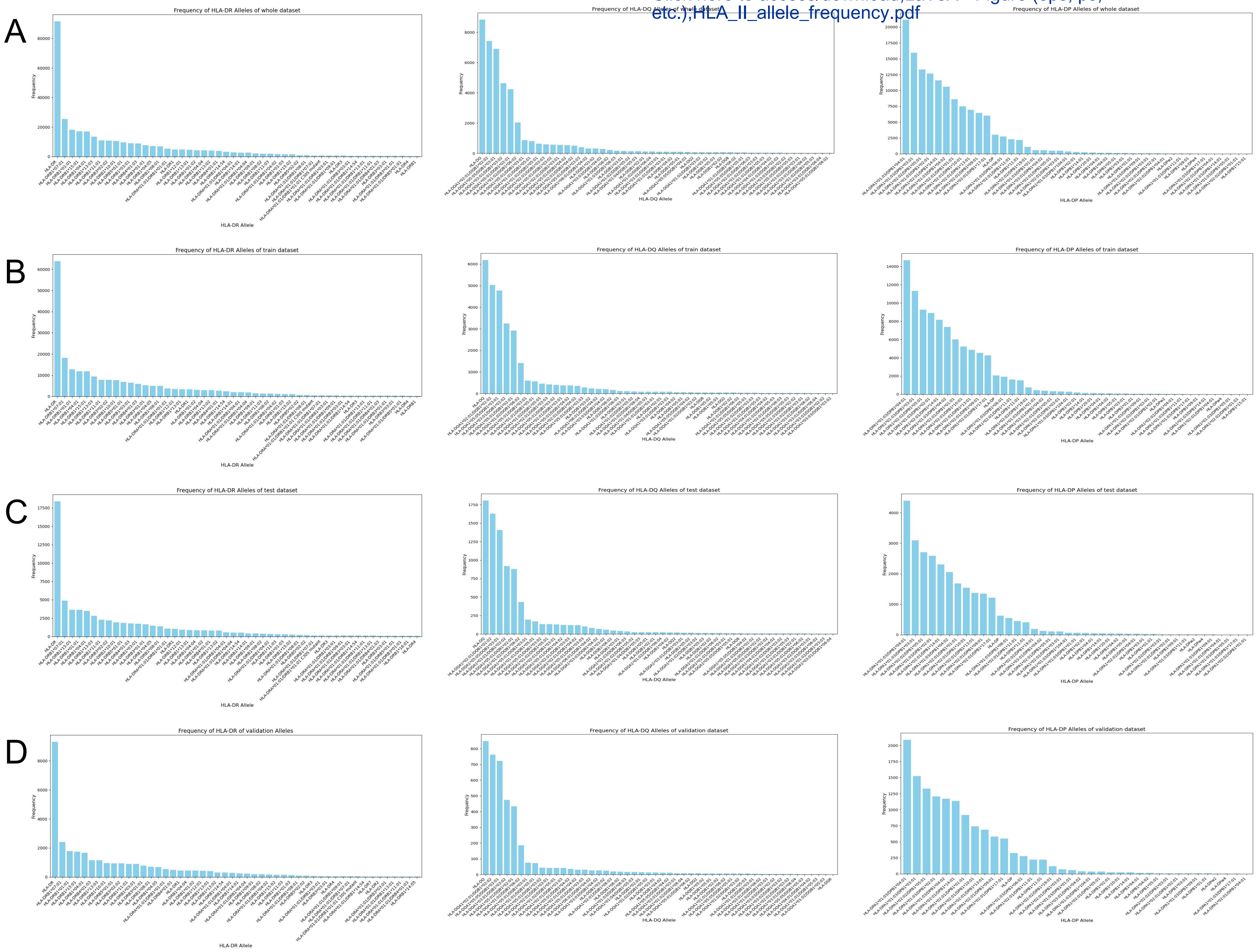

Click here to access/download;LaTeX - Figure (eps, ps, etc.);HLA_II_allele_frequency.png

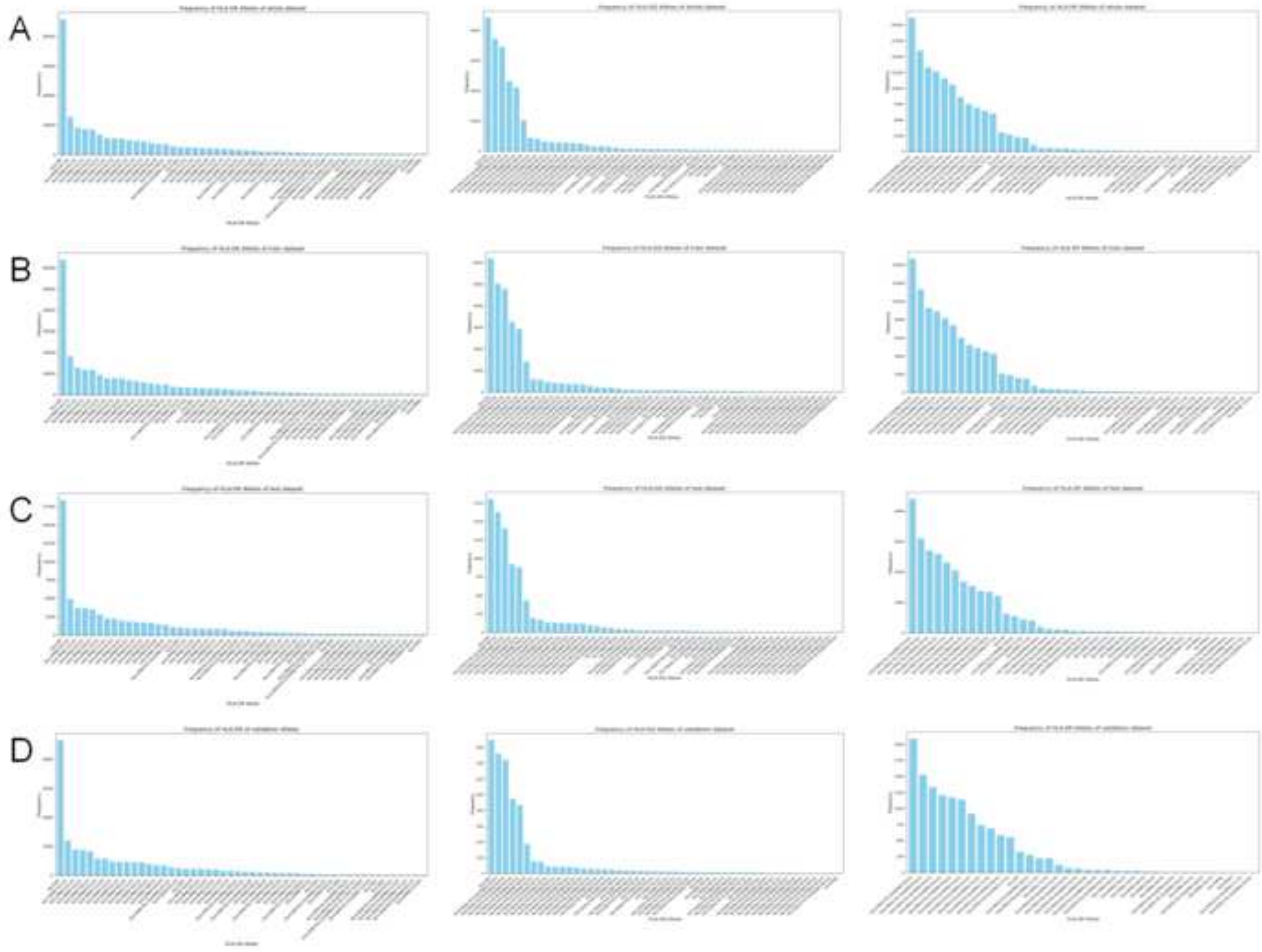

Click here to access/download;LaTeX - Figure (eps, ps, etc.);main_figure _2.pdf

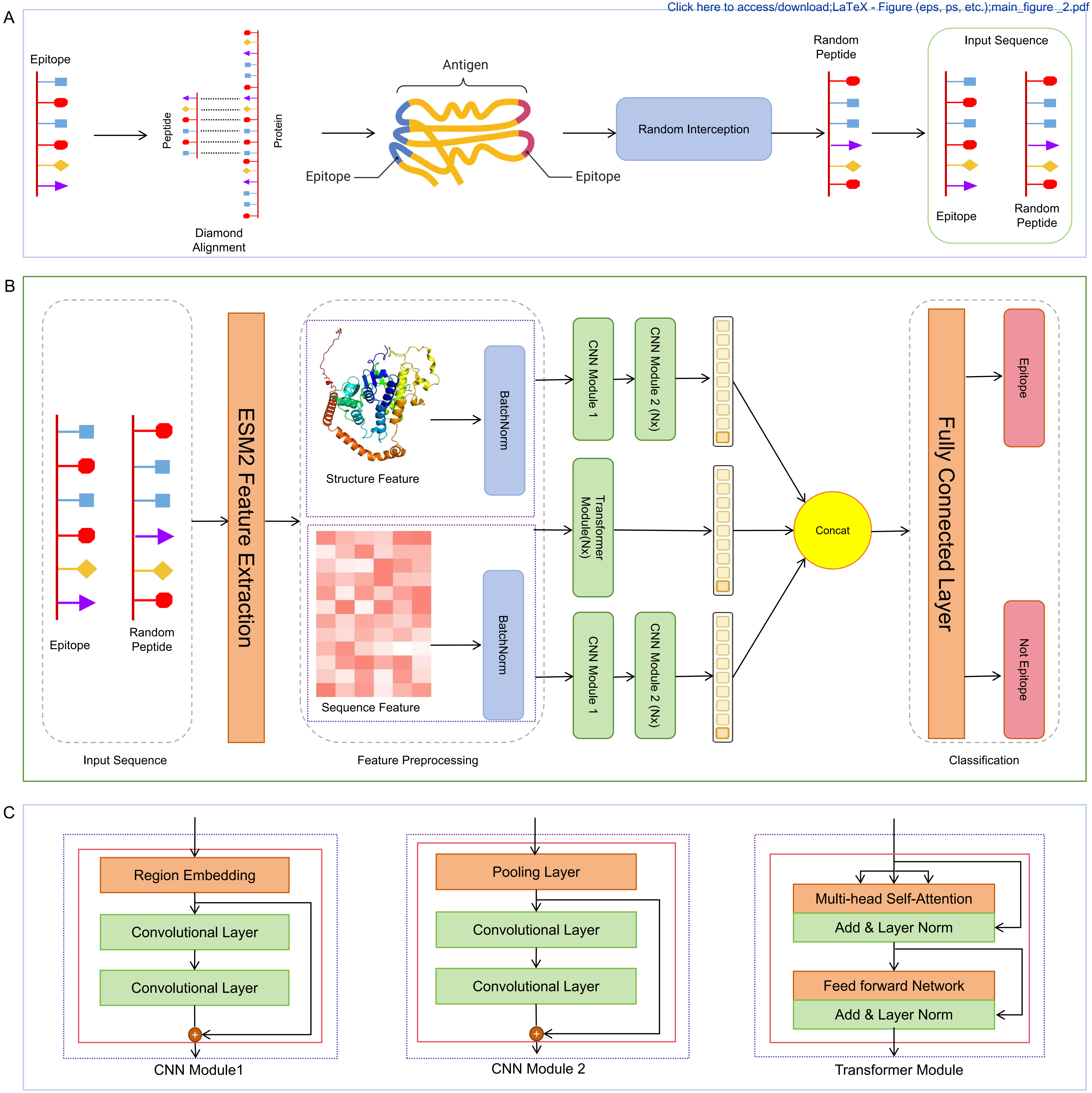

Click here to access/download;LaTeX - Figure (eps, ps, etc.);main_figure.pdf

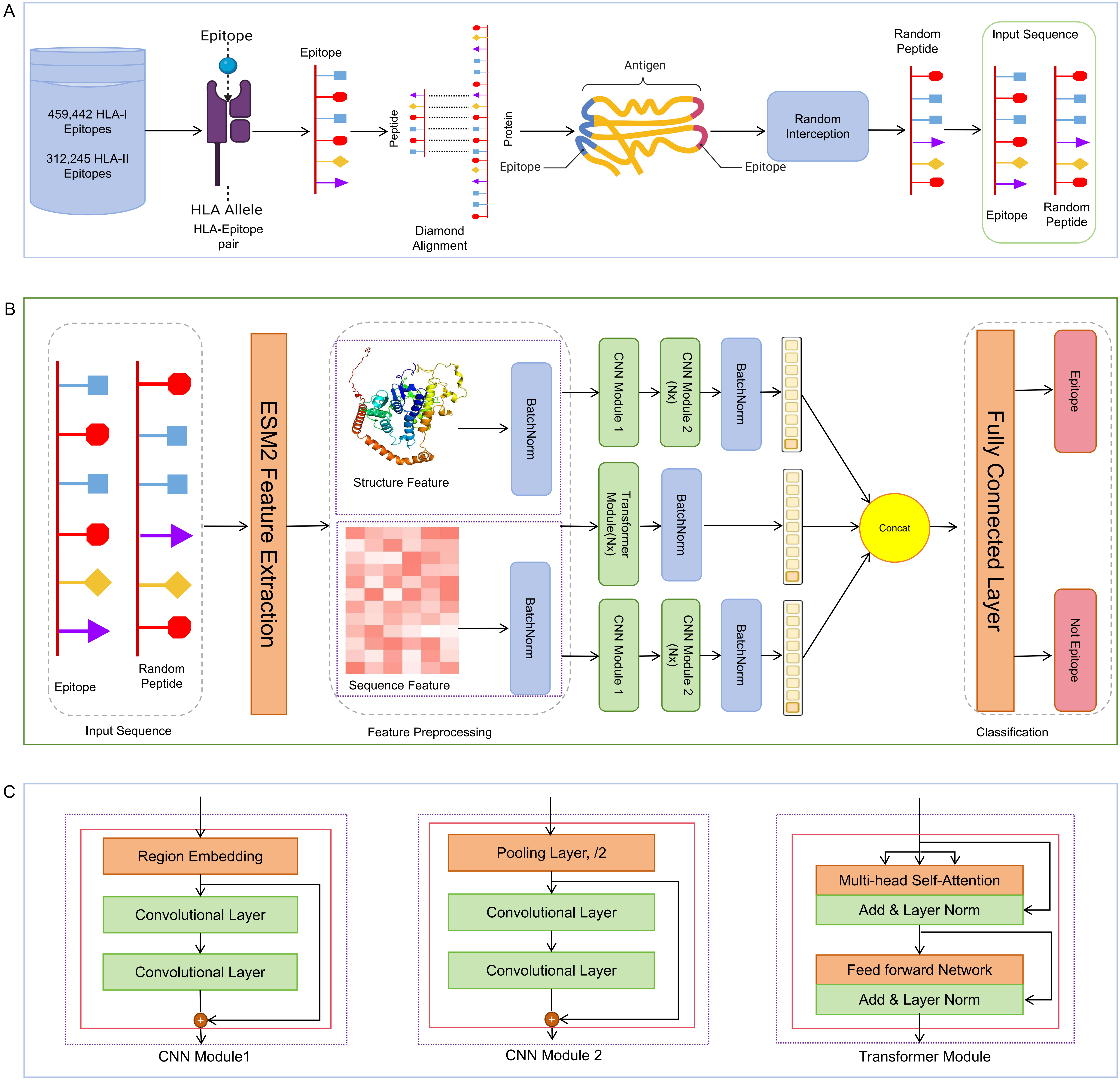

Click here to access/download;LaTeX - Figure (eps, ps, etc.);PCA_embedding.pdf

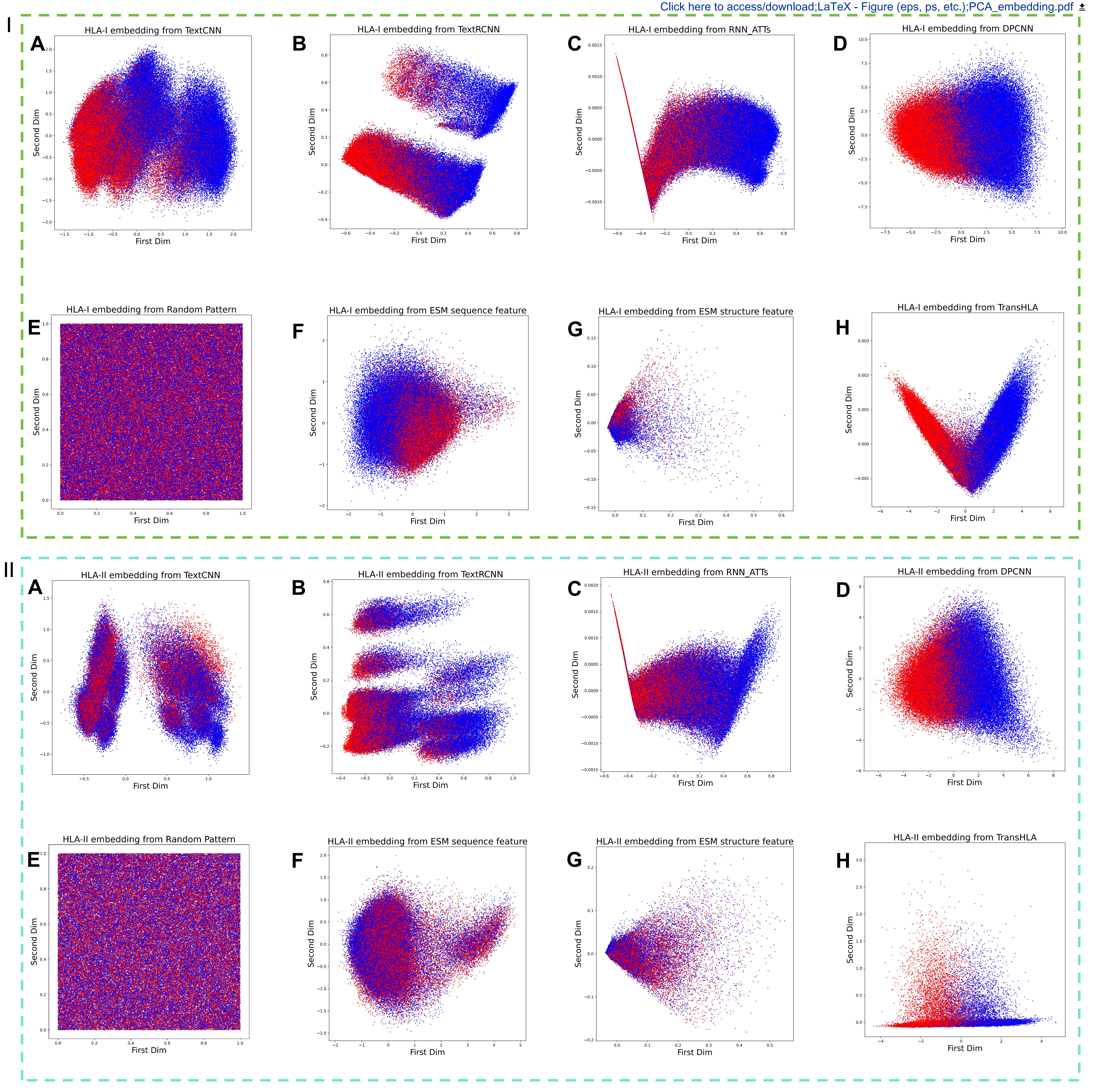

Click here to access/download;LaTeX - Figure (eps, ps, etc.);Ablation_study (1).pdf

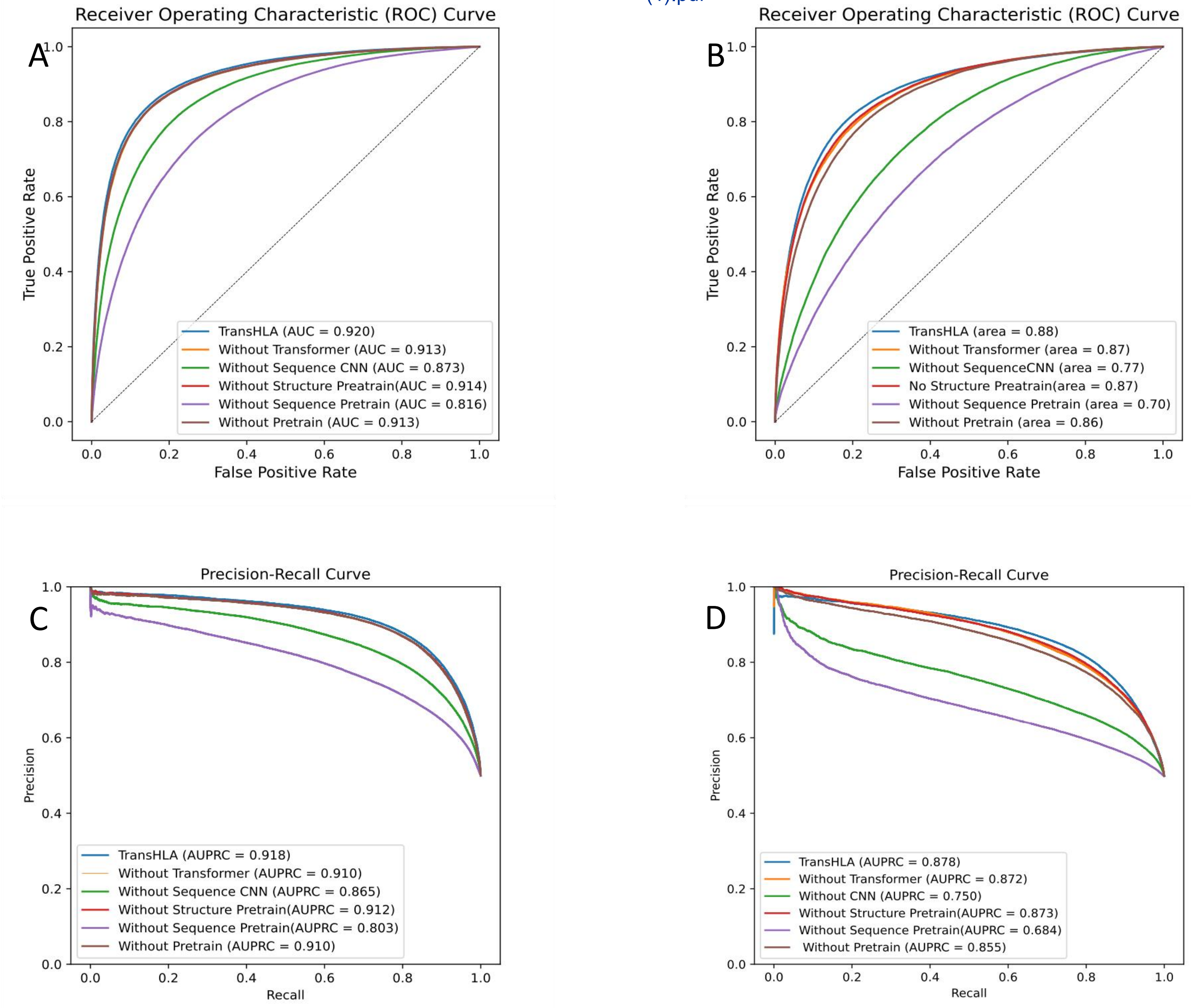

Click here to access/download;LaTeX - Figure (eps, ps, etc.);Compare_study.pdf

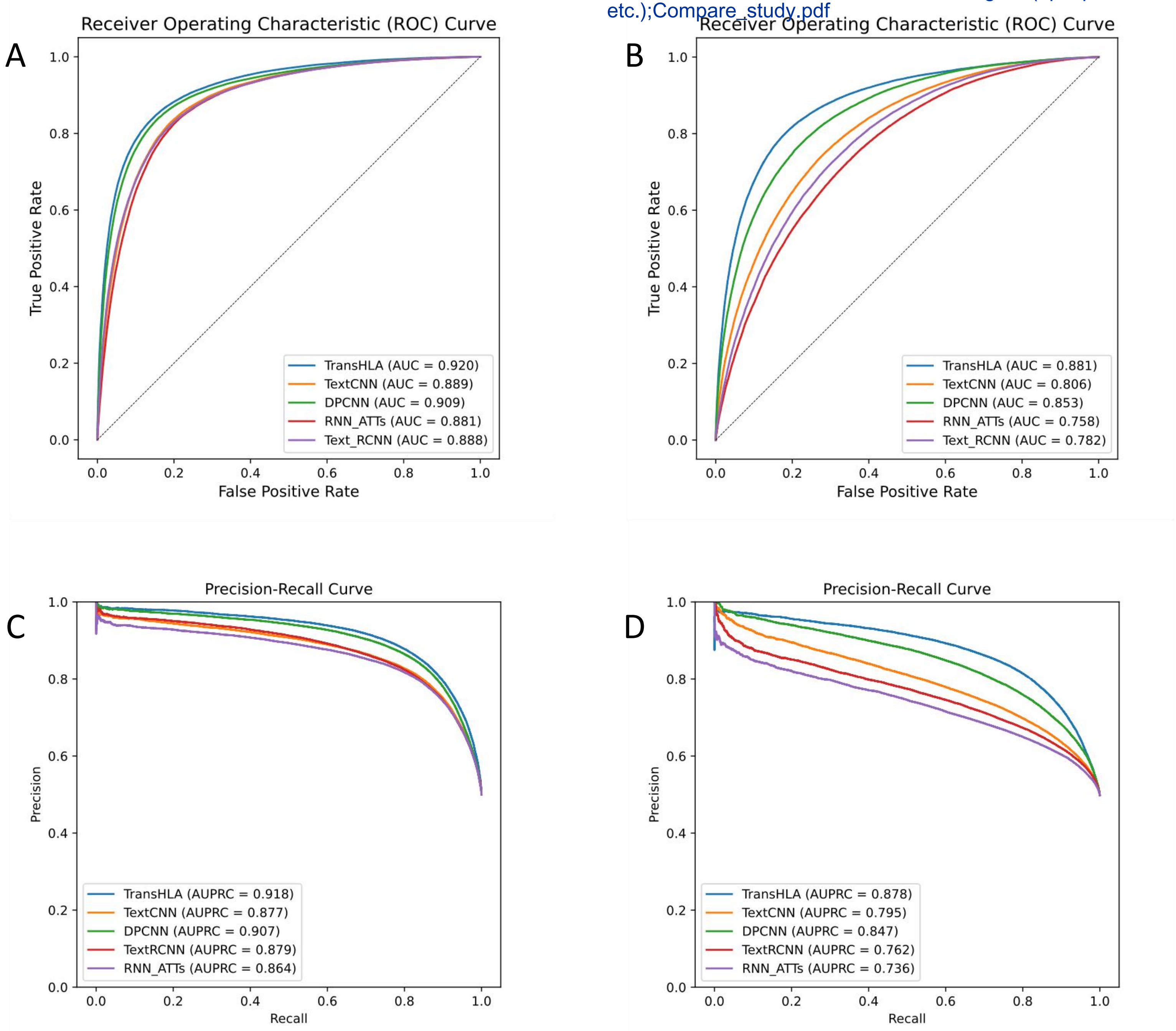

Click here to access/download;LaTeX - Figure (eps, ps, etc.);Confusion_matrix.pdf

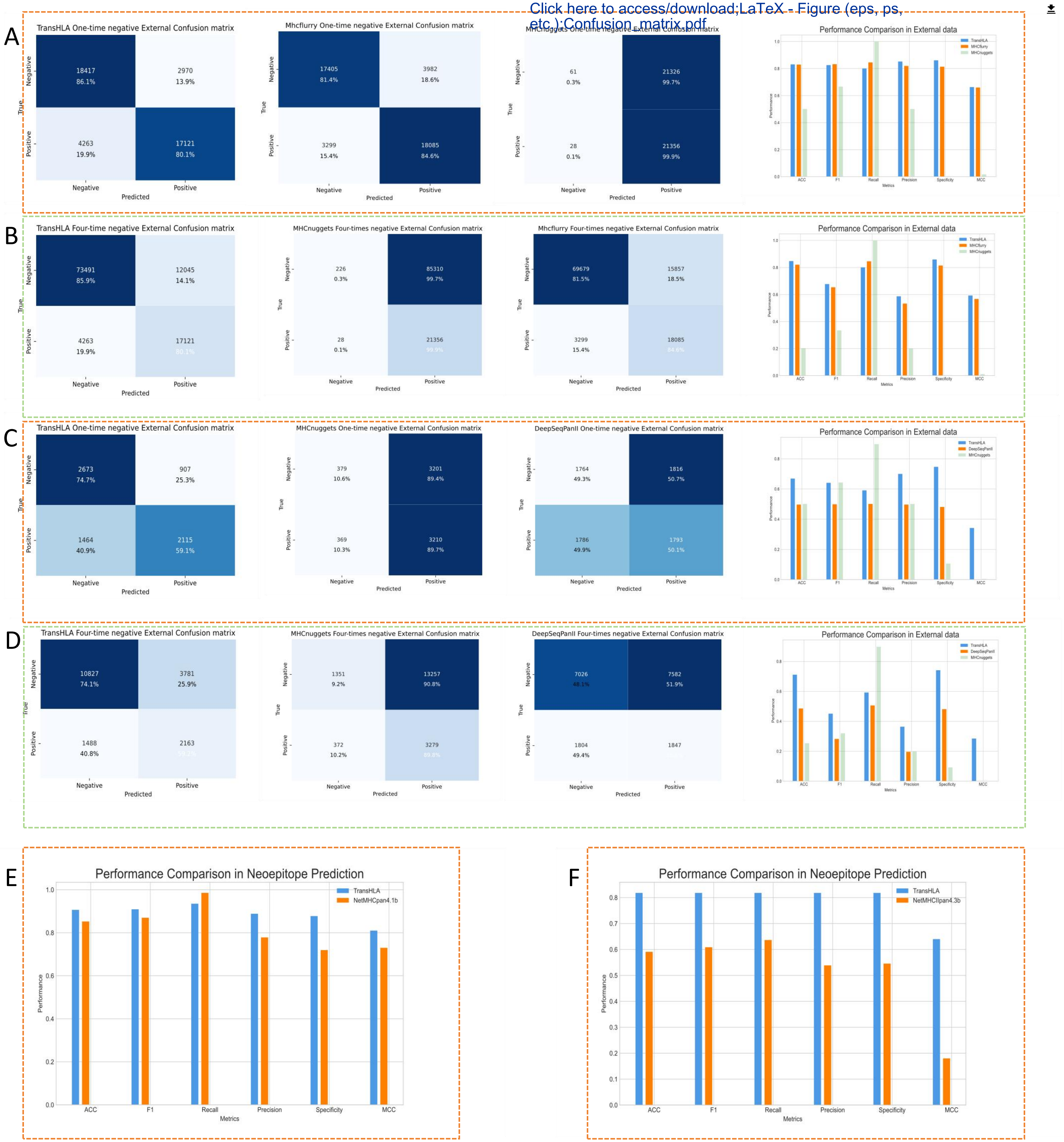

Click here to access/download;LaTeX - Figure (eps, ps, etc.);example-grid-100x100pt.png

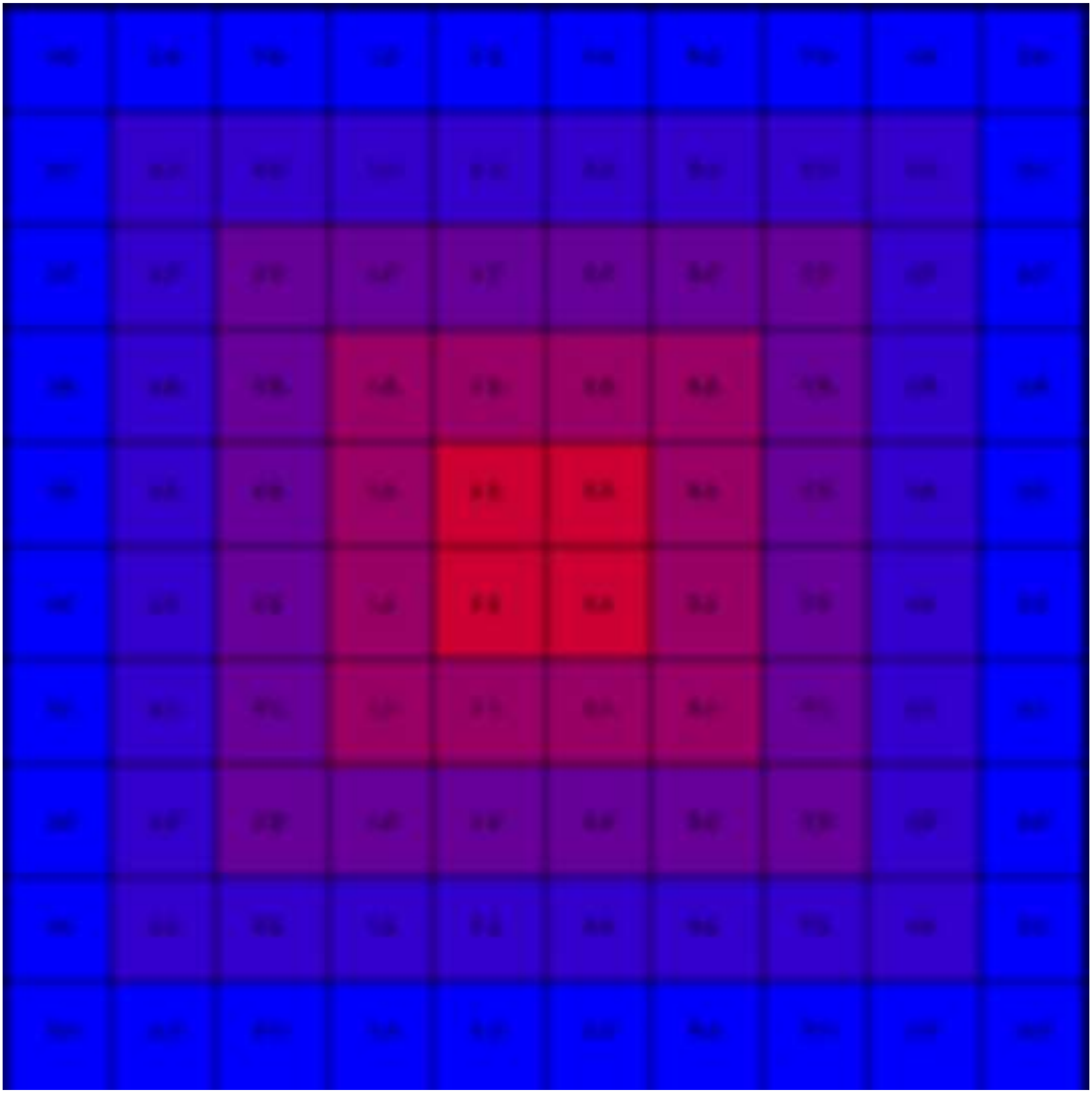

Click here to access/download;LaTeX - Figure (eps, ps, etc.);example-image.png

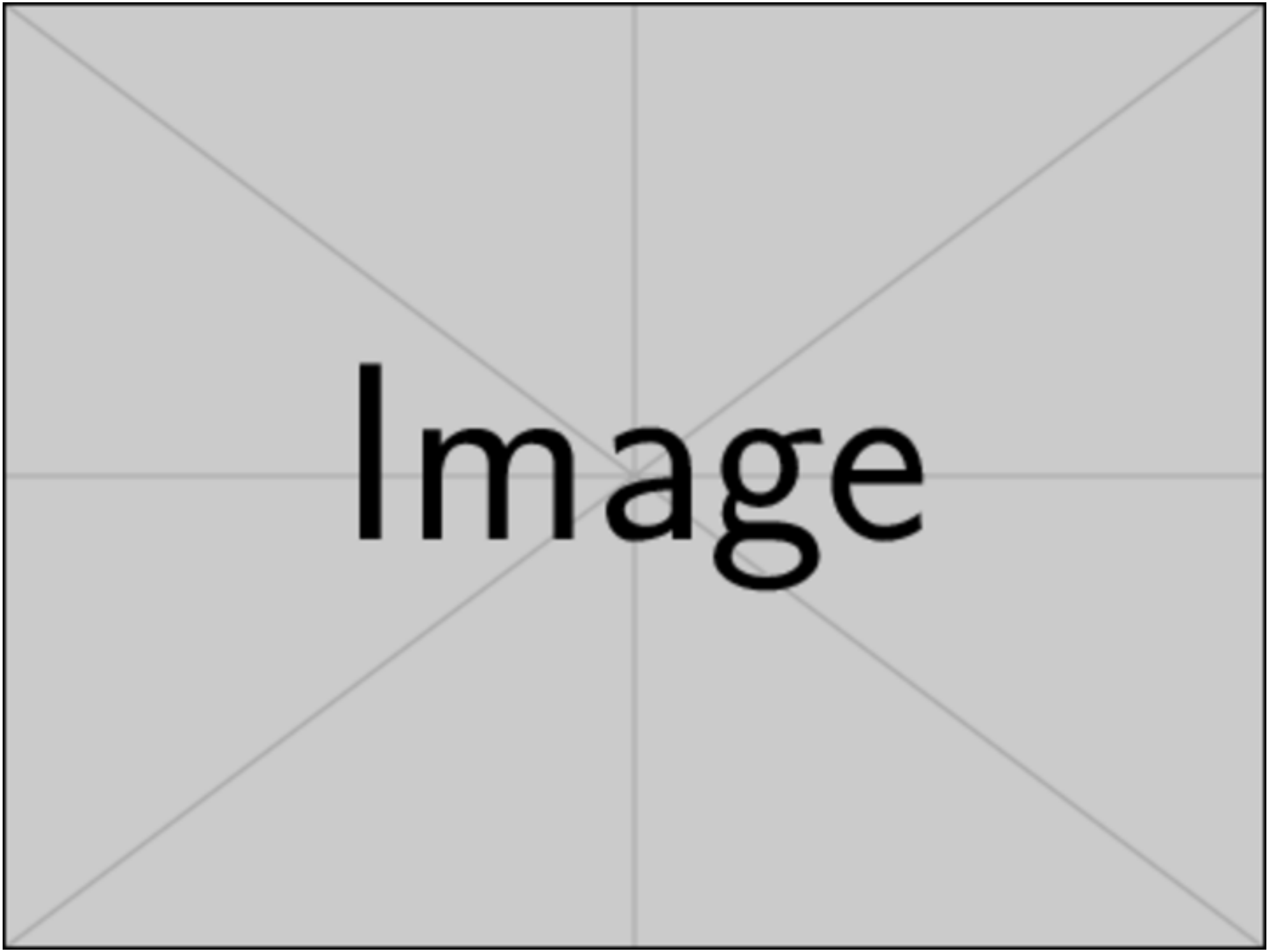

Supplementary Material

Click here to access/download;LaTeX - Figure (eps, ps, etc.);supplementary file.pdf

Supplementary file 1

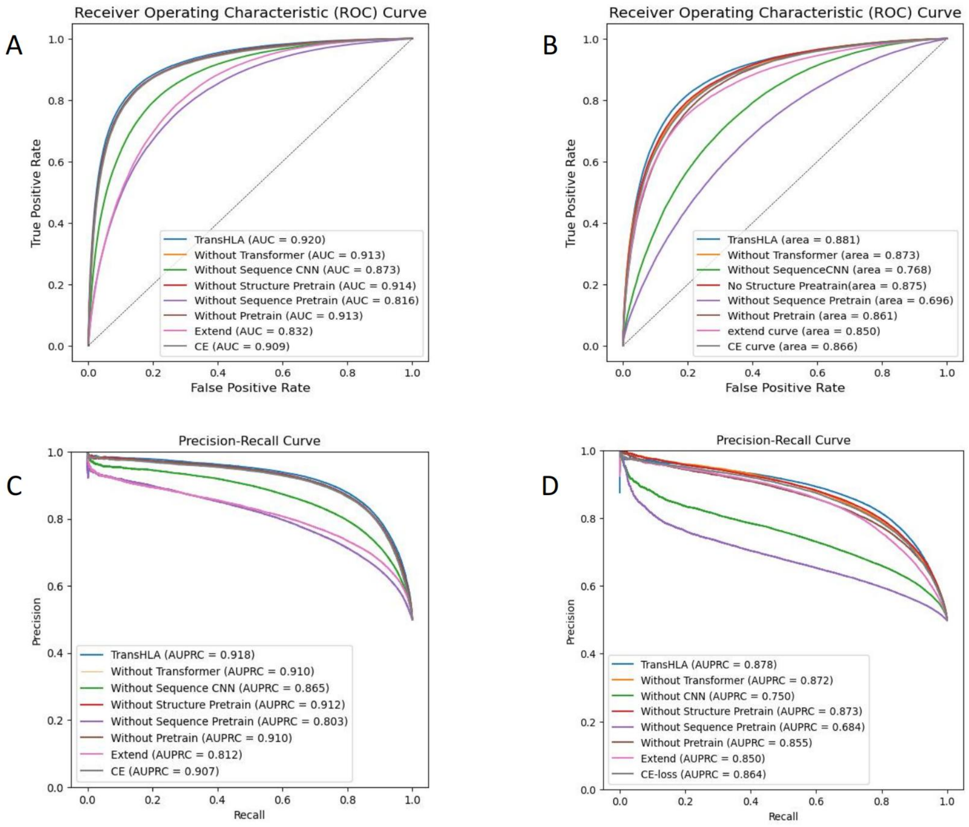

Supplementary Figure 1. The figure presents an ablation study for our predictive model in different modules, showing how performance metrics—AUROC and AUPRC—are affected by the removal of specific modules for both HLA-I (Subfigures A and C) and HLA-II (Subfigures B and D) classes. Changes in these metrics underscore the contribution of each component to the model's accuracy in epitope prediction, providing insight into the model's architecture and the pivotal elements for its effectiveness across HLA classes.

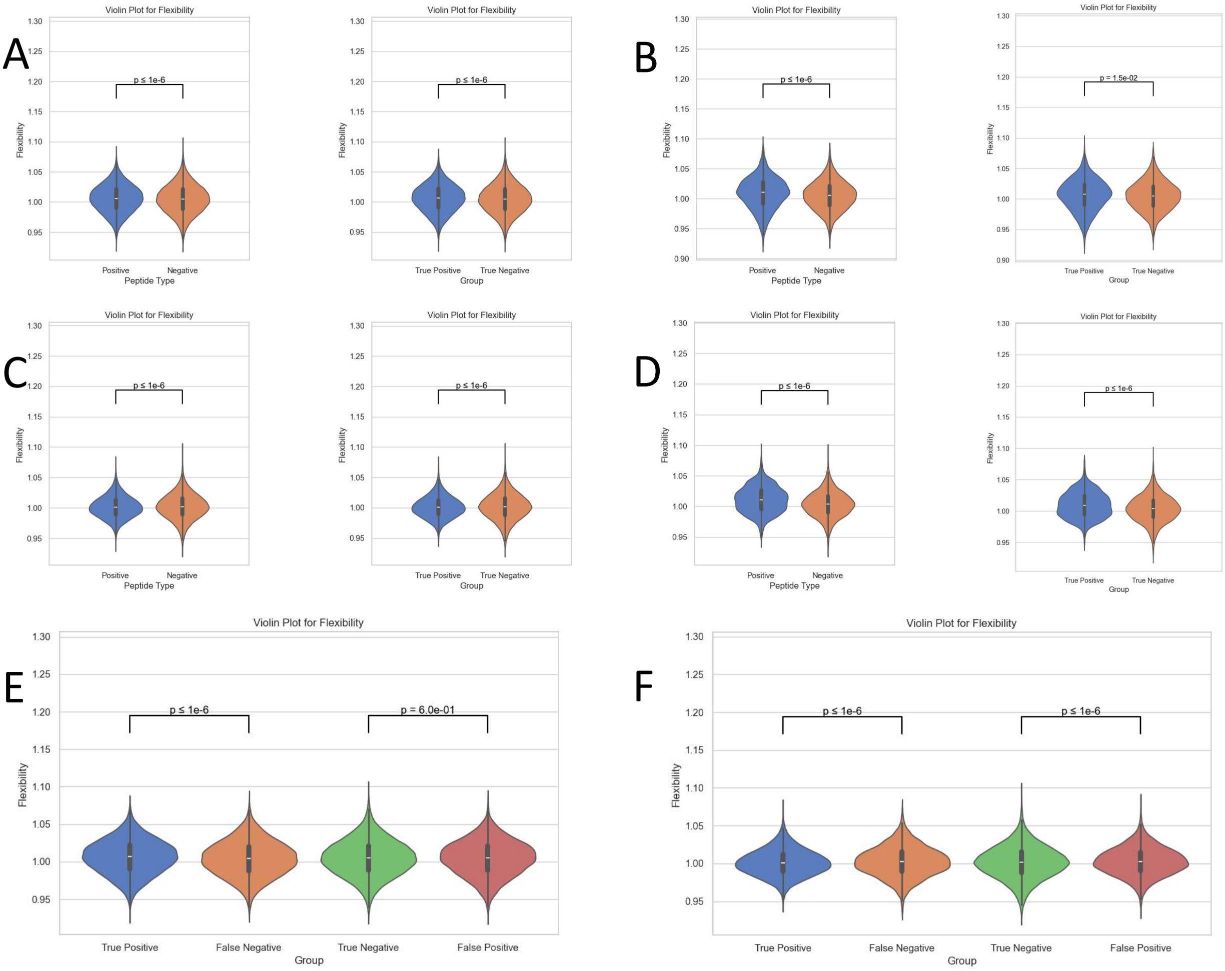

Supplementary Figure 2. This comprehensive figure presents a series of violin plots illustrating the 'Flexibility' chemical property of peptides across various sample subsets for HLA-I and HLA-II molecules. Subfigure (A) delineates the Flexibility distribution in independent test samples for HLA-I, separated into positive and negative samples, with each subgroup's statistical significance assessed via t-tests and annotated with corresponding p-values. Subfigure (C) mirrors this setup for HLA-II independent test samples, highlighting the comparative Flexibility distributions. The external dataset distributions for HLA-I and HLA-II are respectively showcased in subfigures (B) and (D), emphasizing the metric's external validity. Subfigures (E) and (F) delve deeper, contrasting the Flexibility of true positives and false negatives against true negatives and false positives within HLA-I and HLA-II datasets, respectively.

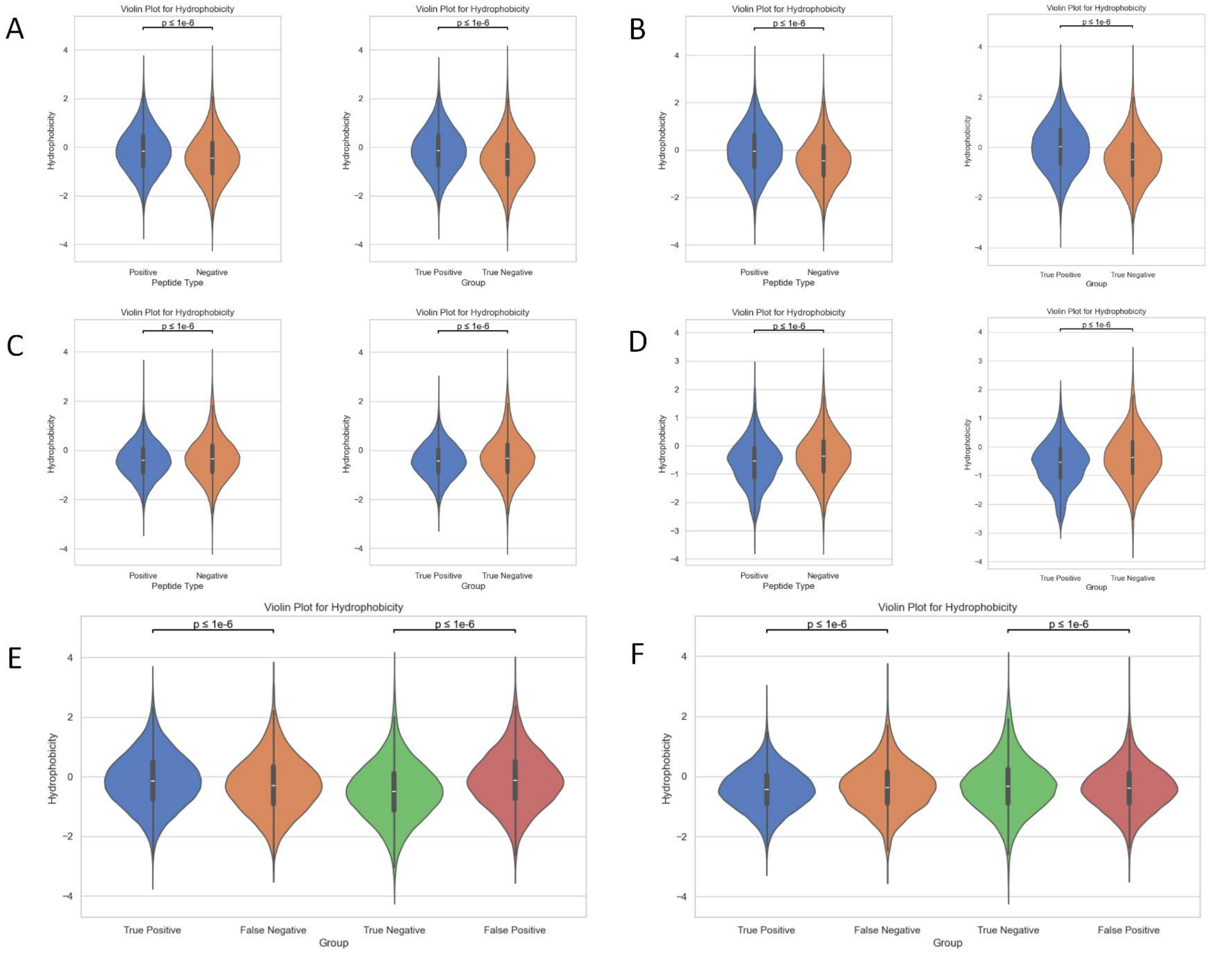

Supplementary Figure 3. This comprehensive figure presents a series of violin plots illustrating the 'Hydrophobicity' chemical property of peptides across various sample subsets for HLA-I and HLA-II molecules. Subfigure (A) delineates the Hydrophobicity distribution in independent test samples for HLA-I, separated into positive and negative samples, with each subgroup's statistical significance assessed via t-tests and annotated with corresponding p-values. Subfigure (C) mirrors this setup for HLA-II independent test samples, highlighting the comparative Hydrophobicity distributions. The external dataset distributions for HLA-I and HLA-II are respectively showcased in subfigures (B) and (D), emphasizing the metric's external validity. Subfigures (E) and (F) delve deeper, contrasting the Hydrophobicity of true positives and false negatives against true negatives and false positives within HLA-I and HLA-II datasets, respectively.

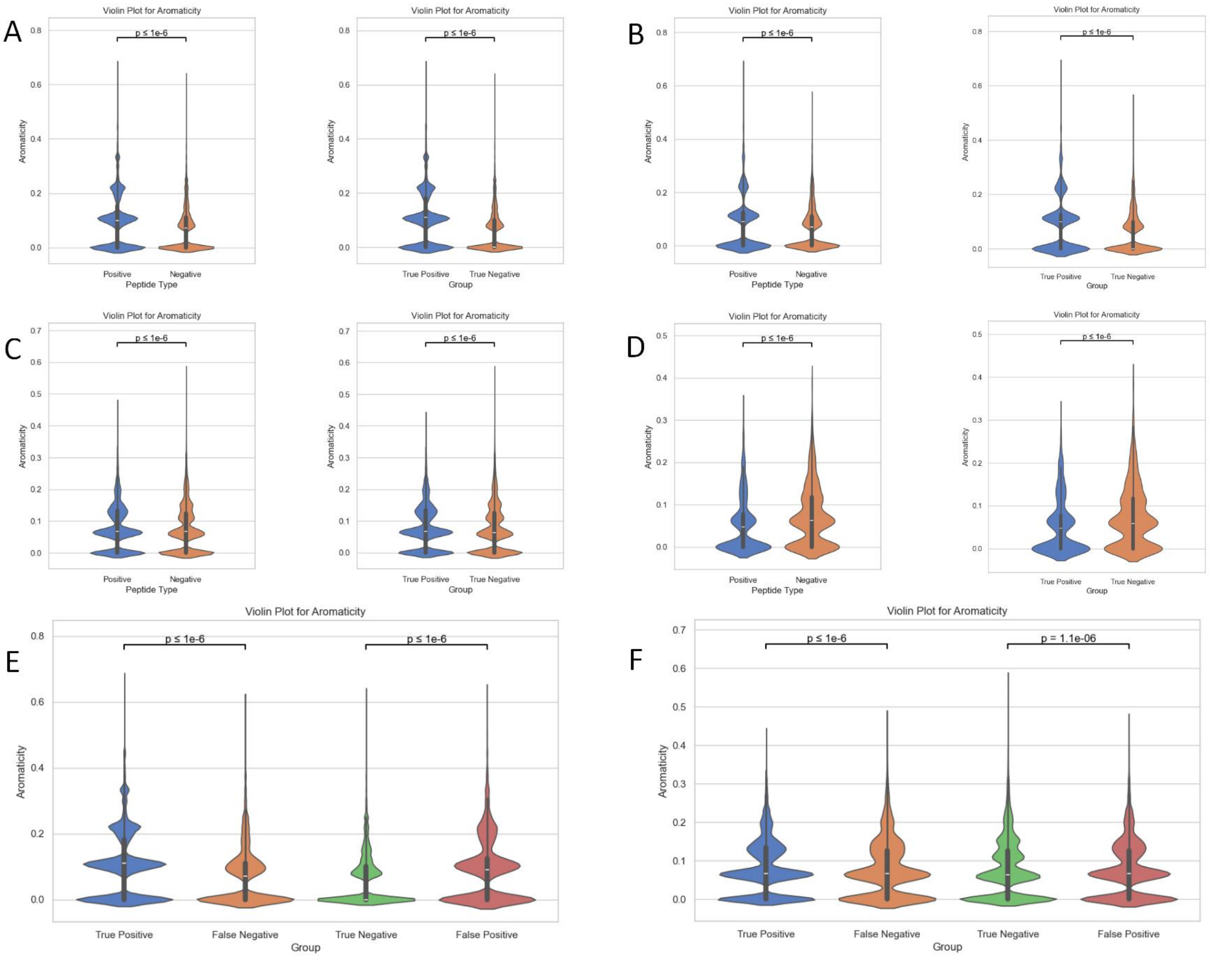

Supplementary Figure 4. This comprehensive figure presents a series of violin plots illustrating the 'Aromaticity' chemical property of peptides across various sample subsets for HLA-I and HLA-II molecules. Subfigure (A) delineates the Aromaticity distribution in independent test samples for HLA-I, separated into positive and negative samples, with each subgroup's statistical significance assessed via t-tests and annotated with corresponding p-values. Subfigure (C) mirrors this setup for HLA-II independent test samples, highlighting the comparative Aromaticity distributions. The external dataset distributions for HLA-I and HLA-II are respectively showcased in subfigures (B) and (D), emphasizing the metric's external validity. Subfigures (E) and (F) delve deeper, contrasting the Aromaticity of true positives and false negatives against true negatives and false positives within HLA-I and HLA-II datasets, respectively.

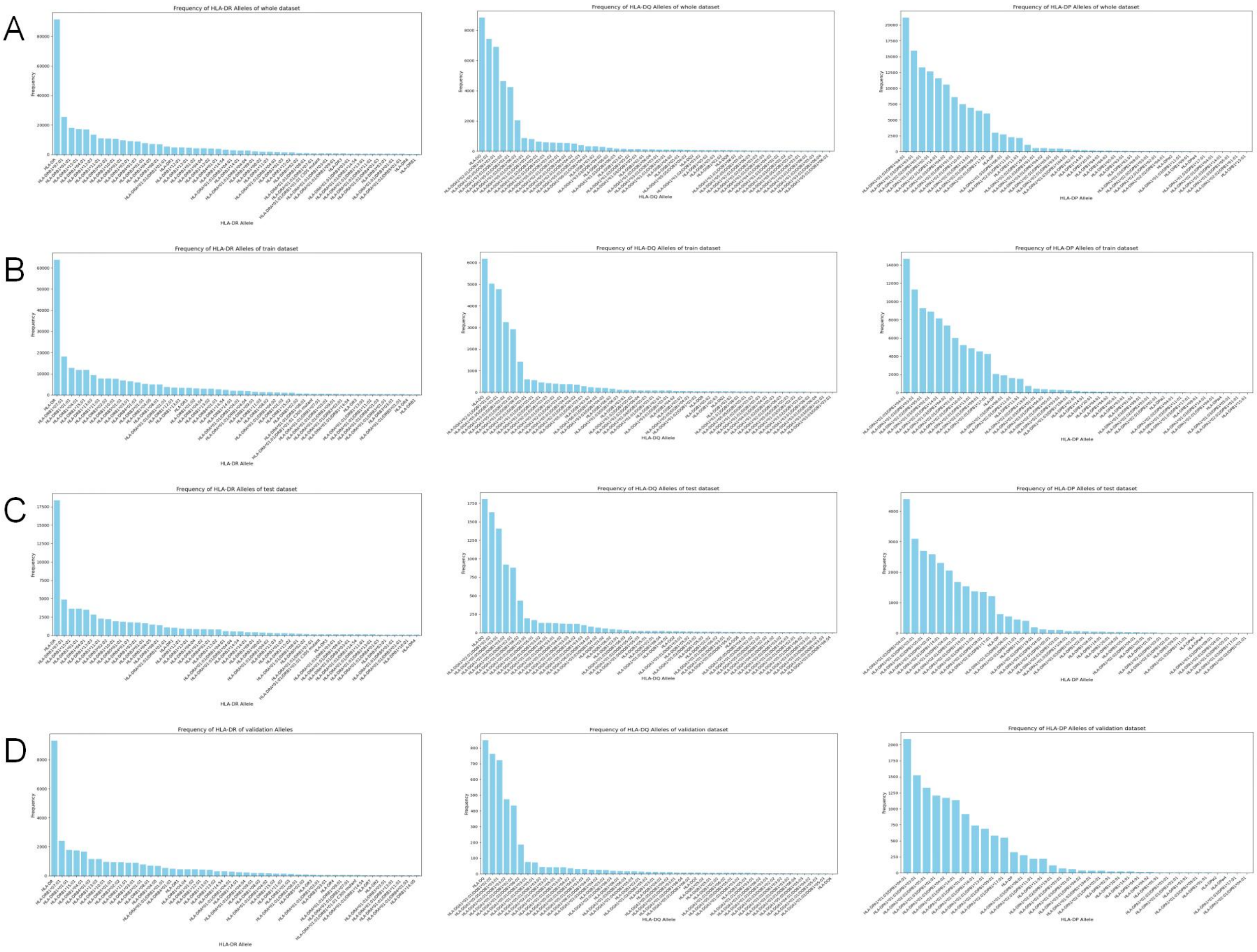

Supplementary Figure 5. This figure illustrates the experimental distribution of major HLA-II alleles in the IEDB. We plotted the alleles by type, where subfigure A shows the distribution for all data, B for the training dataset, C for the test dataset, and D for the validation dataset. Left represents HLA-DR alleles, middle represents HLA-DQ alleles, and right represents HLA-DP alleles.

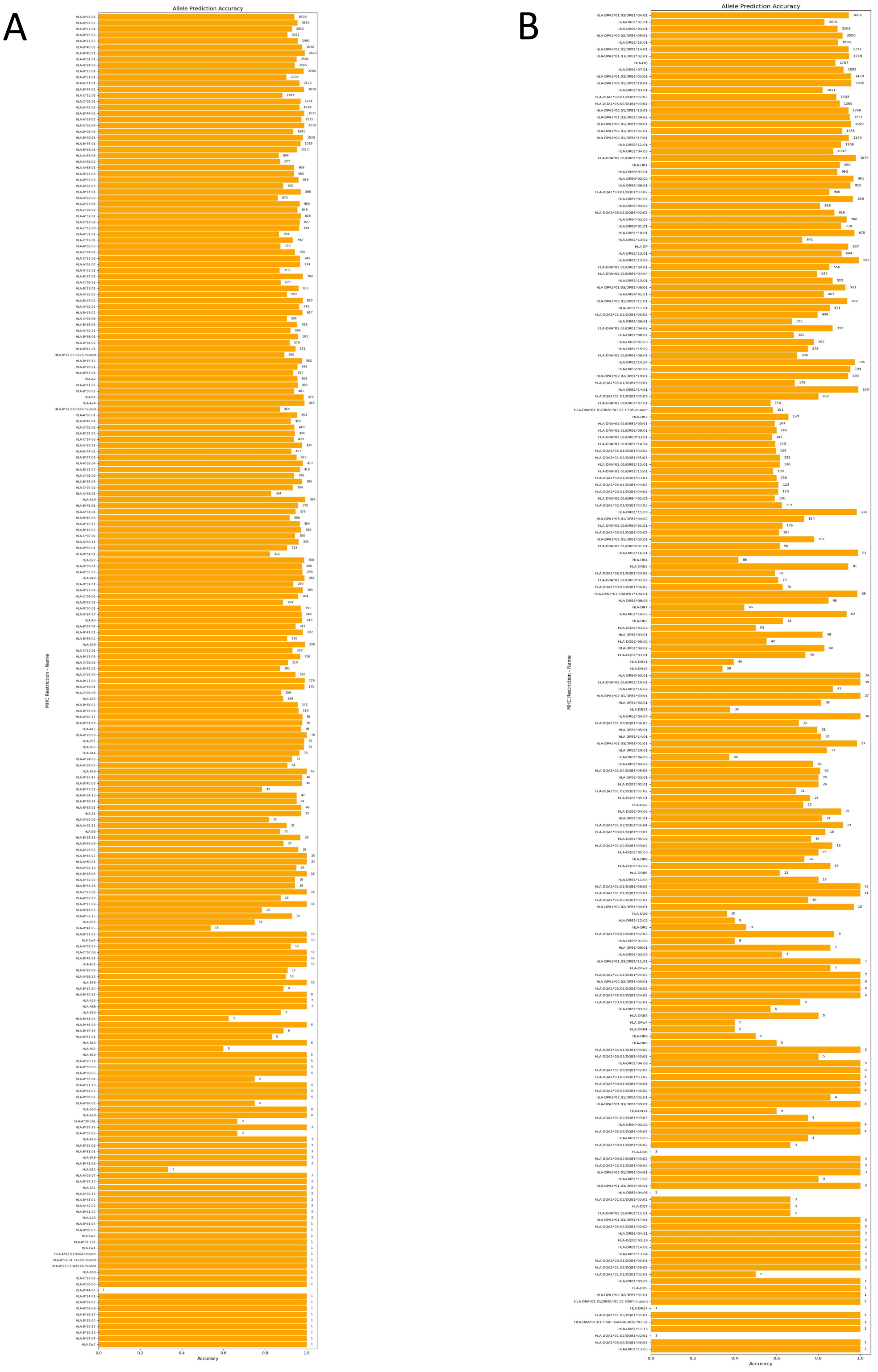

Supplementary Figure 6. This figure illustrates the prediction results of TransHLA on the test set for different HLA alleles. Panel A shows the results corresponding to HLA-I alleles, while Panel B displays those for HLA-II alleles. The number of epitopes corresponding to each allele is annotated to the right of each bar.

Feature	HLA I Importance	HLA II Importance
**Helix Content**	**0.315736**	**0.166237**
**Aromaticity**	**0.257780**	**0.218409**
**Flexibility**	**0.211219**	0.046253
Sheet Content	0.063786	0.091180
Isoelectric Point	0.043059	0.111911
Mean-Charge-at-PH-7	0.034550	0.089355
Coil Content	0.033767	0.048550
**Hydrophobicity**	0.016858	**0.129952**
Mean Molecular Weight	0.013744	0.052652
Instability Index	0.009502	0.045500

Supplementary Table 1: The importance of different Features in XGboost model

Type	ACC(%)	F1(%)	Recall(%)	MCC	Precision(%)	Specificity (%)
**HLA-I**	**84.75**	**67.73**	**80.06**	**0.59**	**58.70**	**86.11**
**HLA-I**	**71.14**	**45.08**	**59.24**	**0.28**	**36.39**	**74.12**

Supplementary Table 2: TransHLA's performance in Four-time Negative samples dataset

HLA I Type	Epitopes	Prediction Probability
**HLA I**	**ALWGFFPVL**	**0.9669**
	**LDTNADKQLSF**	**0.8441**
	**WQQGLRVSF**	**0.9774**
	**ILDTAGKEEY**	**0.9930**
**HLA II**	**PKYVKQNTLKLAT**	**0.9993**
	**ISTNIRQAGVQYSRA**	**0.9892**

Supplementary Table 3: TransHLA's performance of allele unseen epitopes in test dataset

HLA Restriction	Epitope
HLA-A*02:01 T163A mutant	ALWGFFPVL
HLA-A*02:01 W167A mutant	ALWGFFPVL
HLA-A*02:01 K66A mutant	ALWGFFPVL
HLA-Cw2	LDTNADKQLSF
HLA-B*15:04	WQQGLRVSF
HLA-A*01:191	ILDTAGKEEY
HLA-DRB1*13:04	PKYVKQNTLKLAT
HLA-DRB1*13:05	PKYVKQNTLKLAT
HLA-DRA*01:01/DRB1*01:01 G86Y mutant	PKYVKQNTLKLAT
HLA-DRB1*13:04	ISTNIRQAGVQYSRA

Supplementary Table 4: The unseen alleles epitopes in test data and their corresponding alleles

TransHLA Architecture Hyperparameters

The TransHLA model is specifically designed for applications in immunogenetics, focusing on the analysis of HLA (Human Leukocyte Antigen) sequences. It incorporates a hybrid architecture featuring both Transformer and Convolutional Neural Network (CNN) components. Below are detailed descriptions of the model components and their respective hyperparameters:

Model Components

- **Pretrained ESM-2 Model:** Uses the esm2_t33_650M_UR50D model for the initial embedding of sequences.
- **CNN Layers for Region and Structure Embedding:**
  - **Layer 1** (region_cnn1) and **Layer 2** (region_cnn2): Each serves different embedding purposes tailored to specific features of the input sequences.
- **Transformer Encoder:**
  - **Number of Layers** (n_layers): Determined by model-specific parameters, accommodating varying depths of processing.
  - **Number of Attention Heads** (n_head): Configured to optimize attention mechanisms across different parts of the input sequence.
  - **Model Dimension** (d_model): Defines the size of feature vectors within the transformer, influencing the capacity to handle information complexity.
  - **Feedforward Dimension** (d_ff): Specifies the size of the internal feed-forward networks, crucial for processing layers of features.
  - **Batch Normalization:** Implemented after certain layers to enhance model stability during training.
  - **Max Pooling and Padding:** Employed strategically within CNN layers to effectively reduce dimensionality and adjust tensor sizes.

Hyperparameters

- **Maximum Input Length** (max_len): 512 - Sets the limit on the length of sequences the model can process at once.
- **CNN Padding Index** (cnn_padding_index): 0 - Indicates the padding value used in CNN layers to maintain consistency in tensor dimensions.
- **CNN Number of Channels** (cnn_num_channel): 256 - Specifies the output channels of the CNN layers, directly influencing the breadth of feature detection.
- **Region Embedding Size** (region_embedding_size): Typically 3 filters that capture local contextual information within the sequence.
- **CNN Kernel Size** (cnn_kernel_size): 3 - Determines the extent of each convolution operation, affecting how the input data is processed.
- **CNN Padding Size** (cnn_padding_size): 1 - Ensures that the convolution output maintains appropriate dimensions.
- **CNN Stride** (cnn_stride): 1 - Controls the movement of the convolutional filters across the input field.
- **Pooling Size** (pooling_size): 2 - Defines the window size for max pooling, reducing spatial dimensions while preserving the most significant features.

Forward Pass Description

- **Input Processing:** Starts by embedding sequences using a pretrained ESM model, from which both sequence and structural representations are derived.

- **Transformer Encoding:** Advances the embeddings through a series of Transformer encoder layers, processing the complex dependencies within the data.
- **CNN Processing:** Applies a sequence of CNN layers to both sequence and structure-derived embeddings, enhancing feature extraction through residual connections and pooling techniques.
- **Feature Integration and Classification:**
    - Integrates outputs from both the Transformer and CNN pathways.
    - Propels the integrated features through dense layers for further refinement.
    - Concludes with a softmax layer to output classification probabilities, providing predictions on HLA types or related immunological responses.

This architecture is meticulously crafted to integrate intricate sequence and structural information, ensuring precise predictions in immunogenetics applications.

Deep Pyramid Convolutional Neural Network (DPCNN) Architecture Hyperparameters

The DPCNN model is designed for efficient text classification leveraging deep pyramid structures. Below we provide the hyperparameters used in the DPCNN architecture as described in the manuscript.

Embedding Layer:

- **Vocabulary Size (num_vocab):** 1000 words
- **Embedding Dimension (embedding_dim):** 256
- **Padding Index (padding_index):** 0 (zero-padding for alignment)
- Region Embedding:
- **Region Embedding Size:** Size of each region for initial feature extraction

Convolutional Layers:

- **Number of Channels (cnn_num_channel):** 256 channels for convolutional operations
- **Kernel Size (cnn_kernel_size):** 3 (width of the convolutional kernel)
- **Padding Size (cnn_padding_size):** 1 (padding applied to each side of the input)
- **Stride (cnn_stride):** 1 (stride of the convolutional operation)

Pooling Layer:

- **Pooling Size (pooling_size):** 2 (factor by which to downsample the input)

Output Layer:

- **Number of Classes (num_classes):** 2 (binary classification)

The model incorporates region embedding followed by two sets of convolutional operations with ReLU activation and constant padding. The output from the convolutional layers passes through max pooling before being flattened and passed to a fully connected layer for classification. The architecture employs residual connections and is optimized to handle varying lengths of text input with a focus on extracting hierarchical features for text representation.

Recurrent Neural Network with Attention (RNN_ATTs) Architecture Hyperparameters

The RNN_ATTs model incorporates an attention mechanism over a recurrent neural network for sequence modeling tasks. The following hyperparameters define the architecture:

- **Vocabulary Size:** 40
- **Embedding Dimension:** 256
- **Hidden Dimension (LSTM):** 128
- **Number of LSTM Layers:** 2
  Bidirectionality: Enabled (True)
  Dropout Rate: 0.2
- **Padding Index:** 0
- **Second Hidden Layer Size:** 64
- **Output Dimension:** 2 (binary classification)

The model uses an LSTM layer with tanh activation for sequence processing, followed by an attention mechanism that assigns weights to the LSTM outputs. The attention-weighted outputs are then passed through a fully connected layer with ReLU activation before reaching the final classification layer.

TextCNN Architecture Hyperparameters

The TextCNN model applies convolutional neural networks to text classification tasks. Here are the key hyperparameters:

- **Vocabulary Size:** 40 tokens
- **Embedding Dimension:** 128
- **Window Sizes:** [2, 4, 3] (corresponding to the size of the convolutional filters)
- **Maximum Sequence Length:** 21
- **Feature Size (Number of Convolutional Filters):** 256 per window size
- **Number of Classes (n_class):** 2 (binary classification)
- **Dropout Rate:** 0.4

The architecture consists of an embedding layer, followed by parallel convolutional layers with varying window sizes for feature extraction, and a max-pooling operation. The pooled features are concatenated and passed through a dropout layer before a fully connected layer for classification.

TextRCNN (Text Recurrent Convolutional Neural Network) Architecture Hyperparameters

The TextRCNN model combines recurrent neural network (RNN) with convolutional neural network (CNN) concepts for text classification. Here are the essential hyperparameters and components of the architecture:

- **Vocabulary Size:** 40 tokens
- **Embedding Dimension:** 128
- **Hidden Size (LSTM):** 50

- **Number of Labels (Output Dimension):** 2 (binary classification)
- **Dropout Rate:** 0.5

The architecture employs the following layers and operations:

- An embedding layer that maps tokens to vectors of the specified embedding dimension.
- A bidirectional LSTM layer for processing sequences, capturing both forward and backward context.
- A custom GlobalMaxPool1d layer that applies global max pooling over the time dimension of the LSTM output.
- Two linear layers with ReLU activation after the first linear transformation. The first linear layer expands the concatenated LSTM outputs and embedding vectors to a size of 256, and the second linear layer maps these to the final output dimension corresponding to the number of classes.

The forward pass of the model performs the following operations:

1. Embed the input sequence.
2. Process the embeddings through the LSTM layer to obtain the last hidden state.
3. Concatenate the embeddings with the LSTM's last hidden state.
4. Apply a linear transformation followed by a ReLU activation.
5. Perform global max pooling on the resulting tensor.
6. Apply dropout for regularization.
7. Pass the pooled features through the final linear layer to obtain the output classification scores.

Mhcnuggets Prediction Hyperparameters

```
from mhcnuggets.src.predict import predict
predict(class_='I',
            peptides_path=peptides_path,
            mhc='{}'.format(allele), output='{}.txt'.format(allele))
```

This code snippet uses the MHCnuggets library to predict peptide bindings to MHC molecules. Specifically:

1. Import the prediction function from MHCnuggets.
2. Call the predict function, specifying:

- class_='I' for predicting bindings to MHC class I molecules (use 'II' for MHC class II).
- peptides_path as the path to a text file containing peptide sequences.
- mhc, set to a specific allele name to use for prediction.
- output, set to the filename where the prediction results will be saved, named after the allele.
- Each allele is processed individually in a loop, with the prediction results for each saved to a separate file.

Mhcflurry Prediction Hyperparameters

```
import mhcflurry
predictor = mhcflurry.Class1PresentationPredictor.load()
results = predictor.predict(peptides, alleles=alleles_list)
```

This code snippet demonstrates the use of the MHCflurry library in Python to predict the binding of peptide sequences to MHC class I molecules. Here's a brief overview:

- Import the MHCflurry library.
- Load a class I presentation predictor.
- Use the predict method to predict the binding between a list of peptides (peptides) and a list of MHC class I alleles (alleles_list).

Due to a limitation in MHCflurry, it can only process up to 6 alleles at a time. Therefore, a loop is typically used to handle all alleles by processing them in batches of six.

NetMHCpan Prediction Hyperparameters

```
../netMHCpan -p NEPDB_I_peptide.pep -BA -xls -xlsfile ../HLA_I_result/output_HLA-A01109.csv -a allele
```

This code snippet demonstrates the use of the NetMHCpan to predict the binding of peptide sequences to MHC alleles. Here's a brief overview:

- -p means the input consists of peptides.
- -BA specifies that the prediction mode is BA (Binding Affinity), which is more robust.
- -xls -xlsfile means the output is in XLS format.
- output_path specifies the path for the output files.
- -a specifies the allele name.

MixMHCpred Prediction Hyperparameters

```
MixMHCpred -i INPUT_FILE -o OUTPUT_FILE [-a LIST_OF_ALLELES]  [-p PEPTIDES_SCORING] [-m OUTPUT_MOTIFS]
```

This code snippet demonstrates the use of the MixMHCpred to predict the binding of peptide sequences to MHC alleles. Here's a brief overview:

- -i, --input: Absolute or relative path to the input file (FASTA format or list of peptides).
- -o, --output: Name of the output file or directory.
- -p, --peptides_scoring: Enable (1) or disable (0) binding predictions. Default is 1 for peptides scoring, 0 for sequence alignment.
- -m, --output_motifs: Enable (1) or disable (0) plotting of logos and creation of an HTML file for motifs. Default is 0.
- -a, --alleles: List of MHC alleles separated by commas

Anthem Prediction Hyperparameters

```
python sware_b_main.py --HLA "$allele" --mode prediction --peptide_file "$peptide_file"
```

This code snippet demonstrates the use of the Anthem to predict the binding of peptide sequences to MHC alleles. Here's a brief overview:

- --peptide_file: the path of the file that contains peptide sequence in text format or protein sequence in fasta format, respectively.
- --HLA: List of MHC alleles separated by commas
- --mode: prediction the mode choose to use. In train model function, if users want to use their trained model, the mode is "useYourOwnModel"

TransPHLA Prediction Hyperparameters

```
python pHLAIformer.py --peptide_file "peptide.fasta" --HLA_file "./HLA/HLA_allele.fasta" --threshold 0.5
--cut_peptide False --cut_length 14 --output_dir "./results/" --output_attention False --output_heatmap False
--output_mutation False
```

This code snippet demonstrates the use of the TransPHLA to predict the binding of peptide sequences to MHC alleles. Here's a brief overview:

- peptide_file: type = str, help = the filename of the .fasta file contains peptides
- HLA_file: type = str, help = the filename of the .fasta file contains sequence
- threshold: type = float, default = 0.5, help = the threshold to define predicted binder, float from 0 - 1, the recommended value is 0.5
- cut_peptide: type = bool, default = True, help = Whether to split peptides larger than cut_length?
- cut_length: type = int, default = 9, help = if there is a peptide sequence length > 15, we will segment the peptide according the length you choose, from 8 - 15
- output_dir: type = str, help = The directory where the output results are stored.
- output_attention, type = bool, default = True, help = Output the mutual influence of peptide and HLA on the binding?
- output_heatmap: type = bool, default = True, help = Visualize the mutual influence of peptide and HLA on the binding?
- output_mutation: type = bool, default = True, help = Whether to perform mutations with better affinity for each sample?

DeepSeqPanII Prediction Hyperparameters

We Match Alleles and Models: Ensure that each allele is matched with the appropriate model, And process sequences: Process all required sequences using the specified model and alleles.

To use DeepSeqPanII with similar parameters, you can follow these steps:

- Clone DeepSeqPanII: Clone the DeepSeqPanII repository into the desired folder with the command:
- git clone https://github.com/pcpLiu/DeepSeqPanII.git
- Switch Directory: After cloning, navigate to the code_and_dataset directory:
- cd DeepSeqPanII/code_and_dataset
- Use the Command: Execute the following command to run DeepSeqPanII:

```
python deepseqpanII.py <model_path> <allele1_name> <allele2_name> <peptide_sequence>
```

This code snippet demonstrates the use of the DeepSeqPanII to predict the binding of peptide sequences to MHC alleles. Here's a brief overview:

- <model_path> is the path where the model is located.
- <allele1_name> is the name of the first allele.
- <allele2_name> is the name of the second allele.
- <peptide_sequence> is the peptide sequence to be processed.

NetMHCIIpan Prediction Hyperparameters

```
./netMHCIIpan -inptype 1 -f NEPDB.pep -BA -xls -xlsfile  output_path -a allele
```

This code snippet demonstrates the use of the NetMHCIIpan to predict the binding of peptide sequences to MHC alleles. Here's a brief overview:

- -inptype 1 means the input consists of peptides.
- -BA specifies that the prediction mode is BA (Binding Affinity), which is more robust.
- -xls -xlsfile means the output is in XLS format.
- output_path specifies the path for the output files.
- -a specifies the allele name.

MixMHC2pred Prediction Hyperparameters

```
MixMHCpred -i INPUT_FILE -o OUTPUT_FILE [-a LIST_OF_ALLELES]  [-p PEPTIDES_SCORING] [-m OUTPUT_MOTIFS]
```

This code snippet demonstrates the use of the MixMHC2pred to predict the binding of peptide sequences to MHC alleles. Here's a brief overview:

- -i, --input: Absolute or relative path to the input file (FASTA format or list of peptides).
- -o, --output: Name of the output file or directory.
- -a, --alleles: List of MHC alleles separated by commas
- --no_context: Use the --no_context option in MixMHC2pred for analyzing pre-cleaved peptides without context, which is suitable for experiments testing specific peptides directly.

Manuscript

Click here to access/download;Manuscript;TransHLA.pdf

GigaScience, 2023, 1–14

doi: xx.xxxx/xxxx
Manuscript in Preparation
Paper

PAPER

TransHLA: A Hybrid Transformer Model for HLA-Presented Epitope Detection

Tianchi LU[1,†], Xueying Wang[1,2,†], Wan Nie[1], Miaozhe Huo[1] and Shuaicheng LI[1,*]

[1]Department of Computer Science, City University of Hong Kong, Kowloon, Hong Kong and [2]Department of Computer Science, City University of Hong Kong (Dongguan), Dongguan523000, China

[*]To whom correspondence should be addressed. Tel: +852 3442-9412; Email: shuaicli@cityu.edu.hk
[†]Contributed equally.

Abstract

Background: Precise prediction of epitope presentation on human leukocyte antigen (HLA) molecules is crucial for advancing vaccine development and immunotherapy. Conventional HLA-peptide binding affinity prediction tools often focus on specific alleles and lack a universal approach for comprehensive HLA site analysis. This limitation hinders efficient filtering of invalid peptide segments.
Results: We introduce TransHLA, a pioneering tool designed for epitope prediction across all HLA alleles, integrating Transformer and Residue CNN architectures. TransHLA utilizes the ESM2 large language model for sequence and structure embeddings, achieving high predictive accuracy. For HLA class I, it reaches an accuracy of 84.72% and an AUC of 91.95% on IEDB test data. For HLA class II, it achieves 79.94% accuracy and an AUC of 88.14%. Our case studies using datasets like CEDAR and VDJdb demonstrate that TransHLA surpasses existing models in specificity and sensitivity for identifying immunogenic epitopes and neoepitopes.
Conclusions: TransHLA significantly enhances vaccine design and immunotherapy by efficiently identifying broadly reactive peptides. Our resources, including data and code, are publicly accessible at `https://github.com/SkywalkerLuke/TransHLA`

Key words: Epitope Presentation, Pre-trained language model, Deep Learning

Introduction

The intricate process of epitope presentation by human leukocyte antigen (HLA) molecules is a cornerstone of the immune system's ability to combat pathogens, neoplasms, and its involvement in the multifaceted arenas of autoimmunity, allergies, and organ transplant rejection [1, 2]. HLA class I and II molecules play a pivotal role in presenting crucial antigen peptides to T cells[3], thereby triggering downstream immune responses.

Due to the extensive polymorphism of HLA molecules, their affinity for a wide range of peptides can vary significantly, posing a challenge for vaccine design in accurately identifying peptides that can bind to HLAs[4, 5, 6]. The burgeoning interest in HLA peptide binding has revealed the presentation of antigenic peptides by over 22,000 HLA alleles. This wealth of information constitutes a substantial database for deep learning models, offering ample resources for their development and training[7].

There are two categories of models used to predict the binding affinity between peptides and HLA alleles. The first category includes models trained individually for specific alleles, requiring users to input a peptide and select a specific allele. Examples of this category are MHCnuggets [4] and Anthem[8]. As these models assume that peptides binding to the same allele share similar characteristics, they are dependent on the alleles present in the training data, thus exhibiting limited generalization performance when applied to extensive peptides compared to the second category. The second category, known as pan-allele models, does not strictly limit predictions to specific HLA alleles. Instead, they take both the epitope and HLA allele sequences as input, as seen in models like MHCflurry [5], NetMHCpan(RRID: SCR_006604) [9], NetMHCIIpan [9], DeepSeqpanII [6], TransPHLA [10], MixMHCpred [11], and MixMHC2pred [12]. This combined embedding approach al-

Compiled on: December 23, 2024.
Draft manuscript prepared by the author.

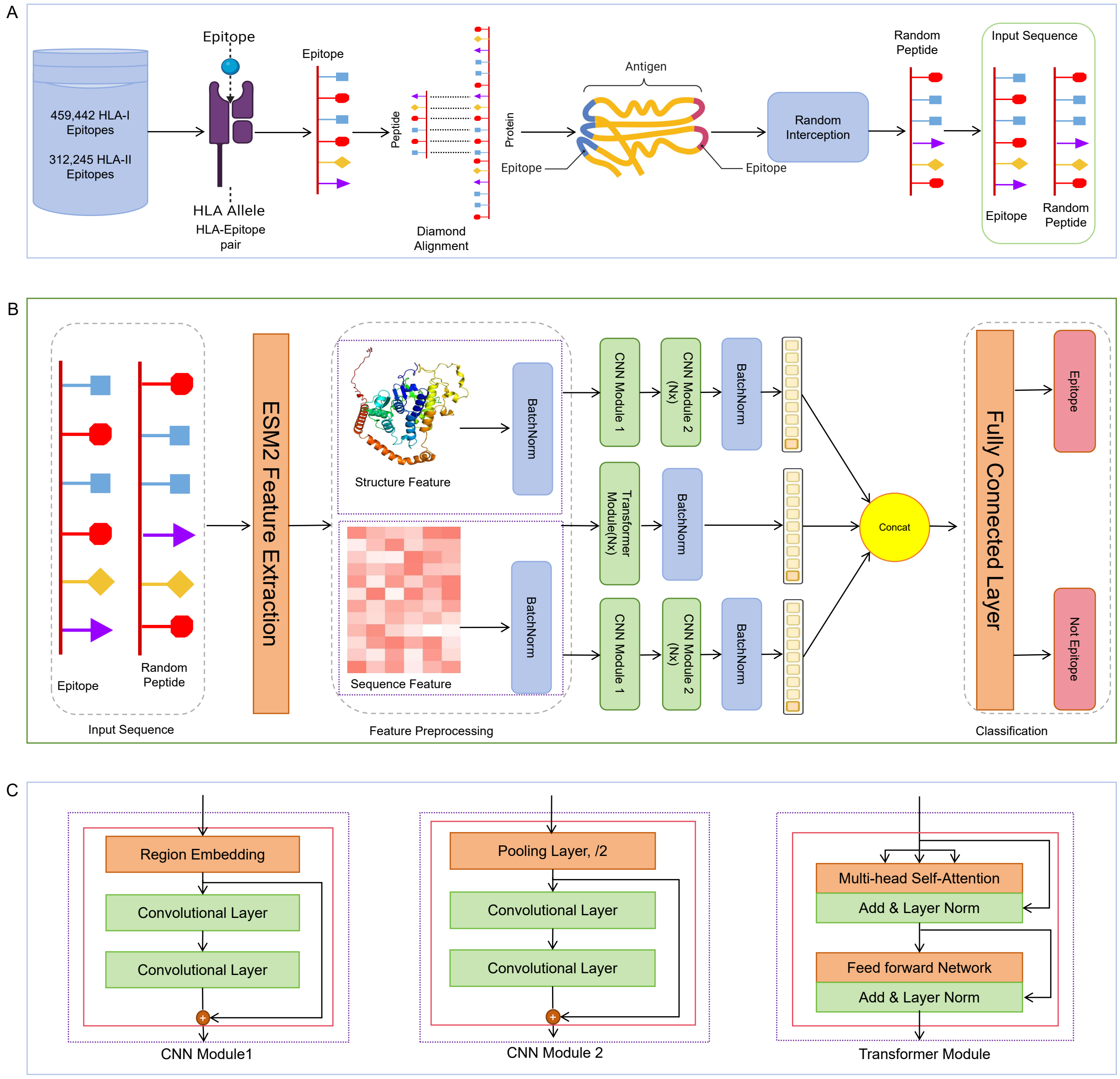

Figure 1. Overview of data acquisition and predictive modeling using TransHLA. (A) Data Acquisition: The dataset, derived from the IEDB, features a variety of peptide sequences that bind to HLA class I or II molecules. For negative sample generation, Non-overlapping random peptide fragments were sourced by matching positive peptides to their originating proteins through sequence alignment. Then, the Non-overlapping random peptide fragments were processed with CD-HIT to achieve a reduced redundancy, resulting in the final set of negative samples. (B)With ESM2's advanced modeling capabilities, we generated high-dimensional sequence embeddings for the peptides associated with both HLA classes. Concurrently, structural insights were obtained through ESM2's contact map predictions, yielding structure embeddings. These two distinct yet complementary sets of embeddings were crafted to capture the intricate nature of peptide-HLA interactions. describes the process from data input to epitope presentation prediction. (C) The architecture of the different modules, including the CNN Module 1, CNN Module 2 and the Transformer module.

Key Points

- We developed TransHLA, a deep learning tool for predicting epitopes across all HLA alleles using Transformer and Residue CNN architectures.
- The model uses ESM2 embeddings to improve predictive accuracy and efficiency.
- TransHLA shows superior specificity and sensitivity in identifying immunogenic epitopes and neoepitopes compared to existing models.
- Our approach offers potential advancements in vaccine design and immunotherapy through enhanced peptide analysis.

lows for a richer feature set, enhancing generalization performance. Both categories require simultaneous input of peptides and alleles to identify potential epitopes

However, while these methods can accurately determine the affinity between specific HLA residues and peptides, they rely heavily on the selection of HLA sites and lack a universal approach that focuses solely on peptides to efficiently filter out invalid peptide segments. To overcome this issue, our TransHLA does not predict binding affinity but instead predicts whether a peptide is a potential presented epitope. TransHLA only requires peptides as input and is built upon a combination of Transformer [13] and Residual CNN(Convolutional Neural Networks) architectures [14], leveraging both the sequence and structural attributes of peptides to assess the HLA binding potential. To improve feature extraction, we utilized a pretrained protein language model called Evolutionary Scale Modeling 2 (ESM2) [15], which employs an autoencoder architecture to derive a semantic and structural representation of the sequence. We have selected several state-of-the-art sequence classification models, namely TextCNN [16], TextRCNN [17], DPCNN [18], and RNN-ATTs [19], for benchmarking and comparison purposes. In addition, in the case study, we employed state-of-the-art peptide-HLA allele binding prediction software to perform predictions for all alleles. We then compared these predictions with the results obtained from TransHLA. For HLA I molecules, TransHLA achieves accuracy of 84.72% and AUC of 91.95% in IEDB test data; a 0.97% improvement in accuracy over the second-place method. For HLA II molecules, TransHLA achieves accuracy of 79.94% with an AUC of 88.14% in IEDB test data; a 2.53% improvement in accuracy over the second-place method. The comprehensive analysis of the results consistently demonstrated that TransHLA outperformed the other models in general epitope prediction for both HLA-I binding and HLA-II binding.

Materials and methods

Datasets

Data Collection

The datasets used in this work were collected and curated from IEDB(RRID: SCR_006604) [20], CEDAR [21], VDJdb [22], ImmuneCode [23], dbPepNeo2.0 [24], and NEPDB [25] databases.

The filtering criteria for each database are as follows:

IEDB: "MHC Ligand", "Linear Peptide", "Host" as Human, and "Outcome" as Positive.

CEDAR: "MHC Ligand", "Linear Peptide", "Host" as Human, and "Outcome" as Positive.

VDJdb: "Species" as Human.

ImmuneCode: We download the raw data, and add it into the analysis data.

dbPepNeo2.0: HC neoantigens.

NEPDB: "response" as positive samples.

We also removed peptides containing special characters.

Train-Validation-Test Data Construction

The IEDB database provided the source of our train, validation, test. The other four databases—CEDAR, VDJdb, ImmuneCode, and dbPepNeo2.0 were utilized exclusively for external test to assess the generalizability of our models. The NEPDB were utilized for assessing the performance on the neoepitope prediction. Our particular emphasis was on epitopes originating from human hosts that exhibited a positive outcome in Ligand elution/Mass spectrometry assays. For epitopes presented by HLA-II, the peptide length varied between 13 and 21 amino acids [9], whereas for epitopes presented by HLA-I, the peptide length was within the range of 8 to 14 amino acids [9].

To construct the train-validation-test datasets for both HLA-II and HLA-I, the positive samples were collected from IEDB [20] using the filtering criteria "MHC Ligand", "Linear Peptide", "Host" as Human, and "Outcome" as Positive. For negative samples, we used diamond [26] to blast the positive peptide sequences against the non-redundant (nr) database [27], recovering the proteins from which the sequences originated. From these proteins, random fragments excluding the positive sequences were selected to ensure non-overlap. For the positive samples, we removed peptides that did not align with any protein in the nr database using diamond.

In this way, we obtained negative samples that are representative of the potential peptide repertoire but do not include the known positive epitopes. Sequence redundancy was removed using CD-HIT [28] with a threshold of 0.8. Finally, we obtained balanced datasets for both HLA-II and HLA-I as follows: 312,245 positive samples and an equal number of negative samples for epitopes presented by HLA-II, and 459,442 positive and negative samples for epitopes presented by HLA-I. The datasets were divided into train, validation, and test sets in a ratio of 7:1:2. The details can be found in Table 1.

To concurrently ascertain the reasonableness of our data distribution, we examined the frequencies of HLA alleles associated with peptide binding within the IEDB database. The frequency distribution of HLA alleles demonstrates highly similar characteristics across the training, validation, and test datasets (Figure 2 and Supplementary Figure 5). Our train-validation-test data includes 258 different HLA-I alleles and 227 different HLA-II alleles. Additionally, due to the larger number of HLA-A types, we plotted the top 50 alleles, while for HLA-B and HLA-C, all alleles were included. In the Supplementary Figure 5, we also plotted the top 50 alleles for HLA-DR, HLA-DQ, and HLA-DP.

Table 1. The number of samples on training datasets and independent test datasets

Datasets	Types	Count
HLA-I Epitopes	Positive	459,442
	Negative	459,442
HLA-II Epitopes	Positive	312,245
	Negative	312,245

Pre-trained Embeddings for Sequence and Structure

Pre-trained protein language models[29, 15, 30] have been extensively applied in various tasks, such as protein classification, by providing intricate representations of protein sequences [31, 32, 33]. Additionally, AlphaFold2 [34] and ColabFold [35] have set high standards in protein structure prediction.

In our approach, we address the issue of HLA-I binding epitopes with lengths less than 14 and HLA-II binding epitopes with

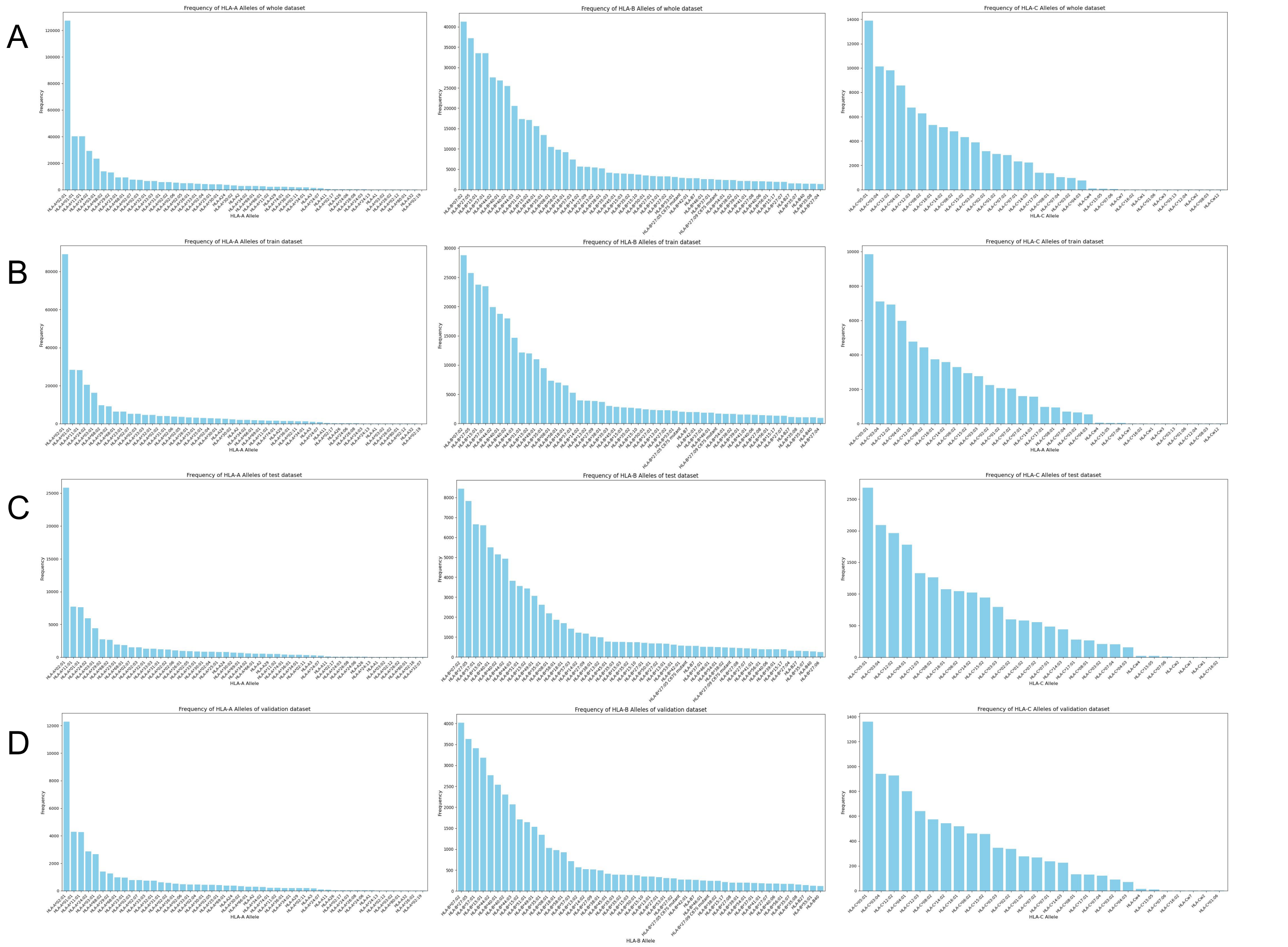

Figure 2. This figure illustrates the experimental distribution of major HLA-I alleles in the IEDB. We plotted the alleles by type, where subfigure A shows the distribution for all data, B for the training dataset, C for the test dataset, and D for the validation dataset.Left represents HLA-A alleles, middle represents HLA-B alleles, and right represents HLA-C alleles.

lengths less than 21 by padding the end with ones. This padding technique ensures that the sequences have the required lengths. Subsequently, we utilize the ESM2 protein language model to extract sequence embeddings and predict structure embeddings for these epitopes[15].

The Architectures of the Deep Learning Model

The Transformer module

To enhance the extraction of global features, we incorporated the Transformer encoder module [13, 36], which utilizes inputs in the form of pre-trained sequence features extracted by ESM2, represented as a $\mathbf{E} \in R^{1280 \times peptide\_length}$ matrix. The module leverages a multi-head attention mechanism to facilitate effective global feature extraction.

Within each attention head, three key components are involved: Q (query), K (key), and V (value). Q represents the current position being attended, while the K and V represent other positions in the peptide sequence. By computing the attention weights between the Q and K, the model determines the importance of each position and assigns higher weights to more relevant positions. The values are then combined based on these attention weights to generate the output representation, and the scaled dot-product attention is calculated as:

$$\text{Attention}(Q, K, V) = \text{softmax}\left(\frac{QK^T}{\sqrt{d_k}}\right)V \tag{1}$$

d_k is the dimension of the key vectors.

The multi-head attention is achieved through a series of operations to transform the input vectors Q, K, and V for h times (where h is the number of heads). Each transformed vector undergoes scaled dot-product attention independently. Finally, the attention outputs are concatenated and further transformed. This process can be expressed as follows:

$$\text{MultiHead}(Q, K, V) = \text{Concat}(\text{head}_1, \ldots, \text{head}_h)W_O$$

$$\text{where} \quad \text{head}_j = \text{Attention}(QW_{Q_j}, KW_{K_j}, VW_{V_j}) (2)$$

In the given equation, W_{Q_j}, W_{K_j}, and W_{V_j} represent weight matrices for each head corresponding to the Q, K, and V vectors, respectively. W_O denotes the weight matrix for the output. This formulation allows for weight sharing across heads, reducing redundancy and promoting a more compact representation.

The CNN module

To enhance feature extractions, TransHLAs employs two structurally identical CNNs, each consisting of a CNN module 1 for region embedding, followed by multiple layers of CNN module 2. These

modules process both the pre-trained sequence features $\mathbf{E}$ and the contact map structural features extracted by ESM2, with the contact map being a symmetric matrix $\mathbf{S} \in R^{peptide\_length \times peptide\_length}$. Residual connections are implemented between each module to prevent gradient vanishing and ensure effective training of the deep network structure.

CNN Module 1.
It first applies a text region embedding layer to get a dense representation of sequences:

$$\mathbf{x} = \text{RegionEmbed}(\mathbf{E}) \tag{3}$$

where $\mathbf{x} \in R^{M \times D}$ and M is the number of text regions and D is the region embedding dimension.

This is followed by a convolution block, which contains two convolutional layers each with 256 feature maps:

$$\mathbf{z}_1 = \text{ConvBlock}(\mathbf{x}) \tag{4}$$

where $\mathbf{z}_1 \in R^{M \times 256}$.

CNN Module 2.
Following the CNN module 1, the CNN module 2 commences with a pooling layer that reduces the length of the feature map to half of its original size. Subsequently, CNN module 2 employs a convolutional block with the same structure as the one in CNN module 1, featuring an isometric convolutional layer with 256 channels.

$\mathbf{z}_1$:

$$\mathbf{x}_1 = \text{Downsample}(\mathbf{z}_1) \tag{5}$$

where $\mathbf{x}_1 \in R^{M/2 \times 256}$.

$$\mathbf{z}_2 = \text{ConvBlock}(\mathbf{x}_1) \tag{6}$$

where $\mathbf{z}_2 \in R^{M/2 \times 256}$.

This is repeated for L times, with the downsampling layer and l-th CNN Modules being:

$$\mathbf{x}_l = \text{Downsample}(\mathbf{z}_l) \tag{7}$$
$$\mathbf{z}_{l+1} = \text{ConvBlock}(\mathbf{x}_l) \tag{8}$$

where $\mathbf{z}_{l+1} \in R^{M/2^l \times 256}$.

After the final Downsample layer, we add a batchnorm layer, which helps reduce internal covariate shift and acts as a regularization technique.

$$\mathbf{z}_{l+1} = \text{BatchNorm}(\mathbf{z}_{l+1}) \tag{9}$$

The TIM Loss
The TransHLA framework is developed based on a modified Transductive Information Maximization (TIM) loss function, as introduced by Boudiaf et al. (2020) [37], which merges conventional cross-entropy with a mutual information component,tailored for empirical analysis. We address the empirical mutual information within dataset X (comprising amino acid sequences) linked to their respective outcomes Y (which now signify epitope presentation). The first factor is the empirical conditional entropy of the outcomes given the data, denoted as $\widehat{\mathcal{H}}(Y|X)$, which reduces uncertainty in predictions for unlabeled samples, encouraging the model to output confident predictions. The next factor is the empirical marginal entropy of the outcomes, denoted as $\widehat{\mathcal{H}}(Y)$, which encourages a uniform distribution of labels, preventing bias towards any particular class. To further calibrate the binary classification process, the cross-entropy loss, indicated as CE, between the model's predictions and the actual outcomes is incorporated.By integrating these entropy terms, the TIM loss enhances the model's generalization in few-shot scenarios without the need for complex meta-learning schemes. The formulation for these components is defined as follows

$$\begin{aligned} \widehat{\mathcal{H}}(Y) &:= -\sum_{k=1}^{K} \widehat{p}_k \log \widehat{p}_k \\ \widehat{\mathcal{H}}(Y \mid X) &:= -\frac{1}{|X|} \sum_{i \in X} \sum_{k=1}^{K} p_{ik} \log(p_{ik}) \\ \text{CE} &:= -\frac{1}{|X|} \sum_{i \in X} \sum_{k=1}^{K} y_{ik} \log(p_{ik}) \end{aligned} \tag{10}$$

Let $|X|$ denote the total count of sequences within the dataset, with i being the sequence identifier in X, and K representing the possible outcome categories. The variable p_{ik} is the predicted likelihood of the i-th amino acid sequence being classified within the k-th category. The binary variable y_{ik} is used to indicate if the i-th sequence is actually categorized under class k. We assign $K = 2$ for this model since the task at hand is a binary classification problem.

The final loss function for TransHLA is defined as:

$$\widehat{\mathcal{L}}(X;Y) := \text{CE} - \widehat{\mathcal{H}}(Y) + \alpha \widehat{\mathcal{H}}(Y \mid X) \tag{11}$$

Where α is hyperparameter that determine the rate of convergence for each term in the loss function. In experiments, we set $\alpha = 0.04$, considering the standard cross-entropy loss and standard mutual information.

By selecting these particular hyperparameter values, we maintain fairness and impartiality in TransHLA's training process.

Performance evaluation

In the Benchmark Results with other sequence classification models, we employed the aforementioned metrics, including accuracy (ACC), Recall, F1-score (F1), and Matthews Correlation Coefficient (MCC), to evaluate the performance of TransHLA.

$$ACC = \frac{TP + TN}{TP + FP + TN + FN} \tag{12}$$

$$Recall = \frac{TP}{TP + FN} \tag{13}$$

$$F1 = \frac{2TP}{2TP + FN + FP} \tag{14}$$

$$MCC = \frac{TP \times TN - FP \times FN}{\sqrt{(TP + FP) \times (TP + FN) \times (TN + FP) \times (TN + FN)}} \tag{15}$$

Additionally, in the Comparison Results with other HLA-epitope binding software across extensive datasets, we augmented our assessment with two further metrics, Precision and Specificity. These additional indicators highlight how our methodology has overcome the limitations commonly associated with traditional affinity-binding software, particularly the tendency to incorrectly

classify negative samples as positive.

$$Precision = \frac{TP}{TP + FP} \quad (16)$$

$$Specificity = \frac{TN}{TN + FP} \quad (17)$$

Where TP (true positives) represents the number of correctly identified true epitope, TN (true negatives) represents the number of correctly identified the normal peptide, The FP (false positives) represents the number of instances where normal peptides were incorrectly identified as epitopes , and FN (false negatives) represents the number of instances where epitopes were incorrectly identified as normal peptides.

In addition to these metrics, we also utilize Receiver Operating Characteristic (ROC) and Precision-Recall (PR) curves as significant evaluation tools for classification accuracy. The Area Under the ROC Curve (AU-ROC) and the Area Under the Precision-Recall Curve (AU-PRC) values quantify the overall performance by measuring the area beneath the ROC and PR curves, respectively.

Results

Benchmark Results with other sequence classification models

Since this paper focuses on the epitope presentation classification problem, a corresponding software for comparison is not yet established. We conducted comparison experiments on independent test sequences from IEDB including 92,347 HLA-I binding epitopes, 65,105 HLA-II binding epitopes and 157,879 random sequences with the state-of-the-art sequence classification models, including TextCNN [16], TextRCNN [17], DPCNN [18], and RNN-ATTs [19].

The performance metrics are presented in Table 2. TransHLA exhibits enhanced performance in classifying HLA-I epitopes across five metrics, including ACC, F1, Recall, MCC and AUC(AU-ROC). The corresponding values for TransHLA are 0.847, 0.846, 0.694, and 0.920, respectively. In comparison, the second-ranked software obtains scores of 0.838, 0.838, 0.68 and 0.910 for the same metrics, respectively. Figure 3A and 3C illustrate the ROC and PR curves of the compared models.

HLA-II binding predictions are known to be more complex compared to HLA-I predictions. [38, 39, 40, 12] Consequently, the performance of the models, in general, is inferior in terms of HLA-II binding metrics of other models, with an average decrease of 9.3% in ACC, 7.26% in F1, 11.73% in Recall, 0.19 in MCC, 9.00% in AUC. However, even in the challenging task of HLA-II binding prediction, TransHLA demonstrates robust classification performance. Compared to the values achieved in HLA-I binding prediction, TransHLA only experiences a decrease of 4.78%in ACC, 3.52% in F1, 0.09 in MCC, and 3.81% in AUC. Remarkably, TransHLA achieves an increase of 2.27% in Recall. Furthermore, in the prediction of HLA-II epitope binding, TransHLA demonstrates superior performance across all evaluation metrics. Compared to the next best-performing models, TransHLA achieves an improvement of 0.25 in ACC, an enhancement of 0.316 in the F1 Score, a boost of 1.02 in Recall, and a substantial improvement of 0.548 in MCC.

The prediction performance in different alleles

To further discuss TransHLA's performance across different alleles, we calculated the positive sample prediction accuracy, or recall, for alleles sorted by the number of corresponding epitopes from high to low. The detailed results are shown in Supplementary Figure 6. Overall, when frequencies are higher, prediction performance tends to improve. For example, in HLA class I, when the frequency is above 42, the average performance is around 80%, some achieves 90%,such as HLA-B*40:01, HLA-B*44:03. However, when the frequency is below 42, some alleles show fluctuating results, with accuracy for certain alleles dropping to below 55%, such as HLA-B41:05, HLA-B41:04, and HLA-B15, leading to lower overall effectiveness compared to class II. In HLA class II, when frequencies exceed 200, the overall accuracy is higher, about 80% or more. For frequencies between 100 and 200, such as HLA-DRA01:01/DRB107:01 to HLA-DQA102:01/DQB03:03, performance is slightly lower, with accuracy around 50-65%. However, even alleles with fewer epitopes can still achieve relatively high accuracy. In class I, examples include HLA-B41:05, HLA-B41:04, and HLA-B15, while in class II, examples are HLA-DRB*03:01 and some alleles with even lower frequencies.

Meanwhile, due to the random allocation of epitopes to train-validation-test sets, some test epitopes' alleles did not appear in the train and validation sets. These data were also included in our analysis. For class I, epitopes such as 'ALWGFFPVL', 'LDTNADKQLSF', 'WQQGLRVSF', 'ILDTAGKEEY' were included. For class II, epitopes like 'PKYVKQNTLKLAT' and 'ISTNIRQAGVQYSRA' were analyzed. We found that our model predicted these peptides as positive samples with probabilities greater than 0.8. The specific prediction results are in Supplementary Table 3, and the corresponding alleles are listed in Supplementary Table 4.

Comparison Results with other HLA-epitope binding software in the case study

We employed our software along with various state-of-the-art epitope-HLA binding prediction tools, including Mhcflurry [5], NetMHCpan4.1b [9], NetMHCIIpan4.3b [9], TransPHLA [10], Anthem[8], MixMHCpred [11], DeepSeqPanII [6], MixMHCIIpred [12] and Mhcnuggets [4] to evaluate their accuracy in correctly identifying sequence as potential epitopes from CEDAR [21], VDJdb [22], ImmuneCode [23], and dbPepNeo2.0 [24] datasets. Our analysis yielded a total of 21,387 HLA-I binding epitopes and 3,580 HLA-II binding epitopes in the case study. Our criterion for deeming a peptide as a presentation-worthy epitope is that it must exhibit binding affinity to at least one major HLA allele. Mhcflurry contains 11,576 HLA-I alleles, Mhcnuggets contains 118 HLA-II alleles and 106 HLA-I alleles, and DeepSeqPanII contains 61 HLA-II alleles. And the details information of alleles used in each tool can be found in our Data availability.

In this case study, we added random sequences in the same quantity as the identified persistent epitopes. The results of the experiments are presented in Table 3. For the prediction of HLA-I binding, we compared the performance of the TransHLA against Mhcflurry, NetMHCpan, MixMHCpred, TransPHLA, Anthem, and Mhcnuggets. In the prediction of HLA-II binding, we compared the performance of the TransHLA, DeepSeqPanII, Mhcnuggets, MixMHC2pred, NetMHCIIpan, and MixMHC2pred. The details of the parameters used in the mentioned methods can be found in the Supplementary File. TransHLA, Mhcflurry, and NetMHCpan demonstrated good performance in predicting HLA-I binding. Among the seven models, TransHLA achieved the highest ACC of 83.09%, precision of 85.22%, and specificity of 86.11%, followed by Mhcflurry with 82.96%, 81.95%, and 81.38% in the same metrics. Besides, TransHLA, in conjunction with the Mhcflurry and NetMHCpan, obtained the highest MCC of 0.66 in this experiment. Mhcflurry had the highest F1-score of 83.24%, while TransHLA obtained the second highest F1-score of 82.96%. Upon closer examination of the F1 score metrics, it was observed that TransHLA attained the highest true negative (TN) rate of 86.1%, which surpassed that of MhcFlurry and NetMHCpan at 81.4% and 76.2%. Conversely, NetMHCpan exhibited the highest true positive (TP) rate at 86.0%. This differential performance indicates that TransHLA

Table 2. Benchmark Results with other sequence classification models

Type	Method	ACC (%)	F1(%)	Recall(%)	MCC	AUC (%)
HLA-I	TransHLA	**84.72**	**84.59**	83.92	**0.69**	**91.95**
	TextCNN	81.63	79.61	83.50	0.63	89.37
	TextRCNN	81.21	76.65	83.78	0.64	87.62
	DPCNN	83.75	83.76	**83.96**	0.68	90.97
	RNN-ATTs	81.17	82.27	81.46	0.63	87.98
HLA-II	TransHLA	**79.94**	**81.07**	**86.19**	**0.60**	**88.14**
	TextCNN	73.26	73.49	72.64	0.47	80.64
	TextRCNN	70.96	72.02	69.28	0.42	78.21
	DPCNN	77.41	77.91	75.98	0.55	85.30
	RNN-ATTs	69.04	69.83	67.89	0.38	75.81

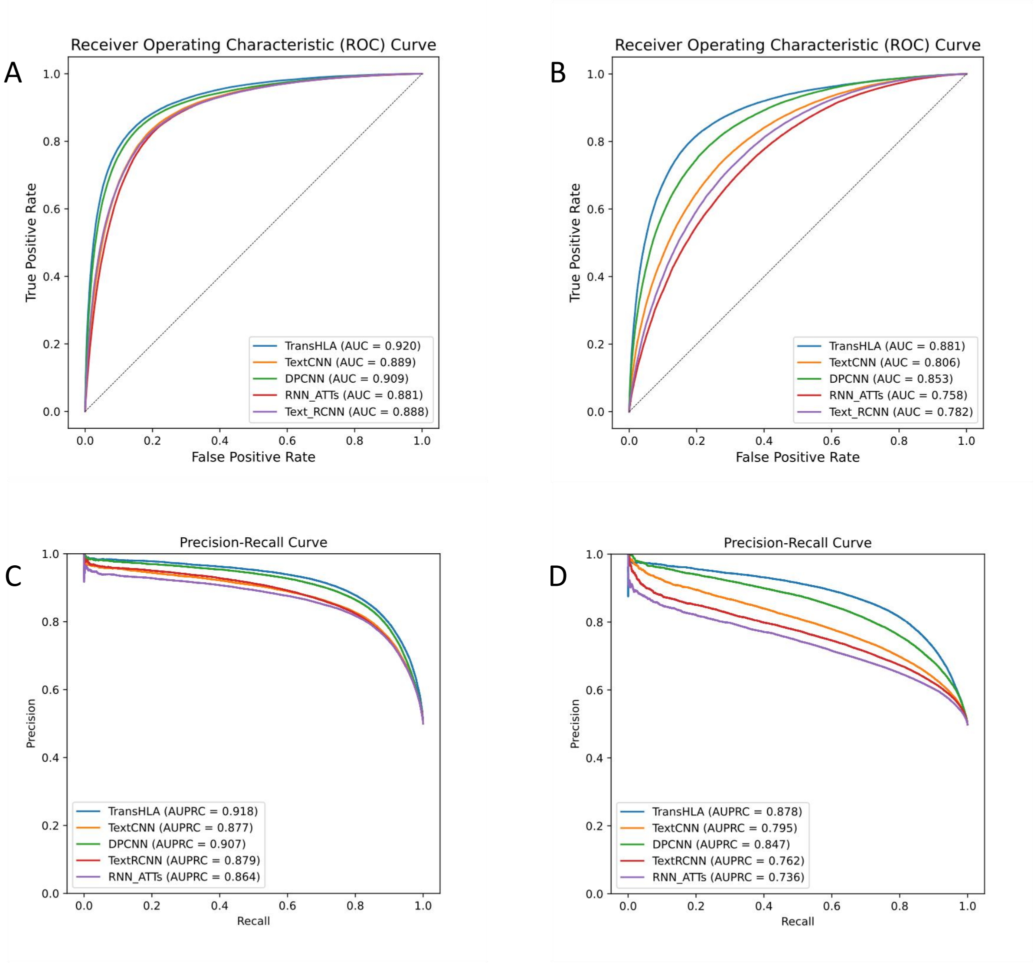

Figure 3. This figure evaluates TransHLA's epitope prediction capabilities, benchmarked against other models using two key statistical metrics: AUROC and AUPRC. Subfigures (A) and (C) examine HLA-I class performance, with (A) showing AUROC and (C) presenting AUPRC. The graphs demonstrate TransHLA's proficiency in distinguishing between epitope and non-epitope peptides for HLA-I, where AUROC indicates its discriminative power and AUPRC reflects the precision-recall trade-off. Subfigures (B) and (D) extend the analysis to HLA-II, with (B) displaying AUROC and (D) illustrating AUPRC. The performance depicted emphasizes TransHLA's effectiveness in identifying HLA-II epitopes, highlighting its ability to differentiate between classes under imbalanced distributions. Together, the subfigures demonstrate TransHLA's advantage over conventional models in epitope prediction for both HLA classes.

Table 3. Comparison Results with other HLA-epitope binding softwares in External Epitope Datasets Case Study

Type	Method	ACC (%)	F1(%)	Recall(%)	MCC	Precision(%)	Specificity (%)
HLA-I	TransHLA	**83.09**	82.56	80.06	**0.66**	**85.22**	**86.11**
	Mhcflurry	82.96	**83.24**	84.57	0.66	81.95	81.38
	Mhcnuggets	50.07	66.67	**99.87**	0.02	50.04	0.29
	TransPHLA	62.68	71.75	94.78	0.33	57.72	30.58
	Anthem	74.46	77.06	85.80	0.50	69.94	63.12
	NetMHCpan4.1b	81.08	81.97	86.01	0.62	78.29	76.15
	MixMHCpred	79.27	80.85	87.51	0.59	75.13	71.03
HLA-II	TransHLA	**66.88**	**64.08**	59.09	**0.34**	**66.99**	**74.67**
	DeepSeqPanII	49.68	49.89	50.10	-0.00	49.68	48.10
	Mhcnuggets	50.13	64.26	**89.69**	0.00	50.07	10.59
	NetMHCIIpan4.3b	60.14	62.76	67.18	0.20	58.89	53.10
	MixMHC2pred	61.62	61.79	62.07	0.23	61.52	61.17

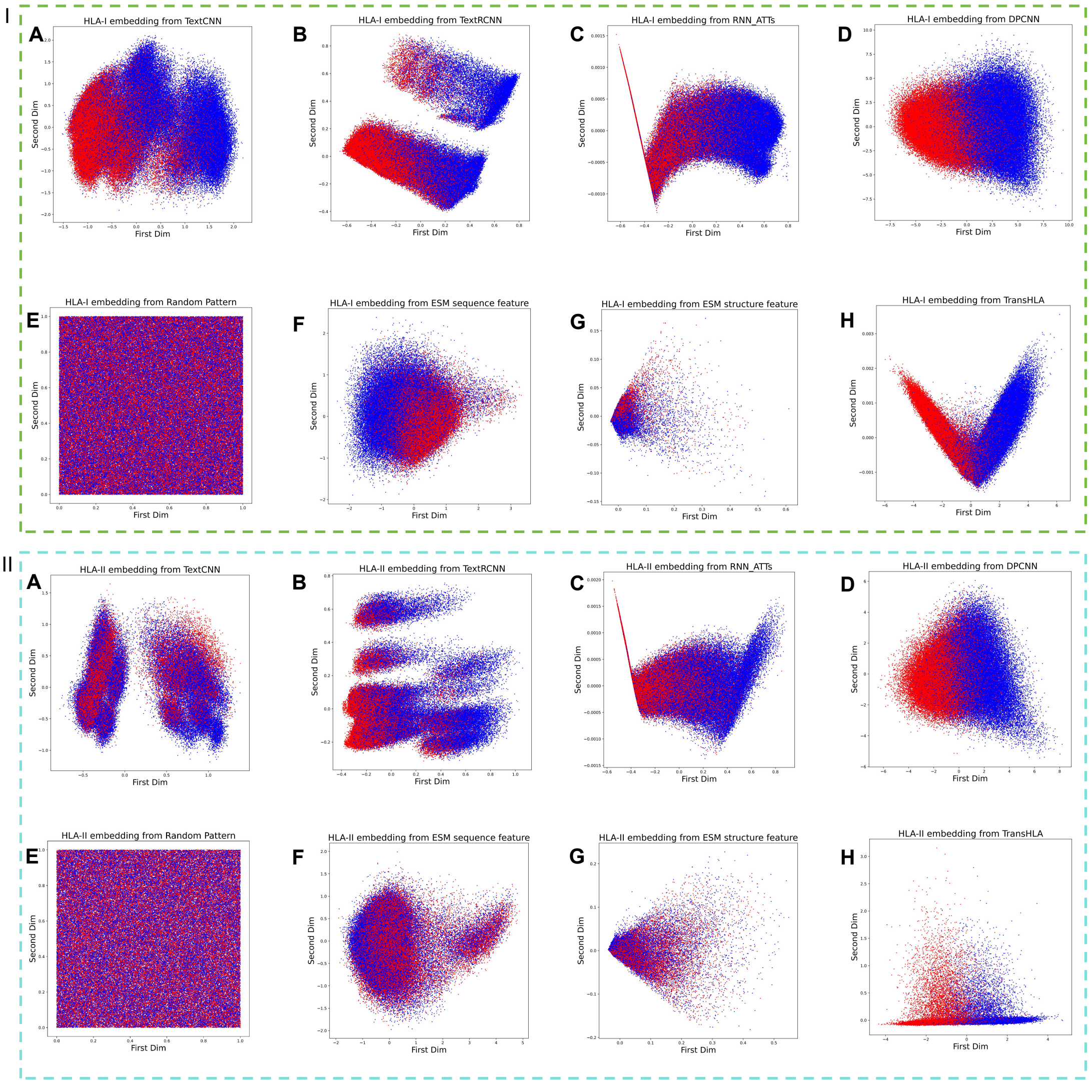

Figure 4. This figure displays the PCA-based 2D feature space distribution of HLA-I and HLA-II epitope presentation test sets. The space is split into two regions: Region I for HLA-I epitopes and Region II for HLA-II epitopes. Each region shows positive samples as blue dots and negative samples as red dots, with representations from various model embeddings (A-H) including Random Pattern, TextCNN, TextRCNN, RNN-ATTs, DPCNN, random initial, ESM2 pre-trained sequence, structure pre-trained, and TransHLA.

demonstrates a more robust capability in filtering out noise during epitope identification. In the prediction of HLA-II binding, TransHLA showed better performance than other models, achieving the highest scores in ACC (66.88%), F1 (64.08%), MCC (0.34), Precision (66.99%), and Specificity (74.67%). In contrast, while Mhcnuggets achieved a high recall of 89.69% in HLA-II binding, its precision was only 50.04%, indicating that it misjudged a large number of negative samples as epitopes. This causes a lot of noise to be mixed into the screening results. NetMHCIIpan 4.3b and MixMHC2pred achieved ACC scores of 60.34% and 61.62%, respectively on HLA-II, but they have much lower Specificity scores than TransHLA. DeepSeqPanII performs worse than the other models in HLA-II binding with 49.68% ACC. To further validate TransHLA's robustness in real-world scenarios with more negative than positive samples, we conducted an experiment with a 1:4 positive-to-negative sample ratio. TransHLA achieved an ACC of 84.75% and Specificity of 86.11% for HLA-I binding, and an ACC of 71.14% and Specificity of 74.12% for HLA-II. Detailed results are in Supplementary Table 2.

TransHLA extracts a high-quality peptide embedding in low-dimension

To assess the feature extraction capability of the model, we undertook dimension reduction and visualization of the penultimate layer features derived from the self-trained models outlined in test data, along with random embeddings and the TransHLA embeddings. The PCA [41] layouts of the learned representations for HLA-I epitopes binding prediction (Figure 4).

When comparing with random embedding, both DPCNN (Figure 4 I-D, II-D) and TransHLA (Figure 4 I-H, II-H)demonstrate superior embedding effects on HLA-I and HLA-II binding epitopes, thereby exhibiting distinct discrimination between positive and negative samples. However, in the low-dimensional visualization of DPCNN, a considerable number of positive and negative samples overlap at the junction, while TransHLA exhibits a more apparent boundary in comparison.

Table 4. Comparison Results with other HLA-epitope binding software in NeoEpitope Prediction Case Study

Type	Method	ACC (%)	F1(%)	Recall(%)	MCC	Precision(%)	Specificity (%)
HLA-I	TransHLA	**90.65**	**90.91**	93.52	**0.81**	**88.43**	**87.77**
	MHCflurry	89.56	90.23	96.40	0.80	84.81	82.73
	Mhcnuggets	50.00	66.67	**100.00**	0.00	50.00	0.00
	TransPHLA	64.03	73.40	99.28	0.40	58.23	28.78
	Anthem	82.01	84.08	94.96	0.66	75.43	69.06
	NetMHCpan4.1b	85.25	86.98	98.56	0.73	77.84	71.94
	MixMHCpred	84.89	86.36	95.68	0.71	78.70	74.10

Table 5. Ablation Study on different modules

Type	Method	ACC (%)	F1(%)	Recall	MCC	AUC (%)
HLA-I	TransHLA	**84.72**	84.59	83.92	**0.69**	**91.95**
	w/ Transformer module	84.18	83.89	82.39	0.68	91.26
	w/ CNN module	83.43	83.09	84.85	0.67	90.70
	w/ structure pre-trained embedding	83.78	**84.80**	81.85	0.68	91.39
	w/ sequence pre-trained embedding	73.37	73.45	73.56	0.4782	80.72
	w/ any embedding	84.56	84.68	**84.87**	0.68	91.33
	w/ TIM Loss	84.04	83.86	82.98	0.68	90.93
	extend sequence	75.60	75.98	77.21	0.51	83.20
HLA-II	TransHLA	**79.94**	**81.07**	**86.19**	**0.60**	**88.14**
	w/ Transformer module	79.38	79.42	79.31	0.59	87.31
	w/ CNN module	74.43	74.09	74.85	0.49	82.36
	w/ structure pre-trained embedding	79.68	80.47	79.41	0.59	87.49
	w/ sequence pre-trained embedding	67.52	61.21	51.41	0.37	76.84
	w/ any embedding	78.35	78.44	77.87	0.57	86.13
	w/ TIM Loss	79.14	78.14	74.79	0.58	86.56
	extend sequence	77.63	77.34	78.07	0.55	85.02

Good performance achieved in TCR experiments validated NeoEpitope prediction by TransHLA

To validate the effectiveness of TransHLA in NeoEpitope prediction, we employed newly identified epitopes with immunogenicity verified by TCR experiments from NEPDB [25] as positive samples and compared our software with several other widely used tools for NeoEpitope prediction, including NetMHCpan, Mhcflurry, Mhcnuggets, MixMHCpred, TransPHLA, and Anthem. In NEPDB, we collected a total of 139 neoantigens presented by HLA-I alleles. Additionally, we used a 1:1 ratio of randomly selected negative samples to form our final dataset.

The results of the comparison between these tools are presented in Table 4. Among the compared methods, TransHLA and Mhcflurry achieved the top two performances. Specifically, TransHLA attained the highest accuracy of 90.65%, followed closely by Mhcflurry, which reached an accuracy of 89.56%. Furthermore, although NetMHCpan4.1b achieved a recall of 98.56%, its specificity was only 71.94%, highlighting its tendency to misclassify negative samples as positive. In contrast, TransHLA maintained a strong balance between recall and specificity, achieving a specificity of 87.77% and a high recall of 93.52%. Interestingly, all models demonstrated higher recall in prediction compared to MHC experimental validation, with epitopes validated by TCR experiments (Table 4) performing significantly better in predictions than those validated solely by MHC experiments (Table 3). TransHLA demonstrated the highest Specificity, Precision, ACC, F1, and MCC, ensuring that it does not miss a large number of immunogenic epitopes while effectively filtering out the majority of negative samples.

Ablation study

To evaluate the contribution of different components of TransHLA to its performance on test data, we conducted an ablation study on seven variants: without the transformer module, omitting structure pre-trained embedding, removing sequence pre-trained embedding, deleting the CNN module, without any pre-trained embedding, changing TIM loss to Cross Entropy loss, and randomly extending the peptide sequence by 0-3 units on each end using proteins aligned with Diamond. The performance metrics for different modules across both HLA-I and HLA-II models are illustrated in Table 5.

Based on the comparisons conducted, it is evident that the sequence embedding approach is significantly more effective in capturing the peptide features, achieving 84.56% and 79.68% accuracy in HLA-I and HLA-II, respectively. However, the extended sequences only achieved 75.60% and 77.63% accuracy in HLA-I and HLA-II, respectively. This may be due to the fact that, after epitope extension, the distinguishing features are no longer as prominent. Epitopes are a specific type of peptide with unique overall chemical and structural properties, such as hydrophobicity, aromaticity, and secondary structure. Although the extended sequences include the original fragments, they can be considered as new peptide fragments that may no longer retain the original characteristics of the epitope, thus potentially confusing the model [42, 43, 44].

The CNN modules, due to their difference in global feature extraction compared to pretrained transformers, contribute substantially to performance enhancement, achieving 84.18% and 79.38% accuracy in HLA-I and HLA-II. Additionally, other modules also play their respective roles in the overall efficacy of the system. This is corroborated by ablation studies, which demonstrate that each module contributes positively to the model's predictive capabilities.

Chemical and Secondary Structure Patterns of Epitopes in Antigen Presentation

We further utilized Biopython [45] to investigate the chemical properties and secondary structure of the peptide segments. The analyzed chemical properties included aromaticity, hydrophobicity, flexibility, instability index, isoelectric point, average molecular weight, and mean charge at pH 7. For flexibility, we used a window size of 9-mer length [46] with a sliding step of 1, and averaged the resulting values. For peptides with a length less than 9, a flexibility value of 0 was assigned. Secondary structure analysis covered helix, sheet, and coil content. Using XGBoost [47] for feature selection on the same dataset as TransHLA, we identified the top three features for HLA-I as helix content, aromaticity, and flexibility, while for HLA-II, the top three were aromaticity, helix content, and hydrophobicity (Supplementary Table 1). We further analyzed these features in epitopic vs. non-epitopic regions and compared feature distributions between true positives and true negatives predicted by TransHLA.

Through rigorous statistical analyses conducted on our test dataset we made some intriguing observations regarding the helix content of epitopes and non-epitopes. For helix content feature, our findings demonstrate that epitopes consistently exhibit higher helix content in comparison to non-epitopes (Figure 5 A left, C left) with a statistically significant p-value of less than 1×10^{-6}. Furthermore, we also examined the helix content of true positive and true negative samples predicted by TransHLA and found a similar trend (Figure 5 A, C) with a p-value of less than 1×10^{-6} (Figure 5 A right, C right). Aromaticity exhibits a similar pattern to helix content, where epitopes are significantly higher than non-epitopes(Supplementary Figures 4 A, C). Upon conducting a comparative analysis of Figure 5 B and D, a notable divergence was observed when testing the model on the case study dataset. Specifically, epitopes associated with HLA Class I consistently exhibited their characteristic higher helix content, as evidenced by a statistically significant p-value of less than 1×10^{-6}. Conversely, in the case of epitopes associated with HLA Class II, this characteristic was not maintained, as indicated that the helix content of the epitopes is lower than non-epitopes. Consequently, the performance of TransHLA declined compared to the benchmark reported in test data. Moreover, from Supplementary Figure 4 B and D, we can see that aromaticity, which is related to structural stability[48, 49], following a similar trend with helix content Feature. In Supplementary Figures 3C and D, HLA II epitopes show lower hydrophobicity compared to HLA I epitopes and non-epitopes, aligning with their role in presenting exogenous antigens in aqueous environments. This hydrophilicity facilitates hydrogen bonding with water, stabilizing peptide structures [50, 51, 52]. As for flexibility, due to the special handling of this feature, we only calculated this feature for peptides 10 residues or longer, and found it to have limited statistical utility (Supplementary Figure 2). Flexibility showed higher importance in HLA-I epitopes due to shorter peptides (8-9 residues) being assigned a value of zero, artificially inflating its relevance.

Further exploring the positive epitope samples, which include True Positives (TP) and False Negatives (FN), as well as the negative epitope samples, which include True Negatives (TN) and False Positives (FP), we made an intriguing observation (Figure 5 E, F). There is a significant distribution gap in helix content between the correctly classified samples (TP and TN) and the incorrectly classified samples (FP and FN) predicted by TransHLA ($p - value \leq 1 \times 10^{-6}$). Interestingly, the incorrectly classified samples (FN and FP) exhibit relatively similar flexibility distributions. This similarity in epitope characteristics between the FN and FP samples likely contributes to the high difficulty in accurate prediction. Similarly, from Supplementary Figures 3, and 4 (E, F), we can observe that Hydrophobicity, and Aromaticity, exhibit similar conclusions.

Statistical data indicates that both HLA-I and HLA-II epitopes are more rigid and stable, supporting the notion that rigidity facili-

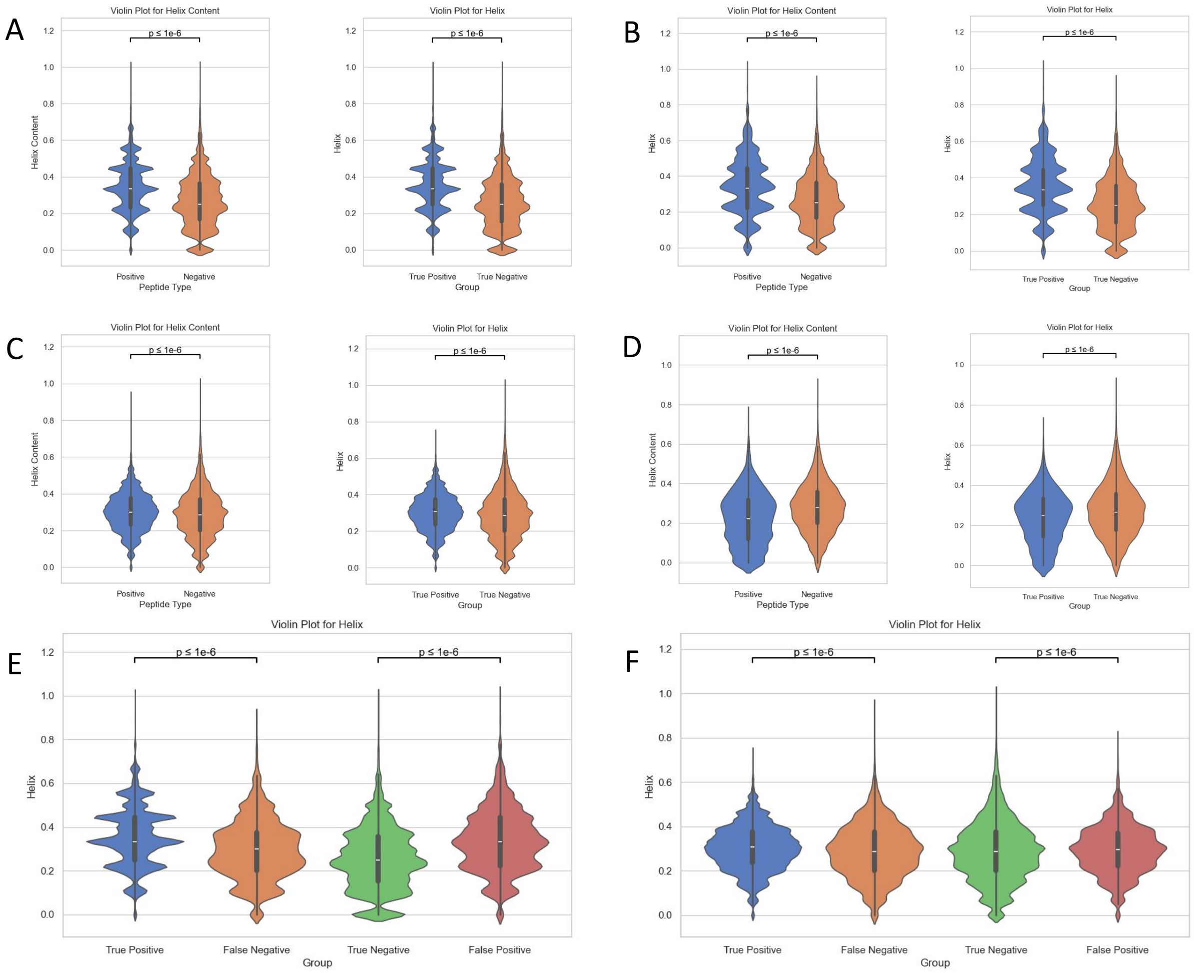

Figure 5. This comprehensive figure presents a series of violin plots illustrating the 'helix content' structure property of peptides across various sample subsets for HLA-I and HLA-II molecules. Subfigure (A) delineates the helix content distribution in independent test samples for HLA-I, separated into positive and negative samples, with each subgroup's statistical significance assessed via t-tests and annotated with corresponding p-values. Subfigure (C) mirrors this setup for HLA-II independent test samples, highlighting the comparative helix content distributions. The external dataset distributions for HLA-I and HLA-II are respectively showcased in subfigures (B) and (D), emphasizing the metric's external validity. Subfigures (E) and (F) delve deeper, contrasting the helix content of true positives and false negatives against true negatives and false positives within HLA-I and HLA-II datasets, respectively.

tates epitope recognition [48, 49, 53, 54]. Our model shows that peptide segments with higher helix content and aromaticity are more likely to be recognized as epitopes in both classes. A key distinction lies in hydrophobicity—HLA-I epitopes are more hydrophobic, while HLA-II epitopes are more hydrophilic, likely reflecting their roles in presenting intracellular and extracellular antigens, respectively, with the latter often exposed to aqueous environments.

Conclusion and Discussion

In this study, we introduced TransHLA, a pre-trained language model-based deep learning framework for predicting epitopes presented by both HLA-I and HLA-II. TransHLA uses a large language model to extract structural and text features of candidate sequences and then processes these features using Convolutional Neural Networks (CNN) and Transformer modules. Experimental results on the benchmark dataset show that TransHLA outperforms cutting edge sequence classification models in predicting both HLA-I and HLA-II binding epitopes.

In comparison to traditional epitope-HLA binding methods, which rely on both epitope and HLA allele information, TransHLA allows users to perform epitope screening without requiring HLA alleles as input. While HLA typing can be accurately performed using RNA-seq or WES data in personalized medicine contexts, TransHLA is designed to streamline allele-specific binding predictions during the screening of immunogenic epitopes, serving as a preliminary step for the widely used tools that focus on HLA-epitope binding affinity. When discovering new epitopes, we observed that binding affinity models tend to more easily misclassify ordinary peptide sequences as epitopes as the number of input alleles increases. This phenomenon arises because, despite binding affinity models being trained on benchmark datasets, the inclusion of multiple alleles introduces increased sequence diversity and variability in binding motifs or features, leading to higher uncertainty and reduced prediction accuracy. Additionally, for certain alleles with a disproportionately low number of epitopes available for training or other limitations, the insufficient training further exacerbates this issue. For example, in netMHCpan4.1, the allele HLA-A03:02 has only 13 epitopes available for training, resulting in an AUC of just 0.6331. As a result of these challenges, these models often misidentify non-presented negative samples as potential epitopes, which increases the experimental burden and adds additional costs for epitope discovery. This limitation is particularly problematic in scenarios such as selecting antigens for a population-wide vaccination, where the population contains many different alleles. In such cases, the variability across alleles further complicates the ability of binding affinity models to reliably identify epitopes. From Table 3 and Table 4, it is evident that binding affinity prediction software achieves significantly lower specificity in epitope detection tasks compared to TransHLA, further highlighting the limitations of these models. TransHLA effectively addresses this issue by adopting a novel training strategy: combining all epitopes into a single class as positive samples and using a 1:1 ratio of negative samples for training. This approach allows the predictor to learn the unified features of epitopes, enabling it to better distinguish epitopes from ordinary peptide sequences. By leveraging this strategy, TransHLA not only retains most potential epitopes while filtering out non-presented negatives, significantly reducing the need for extensive chemical experiments and lowering costs, but also proves particularly advantageous in tasks like population-wide antigen selection by improving the robustness and reliability of epitope detection.

In conclusion, TransHLA serves as a powerful complementary tool that expedite the precise screening of epitopes. TransHLA efficiently filters out non-epitope sequences and achieves higher accuracy compared to conventional methods. In a general Neoepitope dataset verified by TCR experiments, TransHLA achieves an accuracy of 90.65% for HLA-I epitopes.

Availability of source code and requirements

- Project name: TransHLA
- Project home page: `https://github.com/SkywalkerLuke/TransHLA`
- RRID: SCR _ 026171
- BioTools ID: biotools: transhla
- Operating system(s): Platform independent
- Programming language: Python
- Other requirements: Python 3.9 or higher, pytorch 2.0 or higher
- License: MIT license

The datasets and used in this paper are collected from IEDB, CEDAR, VDJdb, ImmunoCode, dbpepneo2.0 and NEPdb [20, 21, 22, 23, 24, 25]. And an archival copy of the code and supporting data is available via the GigaScience repository.[55]. We also submit the training data and test data along with our source code of TransHLA. The alleles used by the softwares for predicting HLA-binding affinity are also provided in the GigaScience Database. DOME-ML (Data, Optimization, Model and Evaluation in Machine Learning) annotations are available in the DOME registry via accession peuywb6nkx[56].

Competing interests

The authors declare no competing interests.

Author's Contributions

TCL and XYW designed the study and algorithm, implemented the code, and wrote the manuscript. SCL supervised the project, designed the initial framework, and revised the manuscript. NW implemented the tests and discussed the project. MZH discussed the project. All authors read and approved the final manuscript.

Acknowledgments

References

1. Chaffey N, Alberts, B., Johnson, A., Lewis, J., Raff, M., Roberts, K. and Walter, P. Molecular biology of the cell. 4th edn. Oxford University Press; 2003.
2. Murphy K, Weaver C. Janeway's immunobiology. Garland science; 2016.
3. Abbas A, Lichtman A, Pillai S. Cellular and molecular immunology E-book. Elsevier Health Sciences; 2014.
4. Shao XM, Bhattacharya R, Huang J, Sivakumar IA, Tokheim C, Zheng L, et al. High-throughput prediction of MHC class I and II neoantigens with MHCnuggets. Cancer immunology research 2020;8(3):396–408.
5. O'Donnell TJ, Rubinsteyn A, Laserson U. MHCflurry 2.0: improved pan-allele prediction of MHC class I-presented peptides by incorporating antigen processing. Cell systems 2020;11(1):42–48.
6. Liu Z, Jin J, Cui Y, Xiong Z, Nasiri A, Zhao Y, et al. DeepSeqPanII: an interpretable recurrent neural network model with attention mechanism for peptide-HLA class II binding prediction. IEEE/ACM Transactions on Computational Biology and Bioinformatics 2021;19(4):2188–2196.
7. Nguyen AT, Szeto C, Gras S. The pockets guide to HLA class I molecules. Biochemical Society Transactions 2021;49(5):2319–2331.
8. Mei S, Li F, Xiang D, Ayala R, Faridi P, Webb GI, et al. Anthem: a user customised tool for fast and accurate prediction of binding between peptides and HLA class I molecules. Briefings in Bioinformatics 2021;22(5):bbaa415.

9. Reynisson B, Alvarez B, Paul S, Peters B, Nielsen M. NetMHCpan-4.1 and NetMHCIIpan-4.0: improved predictions of MHC antigen presentation by concurrent motif deconvolution and integration of MS MHC eluted ligand data. Nucleic acids research 2020;48(W1):W449–W454.
10. Chu Y, Zhang Y, Wang Q, Zhang L, Wang X, Wang Y, et al. A transformer-based model to predict peptide–HLA class I binding and optimize mutated peptides for vaccine design. Nature Machine Intelligence 2022;4(3):300–311.
11. Tadros DM, Racle J, Gfeller D. Predicting MHC-I ligands across alleles and species: How far can we go? bioRxiv 2024;p. 2024–05.
12. Racle J, Guillaume P, Schmidt J, Michaux J, Larabi A, Lau K, et al. Machine learning predictions of MHC-II specificities reveal alternative binding mode of class II epitopes. Immunity 2023;56(6):1359–1375.
13. Vaswani A, Shazeer N, Parmar N, Uszkoreit J, Jones L, Gomez AN, et al. Attention is all you need. Advances in neural information processing systems 2017;30.
14. He K, Zhang X, Ren S, Sun J. Deep residual learning for image recognition. In: Proceedings of the IEEE conference on computer vision and pattern recognition; 2016. p. 770–778.
15. Lin Z, Akin H, Rao R, Hie B, Zhu Z, Lu W, et al. Evolutionary-scale prediction of atomic-level protein structure with a language model. Science 2023;379(6637):1123–1130.
16. Kim Y. Convolutional neural networks for sentence classification. arXiv preprint arXiv:14085882 2014;.
17. Lai S, Xu L, Liu K, Zhao J. Recurrent convolutional neural networks for text classification. In: Proceedings of the AAAI conference on artificial intelligence, vol. 29; 2015. .
18. Johnson R, Zhang T. Deep pyramid convolutional neural networks for text categorization. In: Proceedings of the 55th Annual Meeting of the Association for Computational Linguistics (Volume 1: Long Papers); 2017. p. 562–570.
19. Zhou P, Shi W, Tian J, Qi Z, Li B, Hao H, et al. Attention-based bidirectional long short-term memory networks for relation classification. In: Proceedings of the 54th annual meeting of the association for computational linguistics (volume 2: Short papers); 2016. p. 207–212.
20. Vita R, Mahajan S, Overton JA, Dhanda SK, Martini S, Cantrell JR, et al. The immune epitope database (IEDB): 2018 update. Nucleic acids research 2019;47(D1):D339–D343.
21. Koşaloğlu-Yalçın Z, Blazeska N, Vita R, Carter H, Nielsen M, Schoenberger S, et al. The cancer epitope database and analysis resource (CEDAR). Nucleic Acids Research 2023;51(D1):D845–D852.
22. Shugay M, Bagaev DV, Zvyagin IV, Vroomans RM, Crawford JC, Dolton G, et al. VDJdb: a curated database of T-cell receptor sequences with known antigen specificity. Nucleic acids research 2018;46(D1):D419–D427.
23. Nolan S, Vignali M, Klinger M, Dines JN, Kaplan IM, Svejnoha E, et al. A large-scale database of T-cell receptor beta (TCRβ) sequences and binding associations from natural and synthetic exposure to SARS-CoV-2. Research square 2020;.
24. Lu M, Xu L, Jian X, Tan X, Zhao J, Liu Z, et al. dbPepNeo2. 0: A database for human tumor neoantigen peptides from mass spectrometry and TCR recognition. Frontiers in Immunology 2022;13:855976.
25. Xia J, Bai P, Fan W, Li Q, Li Y, Wang D, et al. NEPdb: a database of T-cell experimentally-validated neoantigens and pan-cancer predicted neoepitopes for cancer immunotherapy. Frontiers in Immunology 2021;12:644637.
26. Buchfink B, Xie C, Huson DH. Fast and sensitive protein alignment using DIAMOND. Nature methods 2015;12(1):59–60.
27. Pruitt KD, Tatusova T, Maglott DR. NCBI reference sequences (RefSeq): a curated non-redundant sequence database of genomes, transcripts and proteins. Nucleic acids research 2007;35(suppl_1):D61–D65.
28. Fu L, Niu B, Zhu Z, Wu S, Li W. CD-HIT: accelerated for clustering the next-generation sequencing data. Bioinformatics 2012;28(23):3150–3152.
29. Chen B, Cheng X, Li P, Geng Ya, Gong J, Li S, et al. xTrimoPGLM: unified 100B-scale pre-trained transformer for deciphering the language of protein. arXiv preprint arXiv:240106199 2024;.
30. Elnaggar A, Heinzinger M, Dallago C, Rehawi G, Yu W, Jones L, et al. ProtTrans: Towards Cracking the Language of Lifes Code Through Self-Supervised Deep Learning and High Performance Computing. IEEE Transactions on Pattern Analysis and Machine Intelligence 2021;p. 1–1.
31. Brown TB, Mann B, Ryder N, Subbiah M, Kaplan J, Dhariwal P, et al. Language Models are Few-Shot Learners. Advances in Neural Information Processing Systems 2020;33:1877–1901.
32. Du Z, Ding X, Xu Y, Li Y. UniDL4BioPep: a universal deep learning architecture for binary classification in peptide bioactivity. Briefings in Bioinformatics 2023;24(3):bbad135.
33. Xu Z, Zhong H, He B, Wang X, Lu T. PTransIPs: Identification of Phosphorylation Sites Enhanced by Protein PLM Embeddings. IEEE Journal of Biomedical and Health Informatics 2024;.
34. Jumper J, Evans R, Pritzel A, Green T, Figurnov M, Ronneberger O, et al. Highly accurate protein structure prediction with AlphaFold. Nature 2021;596(7873):583–589.
35. Mirdita M, Schütze K, Moriwaki Y, Heo L, Ovchinnikov S, Steinegger M. ColabFold: making protein folding accessible to all. Nature methods 2022;19(6):679–682.
36. Devlin J, Chang MW, Lee K, Toutanova K. Bert: Pre-training of deep bidirectional transformers for language understanding. arXiv preprint arXiv:181004805 2018;.
37. Boudiaf M, Masud ZI, Rony J, Dolz J, Piantanida P, Ayed IB, Transductive Information Maximization For Few-Shot Learning; 2020.
38. Racle J, Michaux J, Rockinger GA, Arnaud M, Bobisse S, Chong C, et al. Robust prediction of HLA class II epitopes by deep motif deconvolution of immunopeptidomes. Nature biotechnology 2019;37(11):1283–1286.
39. Nagler A, Kalaora S, Barbolin C, Gangaev A, Ketelaars SL, Alon M, et al. Identification of presented SARS-CoV-2 HLA class I and HLA class II peptides using HLA peptidomics. Cell Reports 2021;35(13).
40. Yang Y, Wei Z, Cia G, Song X, Pucci F, Rooman M, et al. MHCII-peptide presentation: an assessment of the state-of-the-art prediction methods. Frontiers in Immunology 2024;15:1293706.
41. Maćkiewicz A, Ratajczak W. Principal components analysis (PCA). Computers & Geosciences 1993;19(3):303–342.
42. Westhof E, Altschuh D, Moras D, Bloomer A, Mondragon A, Klug A, et al. Correlation between segmental mobility and the location of antigenic determinants in proteins. Nature 1984;311(5982):123–126.
43. Kim DG, Choi Y, Kim HS. Epitopes of protein binders are related to the structural flexibility of a target protein surface. Journal of Chemical Information and Modeling 2021;61(4):2099–2107.
44. Klatt MG, Mack KN, Bai Y, Aretz ZE, Nathan LI, Mun SS, et al. Solving an MHC allele–specific bias in the reported immunopeptidome. JCI insight 2020;5(19).
45. Cock PJ, Antao T, Chang JT, Chapman BA, Cox CJ, Dalke A, et al. Biopython: freely available Python tools for computational molecular biology and bioinformatics. Bioinformatics 2009;25(11):1422.
46. Vihinen M, Torkkila E, Riikonen P. Accuracy of protein flexibility predictions. Proteins: Structure, Function, and Bioinformatics 1994;19(2):141–149.
47. Chen T, Guestrin C. Xgboost: A scalable tree boosting system. In: Proceedings of the 22nd acm sigkdd international conference on knowledge discovery and data mining; 2016. p. 785–794.
48. Mariño Pérez L, Ielasi FS, Bessa LM, Maurin D, Kragelj J, Black-

ledge M, et al. Visualizing protein breathing motions associated with aromatic ring flipping. Nature 2022;602(7898):695–700.
49. Anjana R, Vaishnavi MK, Sherlin D, Kumar SP, Naveen K, Kanth PS, et al. Aromatic-aromatic interactions in structures of proteins and protein-DNA complexes: a study based on orientation and distance. Bioinformation 2012;8(24):1220.
50. Baker E. Hydrogen bonding in biological macromolecules 2012;.
51. Grimaldi J, Radhakrishna M, Kumar SK, Belfort G. Stability of proteins on hydrophilic surfaces. Langmuir 2015;31(3):1005–1010.
52. Drelich J, Chibowski E, Meng DD, Terpilowski K. Hydrophilic and superhydrophilic surfaces and materials. Soft Matter 2011;7(21):9804–9828.
53. Perticaroli S, Nickels JD, Ehlers G, O'Neill H, Zhang Q, Sokolov AP. Secondary structure and rigidity in model proteins. Soft Matter 2013;9(40):9548–9556.
54. Mamonova TB, Glyakina AV, Galzitskaya OV, Kurnikova MG. Stability and rigidity/flexibility—Two sides of the same coin? Biochimica et Biophysica Acta (BBA)-Proteins and Proteomics 2013;1834(5):854–866.
55. Tianchi L, Xueying W, Wan N, Huo M, Shuaicheng L, Supporting data for "TransHLA: A Hybrid Transformer Model for HLA-Presented Epitope Detection". GigaScience Database; 2024. http://gigadb.org/dataset/102633.
56. Registry D; 2024. https://registry.dome-ml.org/review/peuywb6nkx.
