# Supplementary material for: TransHLA: a Hybrid Transformer model for HLA-presented epitope detection
Source: Gigascience. 2025 Feb 27;14:giaf008. doi: 10.1093/gigascience/giaf008 (PMC11878767; doi:10.1093/gigascience/giaf008)
Supplement: giaf008_Response_to_Reviewer_Comments_Original_Submission [file giaf008_response_to_reviewer_comments_original_submission.pdf]

Author's Response To Reviewer Comments

Dear Dr. Hans Zauner and all reviewers,
We sincerely appreciate your thorough review of our manuscript titled “TransHLA: A Hybrid Transformer Model for HLA-Presented Epitope Detection” Your insightful comments have been invaluable in guiding our revisions and enhancing the quality of our work. We have carefully considered each suggestion, reanalyzed the data, and addressed any typographical errors. We hope this edition and the reply below will address your concerns.

Reviewer 1
In this manuscript, the authors present TransHLA, a hybrid transformer model that integrates a transformer-based language model with a deep Convolutional Neural Network (CNN) module. The transformer encoder module leverages a pre-trained large language model (Evolutionary Scale Modeling - ESM2) to extract global features using a multi-head attention mechanism. The feature extraction is further enhanced by two consecutive CNN modules, maximizing the mutual information between query features (sequences) and their label predictions (epitope/non-epitope) through a modified Transductive Information Maximization (TIM) loss function. TransHLA is designed to collectively consider all HLA sites across all alleles and is the first neoantigen prediction tool of its kind, since it does not require HLA alleles as input. The authors also present benchmark study results, showcasing the increased predictive accuracy of TransHLA and its potential as a valuable pre-screening tool.

We thank the positive comments on our work.

Introduction
1)The authors distinguish between two categories of models: those that need only epitopes as input and those that require both epitopes and HLA alleles as inputs. However, the basis for this classification is unclear. For instance, MHCNuggets and DeepSeqPanII, cited as examples of the first category, actually require both an allele and an epitope to predict neoantigens. This is supported by the algorithms' manuals and the supplementary material provided by the authors, where they specify the need for HLA alleles to execute the commands.

Thanks for your careful reading and insightful comments. We have noticed the unclear classification of methods and the inconsistency with the supplementary materials. We revisited the manuals for all relevant tools, including MixMHCpred, Anthem, TransPHLA, and MixMHC2pred, and categorized them into two groups: the first category includes models that are trained individually for specific alleles, requiring users to input a peptide and select a specific allele. The second category involves a general model trained for pan-alleles, which requires users to input a peptide along with any HLA allele sequence. Both categories necessitate simultaneous input of peptides and alleles. We have confirmed that Mhcflurry, NetMHCpan, NetMHCIIpan, DeepSeqpanII, TransPHLA, MixMHCpred and MixMHC2pred belong to the first category, while Mhcnuggets, and Anthem fall into the second category. The manuscript （Lines 17 -34 in pages 1-2) and the supplementary materials have been revised for consistency.

2)The authors state: "Considering that TransHLA is the first epitope prediction software that does not impose restrictions on HLA alleles". This needs clarification, as all available "pan-allele" models do not impose restrictions on HLA alleles (the models are trained on nearly all sequenced HLAs). Perhaps the authors meant that TransHLA does not require HLA alleles as input?

Thank you for your valuable suggestion. We apologize for the lack of clarity in our statement regarding TransHLA. We have updated the text to specify that TransHLA does not require HLA alleles as input.（Lines 36-42 in page 2).

Results
3)The reason for conducting two separate benchmarks (case study and validation) with different HLA binding affinity predictors is unclear. For instance, it is not explained why netMHCpan/netMHCpanII were

not included in the first benchmark and only used in the validation part.

Thank you for your insightful comment regarding the benchmarks. We apologize for the oversight regarding the inclusion of netMHCpan/netMHCpanII in all validation benchmarks. We appreciate your suggestion and have updated the manuscript to include netMHCpan/netMHCpanII in the all benchmarks. The results are now presented in the updated Table 3 and Table 4.

4)It would be very informative if the authors were able to include other widely used HLA binding affinity predictors in their benchmarks, such as mixMHCpred and MixMHCpred2.

Thank you for your insightful suggestion. We agree that MixMHCpred and MixMHC2pred are valuable tools in the field and have included both in our benchmarks. The updated results can be found in Section Comparison Results with other HLA-epitope binding software in the case and Section Good performance achieved in TCR experiments validated NeoEpitope prediction by TransHLA (lines 380 to 390, lines 420-422 and Table 3,4).

5)"the details information of alleles used in each tool can be found in the Supplementary File" However, no information about the alleles used in this study is provided (or at least it was not made available to me at the time of reviewing this version of the manuscript).

Thank you for bringing this to our attention, and we apologize for the inconvenience. In the latest version of the manuscript, we have organized the information regarding the alleles used in each tool. The alleles used by the software for predicting HLA-binding affinity are also provided in the Google Drive linked in our data availability section.

6)The "protein structural flexibility" should be briefly explained and properly cited (Vihinen et al., 1994, Proteins, 19(2), 141-149).

Thank you for your suggestion regarding the explanation of "protein structural flexibility." We have added a brief explanation and properly cited Vihinen et al. (1994, Proteins, 19(2), 141-149) in the revised manuscript (Lines 472- 473).

Conclusion and Discussion

7)The authors claim that TransHLA alleviates "the restrictive requirement of knowing the specific HLA alleles." However, this is not typically a restriction, as serological typing of HLA is routinely performed in clinics, and samples usually come with relevant metadata. Additionally, HLA typing can be easily performed with RNAseq and/or WES data, the same data usually required to produce the putative epitopes initially, with high accuracy (e.g., OptiType can reach 93.5% [CI95: 91.8-95.1%] accuracy for HLA class I). Therefore, this information is generally readily available for processing. While the authors effectively demonstrate the accuracy of TransHLA, they fail to clarify the context in which this computational tool could be utilized. To the best of my knowledge, in the research field of personalized medicine, neoantigen vaccines are typically produced at the patient level, taking the patients' HLA alleles into consideration. Binding affinity, by definition, can quantitatively differentiate between strong (low IC50) and weak (high IC50) binders. Thus, binding affinity predictions are a pivotal step for neoantigen prioritization. Given that the authors suggest TransHLA as an "alternative for filtering potential epitopes", how would TransHLA perform in such situations? To enhance clarity, the authors should elaborate on a scenario where TransHLA would be a superior choice compared to high-performing HLA binding affinity predictors in this context.

Thank you for your thoughtful and detailed comments. We appreciate your insights regarding the context in which TransHLA can be utilized. To clarify, we propose the scenario where TransHLA may offer advantages:
TransHLA allows users to perform epitope screening without requiring HLA alleles as input. While HLA typing can be accurately performed using RNA-seq or WES data in personalized medicine contexts, TransHLA is designed to streamline allele-specific binding predictions during the screening of immunogenic epitopes, serving as a preliminary step for the widely used tools that focus on HLA-epitope binding affinity.

When discovering new epitopes, we observed that binding affinity models tend to accumulate errors as the number of input alleles increases, often misidentifying non-presented negative samples as potential epitopes. TransHLA effectively addresses this issue by retaining most potential epitopes while filtering out non-presented negatives, reducing the need for extensive chemical experiments and lowering costs.
These updates can be found in the revised manuscript, specifically in lines 552-566 in page12.

8)The authors mention in the introduction that TransHLA can be used to "expedite the precise screening of peptides". Additionally, in their GitHub repository it is stated that TransHLA "can serve as a preliminary screening for the currently popular tools that are specific for HLA-epitope binding affinity", which is quite accurate. They might consider incorporating this concept into their concluding remarks as well.

Thank you for your constructive feedback. We apologize for the inconsistency between the introduction and our GitHub repository regarding the role of TransHLA in peptide screening. We have now aligned the language in both the manuscript and the GitHub repository to ensure consistency.

Implementation:
9）Since neoantigen prediction is typically carried out using computational pipelines, it would be very helpful if the authors could provide instructions for end-users to install the software and its dependencies in isolated (contained) computational environments. To enhance clarity, I am attaching the files I used to create these environments via Conda (transhla_env.yaml), Singularity (TransHLA.def), and Docker (Dockerfile).
Following the previous point, the authors should consider providing a CLI (similar to the "train.py" and "inference.py" scripts in their GitHub repository) to enhance the software's usability in computational pipelines. As an example, I am attaching the script I used to test the software (TransHLA.py).

Thank you for your suggestions. We have further tested the software based on the files you provided (Conda's transhla_env.yaml, Singularity's TransHLA.def, and Docker's Dockerfile) and have improved the detailed installation instructions and running scripts to enhance the software's usability in computational pipelines.
We have also provided two files, TransHLA_I.py and TransHLA_II.py, along with their corresponding CLI. Additionally, we have included examples on how to install and use our software in a Colab example, and all updates have been synced to the GitHub repository.

Minor
10)It would enhance the clarity (especially for readers who are not familiar with artificial intelligence) if the authors would briefly explain each technical term and then use the abbreviations. For example, "Evolutionary Scale Modeling (ESM2)" and so on.

Thank you for your suggestion. We apologize for any confusion. We have reviewed all technical terms and provided introductions for their first occurrence. For example, ESM2 is explained in lines 46-49 on the page2.

11)Additionally, the manuscript and its supplementary material contain several grammatical and spelling errors that need to be rectified.n

Thank you for your valuable feedback. We apologize for the typographical errors and have made the necessary corrections in the manuscript and supplementary material.

Reviewer 2

The authors present TransHLA, a deep learning tool to predict whether a peptide is an HLA binder or not. They use the ESM2 language model to create peptide embeddings for structural and sequence features and then use transformers and CNNs for the binding prediction. The article is well-written and clear. However, the authors must better justify the choice of their model and its potential application.

We thank the positive comments on our work.

1)In personalized medicine, the HLA alleles of a patient can be obtained via WES and there is no need for such a HLA agnostic binding predictor. Could you briefly outline the most important medical applications where your TransHLA predictor could be most useful?

Thank you for your thoughtful and detailed comments. We appreciate your insights regarding the context in which TransHLA can be utilized. To clarify, we propose the scenario where TransHLA may offer advantages:
TransHLA allows users to perform epitope screening without requiring HLA alleles as input. While HLA typing can be accurately performed using RNA-seq or WES data in personalized medicine contexts, TransHLA is designed to streamline allele-specific binding predictions during the screening of immunogenic epitopes, serving as a preliminary step for the widely used tools that focus on HLA-epitope binding affinity. When discovering new epitopes, we observed that binding affinity models tend to accumulate errors as the number of input alleles increases, often misidentifying non-presented negative samples as potential epitopes. TransHLA effectively addresses this issue by retaining most potential epitopes while filtering out non-presented negatives, reducing the need for extensive chemical experiments and lowering costs.
These updates can be found in the revised manuscript, specifically in lines 552-566 in page12.

2)Could you give more information about your IEDB training set? What are the frequencies of the HLA alleles, and the number of peptides per allele? How did you perform the splits into training, validation, and test sets? Were peptides from the same allele all present in all 3 sets? How does TransHLA perform for peptides binding to alleles not present in the training set compared to peptides binding to alleles present in the training set? How does the performance depend on the number of peptides of the allele in the training set? Is the model biased to these frequent alleles?

Thank you for your question. Our IEDB training set includes data from multiple HLA alleles. We did not split the data based on alleles; instead, we randomly divided the data into training, validation, and test sets. This random division minimizes the impact of allele frequency on coverage.
The distribution of alleles is concentrated among a few common types. Detailed statistics, including the number of alleles and their frequencies, are provided in Figure 2 and Supplementary Figure 5. We have also included the specific number of alleles in the Materials and Methods section, lines 113–123 on page 2.
Our data is highly consistent across the splits. We specifically analyzed alleles and epitopes that appear only in the test set but are absent from the train-validation set. For Class I epitopes, there are only four such epitopes, and for Class II epitopes, there are only two. This means that almost all peptides from the same allele are present in all three sets (train, validation, and test).
For these exceptional epitopes (those from alleles not seen during training), we used TransHLA to predict their binding affinity. Remarkably, all these epitopes were predicted as binders (positive predictions) with relatively high scores, demonstrating TransHLA's ability to generalize and make accurate predictions even for epitopes corresponding to unseen alleles. The specific Class I epitopes are: 'ALWGFFPVL', 'LDTNADKQLSF', 'WQQGLRVSF', 'ILDTAGKEEY'. The specific Class II epitopes are: ’PKYVKQNTLKLAT’ and ’ISTNIRQAGVQYSRA ’. These changes are updated
in lines 321-330 on pages 6 and 8. The Supplementary Table 3,4 provide the detail result and the epitopes' corresponding alleles.
Additionally, we analyzed the number of peptides per allele, and the detailed results are provided in Supplementary Figure 6. The relationship between the number of peptides and the model's performance is discussed in the Results section under "The prediction performance in different alleles". As shown in the results, while the number of peptides per allele does have an effect on the overall accuracy, the model still maintains a reasonable level of reliability for alleles with fewer peptides, especially for Class I epitopes.
In conclusion, while frequent alleles naturally contribute more data to the training process, TransHLA does not exhibit an overwhelming bias toward these alleles. The model demonstrates the capacity to generalize well, even for less frequent alleles or alleles absent from the training set, particularly for Class I epitopes.

3)Peptides are processed by many steps before being presented on HLA molecules. These include cleavage in the proteasome, transport via TAP to the ER, cleavage by ERADs and finally loading on the HLA complex. Why don't you perform your study on extended peptide sequences, where you take into account several

amino acids before and after the peptide termini? Like this, you could also include the other processing steps. It would be interesting to see whether this sequence extension would improve prediction

Thank you for your suggestion. We extended the sequences of all epitopes and negative samples by randomly adding 0-3 amino acids before and after the peptide termini, using protein data obtained from DIAMOND alignment. We then retrained the model and included the results in the ablation study. However, this did not yield improved results. The findings have been added to Table 5 and Supplementary Figure 1. We also included possible explanations in the ablation study, which can be found in lines 449-458 on page 10.

4)Could you compare your approach with a 'simpler' approach, where you calculate all biopython features (such as flexibility), ev. choose the n most informative ones by feature selection, and use a standard classifier such as logistic regression or XGBoost to predict the HLA binding. This method has the advantage that it tells you directly which features are most relevant.fff

Thank you for your valuable suggestion. We extracted features from Biopython, including aromaticity, hydrophobicity, flexibility, instability index, isoelectric point, average molecular weight, mean charge at pH 7, Helix Content, Sheet Content, Coil Content, and then used XGBoost for logistic regression. The importance of features is shown in Supplementary Table 1:

We found that Helix Content, Aromaticity, Flexibility, and Hydrophobicity are the most contributing features. Therefore, similar to flexibility, we conducted a significance analysis for these properties and compared their features on the test and case study datasets. The specific handling of the flexibility feature is described in detail in Section 'Chemical and Secondary Structure Patterns of Epitopes in Antigen Presentation'

5)Please provide the results of the ablation study in a table in the main text, where you compare the ablated models to the base model.

Thank you for bringing this to our attention, and we apologize for the oversight. The results of the ablation study have been moved to the main text, specifically in the result section Ablation study.

6)Could you briefly explain what the different terms in the TIM loss are and why they are important?

Thank you for your suggestion. We have added an explanation of TIM loss and its different terms, as detailed below:
We address the empirical mutual information within dataset X (comprising amino acid sequences) linked to their respective outcomes Y (which now signify epitope presentation). The first factor is the empirical conditional entropy of the outcomes given the data, denoted as H(Y|X), which reduces uncertainty in predictions for unlabeled samples, encouraging the model to output confident predictions. The next factor is the empirical marginal entropy of the outcomes, denoted as H(Y), which encourages a uniform distribution of labels, preventing bias towards any particular class. To further calibrate the binary classification process, the cross-entropy loss, indicated as CE, between the model's predictions and the actual outcomes is incorporated. By integrating these entropy terms, the TIM loss enhances the model's generalization in few-shot scenarios without the need for complex meta-learning schemes.
This information has been incorporated into lines 207-227 of the manuscript.

7）Does the flexibility depend on the length of the peptides? Peptides longer than 10 often bulge out of the binding groove, and naively one would expect them to be less stiff than peptides of length 8 or 9.

Thank you very much for your insightful question. We carefully revisited how we calculated the flexibility in our previous analysis and realized that our initial approach was indeed flawed. Specifically, in Biopython, flexibility is computed using a sliding window method, where the window size is 9, and the peptide length must be at least 10. For peptide lengths shorter than 10, the algorithm returns an empty value. In our earlier calculations, we had naively divided the flexibility list by the peptide length, which inadvertently

caused the flexibility feature to be highly correlated with the peptide length.
In this revised version, we have corrected this mistake. The flexibility feature is now calculated as the average of the flexibility list, and for peptides of length 8 or 9, we set the flexibility value to 0. This corrected flexibility feature was used in our XGBoost model, and although it still exhibits relatively high importance, it is no longer a decisive factor in the model. For this specific analysis, we only considered peptides with lengths of 10 or greater. In this case, the flexibility is derived from normalized data and is thus no longer influenced by peptide length. As a result, we did not observe any significant statistical differences in flexibility for peptides of varying lengths. Additionally, we would like to note that all of the other features used in our model are also normalized and are unrelated to the peptide length. And the analysis can be seen in the section 'Chemical and Secondary Structure Patterns of Epitopes in Antigen Presentation'.
Once again, we greatly appreciate your valuable feedback, which has helped us improve the accuracy and clarity of our analysis.

Minor:
8)In Equation 10, please define ^p_k. In the text, you use T for the number of classes, in the formulae K.

Thank you for bringing this to our attention. The necessary changes have been made in the main text (lines 228-235).
