# Supplementary material for: TransHLA: a Hybrid Transformer model for HLA-presented epitope detection
Source: Gigascience. 2025 Feb 27;14:giaf008. doi: 10.1093/gigascience/giaf008 (PMC11878767; doi:10.1093/gigascience/giaf008)
Supplement: giaf008_Response_to_Reviewer_Comments_Revision_1 [file giaf008_response_to_reviewer_comments_revision_1.pdf]

Author's Response To Reviewer Comments

Reviewer 1
The authors have clearly put in significant effort to refine their revised manuscript, substantially enhancing both its clarity and scientific quality.

The latest version of the GitHub repository has been extensively updated. The manual now includes detailed information on software installation and execution (the addition of a Google Colab example was an excellent touch). The authors have also introduced conda and Docker options, greatly simplifying the installation process. Finally, the new CLI integration makes it easier to incorporate TransHLA into streamlined workflows for neoantigen predictions.

We thank the positive comments on our work.

Reviewer 2
1)Reference for MixMHCPred [11] seems wrong. Please check all your references.
Thank you for your remind， we have revised the Reference and chek all references.

2) In the discussion you outline where your tool could be most useful. Unfortunately i do not find you arguments very convincing. You state that "we observed that binding affinity models tend to accumulate errors ... TransHLA effectively addresses this issue". This might be true but you don't provide any evidence for the former or latter statements. Since these statements are the justification for the development of your tool, you should show in the paper that they are true. Or you could add another justification, for example selecting antigens for a population wide vaccination, where the pupulation contains many different alleles.

Thank you for your suggestion, which made us aware of the lack of precision in our previous statement. We have added an explanation of why binding affinity prediction software has its limitations, along with supporting evidence. Additionally, we have discussed how TransHLA addresses this issue effectively Furthermore, we have incorporated the new suggestion you provided, including the example of selecting antigens for a population-wide vaccination, where the population contains many different alleles, and elaborated on how TransHLA's robustness helps address these challenges. (Lines 554-587 in page 12).
