# Supplementary material for: TransHLA: a Hybrid Transformer model for HLA-presented epitope detection
Source: Gigascience. 2025 Feb 27;14:giaf008. doi: 10.1093/gigascience/giaf008 (PMC11878767; doi:10.1093/gigascience/giaf008)
Supplement: giaf008_Response_to_Reviewer_Comments_Revision_2 [file giaf008_response_to_reviewer_comments_revision_2.pdf]

Author's Response To Reviewer Comments

Subject: Response to Editorial Office - GIGA-D-24-00176R2

Dear Dr. Zauner,

Thank you for your email regarding our manuscript "TransHLA: A Hybrid Transformer Model for HLA-Presented Epitope Detection" (GIGA-D-24-00176R2). We appreciate the feedback and the opportunity to revise our submission.

We are pleased to inform you that we have completed all the requests outlined in your letter:

We have included the citation for the GigaDB dataset in the reference list and the data availability section.
The new software application has been registered in the bio.tools and SciCrunch.org databases, and the corresponding identifiers have been added to the manuscript.
RRIDs for other published software tools mentioned in the methods section have been included as requested.
URLs in the main text have been moved to the bibliography and cited appropriately.
All relevant accession numbers for data have been mentioned in the data availability section.
We have removed all highlighting and tracking of changes.
A reference to the DOME-ML annotations has been added to the bibliography, along with the appropriate citation in the data availability section.
Editable source files have been submitted, including LaTeX style and bib files.
We have ensured that the revised manuscript conforms to the journal style as per the Instructions for Authors.
We believe that these changes address all the requirements, and we look forward to your feedback on the revised manuscript.

Thank you once again for your guidance and support.

Best regards,

Shuaicheng Li
