# Supplementary material for: TransHLA: a Hybrid Transformer model for HLA-presented epitope detection
Source: Gigascience. 2025 Feb 27;14:giaf008. doi: 10.1093/gigascience/giaf008 (PMC11878767; doi:10.1093/gigascience/giaf008)
Supplement: giaf008_Response_to_Reviewer_Comments_Revision_3 [file giaf008_response_to_reviewer_comments_revision_3.pdf]

Author's Response To Reviewer Comments

Dear Dr. Hans,

We appreciate the feedback provided on our manuscript. We have made the following revisions in response to your comments:

We have added a new heading "Data Availability" before the section that begins with "The datasets used in this study..." as requested.
The specified section has undergone proofreading, and we have incorporated the suggested corrections. The revised text now reads: "The datasets used in this paper are collected from IEDB, CEDAR, VDJdb, ImmunoCode, dbpepneo2.0 and NEPdb [20, 21, 22, 23, 24, 25]. An archival copy of the code and supporting information is available via the GigaScience database, GigaDB [55]. We also submitted to GigaDB the training and test data along with our source code of TransHLA. The alleles used by the software for predicting HLA-binding affinity are also provided in GigaDB. DOME-ML (Data, Optimization, Model and Evaluation in Machine Learning) annotations are available in the DOME registry via accession peuywb6nkx [56]."
We have updated reference [55] to include the permanent link.
Reference [56] has been corrected to read: Tianchi L, Xueying W, Wan N, Huo M, Shuaicheng Li. TransHLA: A Hybrid Transformer Model for HLA-Presented Epitope Detection. [DOME-ML Annotations]. DOME-ML Registry 2024. https://urldefense.com/v3/__https://registry.dome-ml.org/review/peuywb6nkx__;!!KjDnqvtInNPT!i0agEmhs5f2c-6xn78vf1UHeQDc7MIvRJjXtTKhowEM818-6OF2FSrgCa2kiVF4O5NMkHRJhDcvGVW88ZMPB$.
Furthermore, we have adjusted the submission order of our files as per your guidance:

Primary manuscript file (.tex)
Bibliography files
Optional style files
Nomenclature files
Figure files

Thank you for your attention to our revisions. We look forward to your feedback.

Yours sincerely,
Prof Shuaicheng LI
