# Supplementary material for: TransHLA: a Hybrid Transformer model for HLA-presented epitope detection
Source: Gigascience. 2025 Feb 27;14:giaf008. doi: 10.1093/gigascience/giaf008 (PMC11878767; doi:10.1093/gigascience/giaf008)
Supplement: giaf008_Reviewer_1_Report_Original_Submission — Georgios Fotakis -- 8/2/2024 [file giaf008_reviewer_1_report_original_submission.pdf]

Reviewer Report

Title: TransHLA: A Hybrid Transformer Model for HLA-Presented Epitope Detection

Version: Original Submission **Date:** 8/2/2024

Reviewer name: Georgios Fotakis

Reviewer Comments to Author:

1) General CommentsIn this manuscript, the authors present TransHLA, a hybrid transformer model that integrates a transformer-based language model with a deep Convolutional Neural Network (CNN) module. The transformer encoder module leverages a pre-trained large language model (Evolutionary Scale Modeling - ESM2) to extract global features using a multi-head attention mechanism. The feature extraction is further enhanced by two consecutive CNN modules, maximizing the mutual information between query features (sequences) and their label predictions (epitope/non-epitope) through a modified Transductive Information Maximization (TIM) loss function. TransHLA is designed to collectively consider all HLA sites across all alleles and is the first neoantigen prediction tool of its kind, since it does not require HLA alleles as input. The authors also present benchmark study results, showcasing the increased predictive accuracy of TransHLA and its potential as a valuable pre-screening tool.The computational method presented in this manuscript demonstrates a strong scientific foundation and shows promise for future refinement and extension, suggesting significant potential for meaningful research output. However, there are some conceptual and technical concerns that need to be addressed.2) Specific comments for revisiona) MajorManuscript:i) Introduction- The authors distinguish between two categories of models: those that need only epitopes as input and those that require both epitopes and HLA alleles as inputs. However, the basis for this classification is unclear. For instance, MHCNuggets and DeepSeqPanII, cited as examples of the first category, actually require both an allele and an epitope to predict neoantigens. This is supported by the algorithms' manuals and the supplementary material provided by the authors, where they specify the need for HLA alleles to execute the commands.- The authors state:"Considering that TransHLA is the first epitope prediction software that does not impose restrictions on HLA alleles"This needs clarification, as all available "pan-allele" models do not impose restrictions on HLA alleles (the models are trained on nearly all sequenced HLAs). Perhaps the authors meant that TransHLA does not require HLA alleles as input?ii) Results- The reason for conducting two separate benchmarks (case study and validation) with different HLA binding affinity predictors is unclear. For instance, it is not explained why netMHCpan/netMHCpanII were not included in the first benchmark and only used in the validation part.- It would be very informative if the authors were able to include other widely used HLA binding affinity predictors in their benchmarks, such as mixMHCpred and mixMHCpred2.- The authors state:"the details information of alleles used in each tool can be found in the Supplementary File"However, no information about the alleles used in this study is provided (or at least it was not made available to me at the time of reviewing this version of the manuscript).- The "protein structural flexibility" should be briefly explained and properly cited (Vihinen et al., 1994, Proteins, 19(2), 141-149).iii) Conclusion and Discussion- The authors claim that TransHLA alleviates "the restrictive requirement of knowing the specific HLA alleles." However, this is not typically

a restriction, as serological typing of HLA is routinely performed in clinics, and samples usually come with relevant metadata. Additionally, HLA typing can be easily performed with RNAseq and/or WES data, the same data usually required to produce the putative epitopes initially, with high accuracy (e.g., OptiType can reach 93.5% [CI95: 91.8-95.1%] accuracy for HLA class I). Therefore, this information is generally readily available for processing. While the authors effectively demonstrate the accuracy of TransHLA, they fail to clarify the context in which this computational tool could be utilized.- To the best of my knowledge, in the research field of personalized medicine, neoantigen vaccines are typically produced at the patient level, taking the patients' HLA alleles into consideration. Binding affinity, by definition, can quantitatively differentiate between strong (low IC50) and weak (high IC50) binders. Thus, binding affinity predictions are a pivotal step for neoantigen prioritization. Given that the authors suggest TransHLA as an "alternative for filtering potential epitopes", how would TransHLA perform in such situations? To enhance clarity, the authors should elaborate on a scenario where TransHLA would be a superior choice compared to high-performing HLA binding affinity predictors in this context.- The authors mention in the introduction that TransHLA can be used to "expedite the precise screening of peptides". Additionally, in their GitHub repository it is stated that TransHLA "can serve as a preliminary screening for the currently popular tools that are specific for HLA-epitope binding affinity", which is quite accurate. They might consider incorporating this concept into their concluding remarks as well.Implementation:- Since neoantigen prediction is typically carried out using computational pipelines, it would be very helpful if the authors could provide instructions for end-users to install the software and its dependencies in isolated (contained) computational environments. To enhance clarity, I am attaching the files I used to create these environments via Conda (transhla_env.yaml), Singularity (TransHLA.def), and Docker (Dockerfile).- Following the previous point, the authors should consider providing a CLI (similar to the "train.py" and "inference.py" scripts in their GitHub repository) to enhance the software's usability in computational pipelines. As an example, I am attaching the script I used to test the software (TransHLA.py).b) Minor- It would enhance the clarity (especially for readers who are not familiar with artificial intelligence) if the authors would briefly explain each technical term and then use the abbreviations. For example, "Evolutionary Scale Modeling (ESM2)" and so on.- Additionally, the manuscript and its supplementary material contain several grammatical and spelling errors that need to be rectified.

Level of Interest

Please indicate how interesting you found the manuscript: Choose an item.

Quality of Written English

Please indicate the quality of language in the manuscript: Choose an item.

Declaration of Competing Interests

Please complete a declaration of competing interests, considering the following questions:

- Have you in the past five years received reimbursements, fees, funding, or salary from an organisation that may in any way gain or lose financially from the publication of this manuscript, either now or in the future?
- Do you hold any stocks or shares in an organisation that may in any way gain or lose financially from the publication of this manuscript, either now or in the future?
- Do you hold or are you currently applying for any patents relating to the content of the manuscript?
- Have you received reimbursements, fees, funding, or salary from an organization that holds or has applied for patents relating to the content of the manuscript?
- Do you have any other financial competing interests?
- Do you have any non-financial competing interests in relation to this paper?

If you can answer no to all of the above, write 'I declare that I have no competing interests' below. If your reply is yes to any, please give details below.

I declare that I have no competing interests

I agree to the open peer review policy of the journal. I understand that my name will be included on my report to the authors and, if the manuscript is accepted for publication, my named report including any attachments I upload will be posted on the website along with the authors' responses. I agree for my report to be made available under an Open Access Creative Commons CC-BY license (http://creativecommons.org/licenses/by/4.0/). I understand that any comments which I do not wish to be included in my named report can be included as confidential comments to the editors, which will not be published.

Choose an item.

To further support our reviewers, we have joined with Publons, where you can gain additional credit to further highlight your hard work (see: https://publons.com/journal/530/gigascience). On publication of this paper, your review will be automatically added to Publons, you can then choose whether or not to claim your Publons credit. I understand this statement.

Yes Choose an item.
