# Supplementary material for: TransHLA: a Hybrid Transformer model for HLA-presented epitope detection
Source: Gigascience. 2025 Feb 27;14:giaf008. doi: 10.1093/gigascience/giaf008 (PMC11878767; doi:10.1093/gigascience/giaf008)
Supplement: giaf008_Reviewer_2_Report_Original_Submission — Markus MÃ¼ller -- 8/5/2024 [file giaf008_reviewer_2_report_original_submission.pdf]

Reviewer Report

Title: TransHLA: A Hybrid Transformer Model for HLA-Presented Epitope Detection

Version: Original Submission **Date:** 8/5/2024

Reviewer name: Markus MÃ¼ller

Reviewer Comments to Author:

The authors present TransHLA, a deep learning tool to predict whether a peptide is an HLA binder or not. They use the ESM2 language model to create peptide embeddings for structural and sequence features and then use transformers and CNNs for the binding prediction. The article is well-written and clear. However, the authors must better justify the choice of their model and its potential application.Major comments:1) In personalized medicine, the HLA alleles of a patient can be obtained via WES and there is no need for such a HLA agnostic binding predictor. Could you briefly outline the most important medical applications where your TransHLA predictor could be most useful?2) Could you give more information about your IEDB training set? What are the frequencies of the HLA alleles, and the number of peptides per allele?How did you perform the splits into training, validation, and test sets? Were peptides from the same allele all present in all 3 sets? How does TransHLAperform for peptides binding to alleles not present in the training set compared to peptides binding to alleles present in the training set?How does the performance depend on the number of peptides of the allele in the training set? Is the model biased to these frequent alleles?3) Peptides are processed by many steps before being presented on HLA molecules. These include cleavage in the proteasome, transport via TAP to the ER, cleavage by ERADs and finally loading on the HLA complex. Why don't you perform your study on extended peptide sequences, where you take into account several amino acids before and after the peptide termini? Like this, you could also include the other processing steps. It would be interesting to see whether this sequence extension would improve prediction.4) Could you compare your approach with a 'simpler' approach, where you calculate all biopython features (such as flexibility), ev. choose the n most informative ones by feature selection, and use a standard classifier such as logistic regression or XGBoost to predict the HLA binding. This method has the advantage that it tells you directly which features are most relevant.5) Please provide the results of the ablation study in a table in the main text, where you compare the ablated models to the base model.6) Could you briefly explain what the different terms in the TIM loss are and why they are important?7) Does the flexibility depend on the length of the peptides? Peptides longer than 10 often bulge out of the binding groove, and naively one would expect them to be less stiff than peptides of length 8 or 9.Minor:1) In Equation 10, please define ^p_k. In the text, you use T for the number of classes, in the formulae K.

Level of Interest

Please indicate how interesting you found the manuscript: Choose an item.

Quality of Written English

Please indicate the quality of language in the manuscript: Choose an item.

Declaration of Competing Interests

Please complete a declaration of competing interests, considering the following questions:

- Have you in the past five years received reimbursements, fees, funding, or salary from an organisation that may in any way gain or lose financially from the publication of this manuscript, either now or in the future?
- Do you hold any stocks or shares in an organisation that may in any way gain or lose financially from the publication of this manuscript, either now or in the future?
- Do you hold or are you currently applying for any patents relating to the content of the manuscript?
- Have you received reimbursements, fees, funding, or salary from an organization that holds or has applied for patents relating to the content of the manuscript?
- Do you have any other financial competing interests?
- Do you have any non-financial competing interests in relation to this paper?

If you can answer no to all of the above, write 'I declare that I have no competing interests' below. If your reply is yes to any, please give details below.

I declare that I have no competing interests

I agree to the open peer review policy of the journal. I understand that my name will be included on my report to the authors and, if the manuscript is accepted for publication, my named report including any attachments I upload will be posted on the website along with the authors' responses. I agree for my report to be made available under an Open Access Creative Commons CC-BY license (http://creativecommons.org/licenses/by/4.0/). I understand that any comments which I do not wish to be included in my named report can be included as confidential comments to the editors, which will not be published.

Choose an item.

To further support our reviewers, we have joined with Publons, where you can gain additional credit to further highlight your hard work (see: https://publons.com/journal/530/gigascience). On publication of this paper, your review will be automatically added to Publons, you can then choose whether or not to claim your Publons credit. I understand this statement.

Yes Choose an item.
